# Single dose creatine improves cognitive performance and induces changes in cerebral high energy phosphates during sleep deprivation

**DOI:** 10.1038/s41598-024-54249-9

**Published:** 2024-02-28

**Authors:** Ali Gordji-Nejad, Andreas Matusch, Sophie Kleedörfer, Harshal Jayeshkumar Patel, Alexander Drzezga, David Elmenhorst, Ferdinand Binkofski, Andreas Bauer

**Affiliations:** 1 Institute of Neuroscience and Medicine (INM-2), Molecular Organization of the Brain, Forschungszentrum Jülich, 52425 Jülich, Germany; 2 Division of Clinical Cognitive Sciences, Department of Neurology, RWTH Aachen University Hospital, 52074 Aachen, Germany; 3 Department of Nuclear Medicine, Faculty of Medicine and University Hospital Cologne, University of Cologne, 50937 Cologne, Germany; 4 German Center for Neurodegenerative Diseases (DZNE), Bonn-Cologne, Germany; 5 Institute of Neuroscience and Medicine (INM-4), Forschungszentrum Jülich, Jülich, Germany

**Keywords:** Human behaviour, Metabolism, Neurophysiology, Circadian rhythms and sleep, Cognitive neuroscience, Molecular neuroscience

## Abstract

The inverse effects of creatine supplementation and sleep deprivation on high energy phosphates, neural creatine, and cognitive performances suggest that creatine is a suitable candidate for reducing the negative effects of sleep deprivation. With this, the main obstacle is the limited exogenous uptake by the central nervous system (CNS), making creatine only effective over a long-term diet of weeks. Thus far, only repeated dosing of creatine over weeks has been studied, yielding detectable changes in CNS levels. Based on the hypothesis that a high extracellular creatine availability and increased intracellular energy consumption will temporarily increase the central creatine uptake, subjects were orally administered a high single dose of creatinemonohydrate (0.35 g/kg) while performing cognitive tests during sleep deprivation. Two consecutive ^31^P-MRS scans, ^1^H-MRS, and cognitive tests were performed each at evening baseline, 3, 5.5, and 7.5 h after single dose creatine (0.35 g/kg) or placebo during sub-total 21 h sleep deprivation (SD). Our results show that creatine induces changes in PCr/Pi, ATP, tCr/tNAA, prevents a drop in pH level, and improves cognitive performance and processing speed. These outcomes suggest that a high single dose of creatine can partially reverse metabolic alterations and fatigue-related cognitive deterioration.

## Introduction

The modern lifestyle and work pressure favor sleep deprivation (SD), leading to more accidents, reduced performance, and chronic diseases. To diminish these negative consequences, psychoactive substances such as caffeine gained immense popularity in recent decades. The sports community appreciates creatine in an ergogenic sense, to enhance physical peak performance. Protective effects in cell culture and ex vivo studies^1^ and cognitive improvements^2–4^, motivated studies of creatine supplementation in neurodegenerative diseases^5–8^. In sleep disorders and SD, changes in creatine-related metabolites were observed using ^1^H-MRS or ^31^P-MRS in humans^9–14^ and enzymatic assays or high pressure liquid chromatography (HPLC) in animals^10,15^.

Up to now, studies about a prolonged diet of oral creatine supplementation over a minimum period of 1 week observed an increase in neural total CR (tCr) and PCr, a decrease in ATP and glutamate (Glu), and improvements in cognitive performance^2,6,16–19^. All are metabolites that are inversely affected by SD and sleep disorders.

Creatine is of low water solubility, poor and delayed bioavailability, and does not diffuse passively through cell membranes. Its uptake into the CNS is assured and limited by the creatine transporter CreatT (SLC6A8) operating near saturation and expressed in the endothelial cell layer of the blood–brain barrier (BBB) but not in its layer of astrocyte feet^20^. Furthermore, the synthetic pathway comprises the arginine:glycine amidinotransferase (AGAT) followed by guanidinoacetate methyltransferase (GAMT). AGAT and GAMT were detected in all brain cell types, neurons, astrocytes, and oligodendrocytes, but rarely co-expressed in the same cell. CNS uptake of exogenous creatine from the periphery is marginal and takes a long time. Therefore, most studies investigating the effect of creatine supplementation on cerebral metabolites, require a minimum period of 1 week or longer. A study by^21^, starting with early measurements after 3 days of Cr supplementation, found no significant change in cerebral tCr level in the brain. Nevertheless, the kinetics of creatine serum levels in humans have been extensively studied also after a 20 g single oral dose, reaching a maximum at 2.5 h (*T*_max_) and decreasing to half-maximum at 5 h^22^. Perasso et al.^23^ showed the uptake of [^14^C] creatine into rat brain reaching a plateau after 2.5 h and lasting for 9 h after intraperitoneal 160 mg/kg creatine. Other studies have found increased creatine levels when supplemented in a modified form or mixed with additional components^8,24^. These results indicate that intracellular creatine uptake on a short time scale is possible under certain conditions. The study aimed to prove whether a high extracellular availability of creatine can compensate for metabolic changes and cognitive impairment during sleep deprivation.

Hence, the kinetics of cerebral phosphate-metabolites and cognitive performance has been studied in a time range of 8 h after acute single dose creatine (0.35 g/kg) versus placebo during the first two-thirds of a night without sleep using ^1^H-MRS, ^31^P-MRS, and cognitive tasks at four-time points.

## Materials and methods

### Study participants

Fifteen healthy subjects (8 females, aged 23 ± 2 years, range 20–28, 13 right handed) participated. None had signs of sleep disorders, psychiatric or neurological diseases, alcohol or drug abuse, were smokers, or took any medication. To rule out symptoms of sleep problems, a seven-item questionnaire Insomnia Severity Index (ISI) of two last weeks were performed. Caffeine and occasional alcohol intake were stopped at least 48 h before the measurement nights and not resumed in between. Subjects were requested to sleep every night at 11 p.m., wake up at 7 a.m. in the morning, and record their sleep/wake time 2 weeks before and between the sessions. The study was in accordance with the declaration of Helsinki and approved by the local ethics committee of the Medical Faculty of RWTH Aachen University. Informed consent was obtained from all subjects for the study.

### Experimental procedure

**Figure 1:**
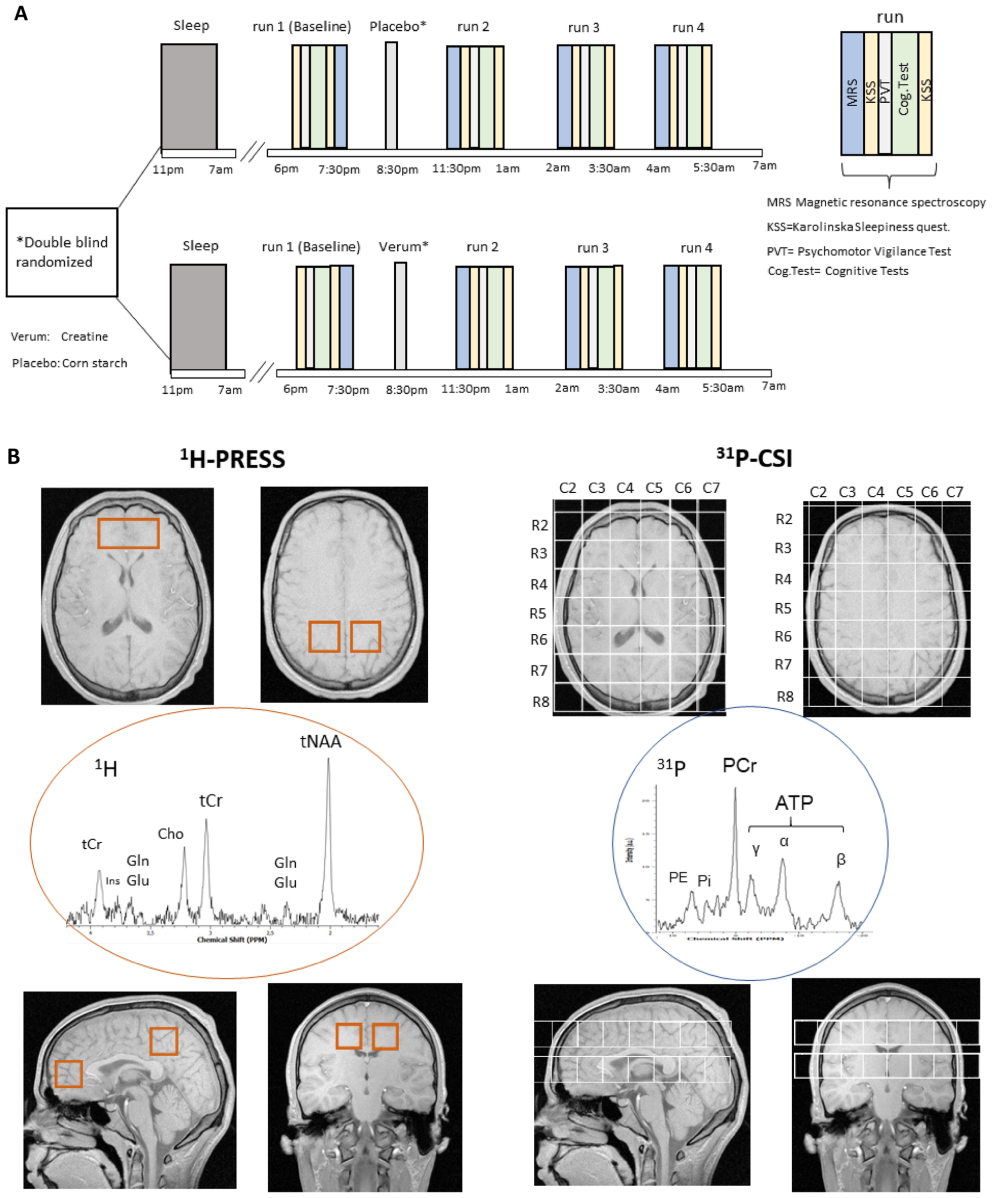
(**A**) Study design. Subjects were administered creatine at 8:30 p.m. in one session and placebo in another, spaced at least five days in random order. Seven participants completed the verum and eight the placebo as the first condition. Cognitive and metabolic parameters were acquired in four runs, baseline starting at 6 p.m., the other at 0 a.m., 2 a.m., and 4 a.m. Each session took 1 h:35 min and comprised two ^31^P-MRS-, three ^1^H-MRS measurements, followed by fatigue scores, psychomotor vigilance tests (PVT), and other cognitive tasks (Cog.Test). (**B**) Positioning of three single voxel ^1^H-MR-spectroscopy (PRESS) voxels (left, red) and two 8 × 8 ^31^P-MRS CSI grids (right, white) in coronal, transversal and sagittal view. Isotropic voxel size was (25 mm)^3^ except for the frontal PRESS-voxel of double volume. Exemplary spectra are given with some signals assigned. Signals exploited in this study were of ^31^P-MRS Pi at ≈5 ppm, PCr set at 0 ppm and the ATP-β-signal at − 16.3 ppm, of ^1^H-MRS the CH_3_-signal at 3.2 ppm, and the CH_2_-signal at 3.92 ppm of creatine (total creatine, tCr), N-acetyl-aspartate (NAA) at 2.0 ppm and signals of glutamate (Glu) at 2.35 (C3, C4-protons) and glutamine or glutamate (Glx) at 3.75 ppm (C2-proton).

This trial was a double-blind, randomized, prospective with balanced cross-over design. Verum (creatinemonohydrate, AlzChem, Trostberg) and placebo (corn starch, Caelo GmbH) were prepared, blinded, and arranged in pair-wise randomized, balanced order by the university hospital pharmacy Mainz. Every subject was measured at two nights (Fig. 1A) with a minimum interval of 5 days (max. 27, mean 10 ± 6 days) in between. The study was conducted at the University Hospital RWTH Aachen, Germany. Throughout both sessions, subjects stayed in a lab room next to the MRI scanner in a dimly lit and quiet environment under consistent and constant conditions. They were not allowed to sleep, permanently supervised, optically monitored, and spoken to whenever initial signs of falling asleep occurred. No cognitively stressful activities such as working on a laptop, watching movies, or playing games were permitted, while the consumption of drinks, and light foods such as nonmeat snacks were allowed. At one night, 0.35 g/kg creatine was administered, at the other, 0.35 g/kg placebo. A positive and negative affect schedule PANAS^25^ was acquired once initially per session. Karolinska Sleepiness Scores (KSS, ranged from 1 to 10) and fatigue score (FAT, ranged from 1 to 20) were performed before and after each run. FAT was the 10-item inverted version of the Samn and Perelli Fatigue score^26,27^.

After the baseline measurement run which started at 6:10 p.m. (± 15 min), subjects were orally administered with 0.35 g/kg creatine or 0.35 g/kg placebo at 8:28 p.m. (± 12 min) and measured in subsequent runs at 11:33 p.m. (± 14 min), 1:39 a.m. (± 14 min) and 3:44 a.m. (± 14 min), from now on referred to as 0 a.m., 2 a.m., 4 a.m. The 3 time points were chosen to follow the course effect of creatine, which according to Schedel et al.^22^, extends a time range of up to 7 h in plasma and reaches a maximum level of 3 h after injection.

Each run comprised two ^31^P-MRS measurements, three ^1^H-MRS measurements, and a neuropsychological test battery outside the scanner (Fig. 1A,B). Only in the baseline session cognitive performance tests were carried out before MRS measurements. The aim of exchanging the order at baseline was to prevent a cognitive performance associated fatigue compensation in metabolic changes when creatine was orally administered. Including preparing, adjustments and shimming, the average duration of a MRS measurement was 1 h:15 min (± 10 min). Subjects had to fix a spot with open eyes in the scanner, were equipped with a pulse oximeter, and were permanently monitored. After MRS, subjects did cognitive tasks lasting 18 min:54 s. (± 1 min).

#### Cognitive scores and tasks

The test battery consisted of a Psychomotor Vigilance test (PVT, 6 min ± 10 s), Word Memory Test (WMT, 2 min ± 42 s), forward memory digit span test (SPAN, 30 s ± 7 s), spatial N-Back (3-Back) (2 min:25 s ± 8 s) and multiple-choice tasks in language (2 min:30 s ± 8 s), logic (3 min:27 s ± 60 s) and numeric (2 min:40 s ± 47 s). The processing time of each task was recorded. Before beginning the study, each subject completed a training run of all tasks.

The PVT^28^ measuring and immediately indicating reaction time was carried out on a Pocket PC hp jornada 560 Microsoft. In a dark environment, subjects had to press a button upon the appearance of a green light LED. Trials lasting longer than 850ms were classified as lapses.

##### Memory tasks

Word Memory Test (WMT) and SPAN were programmed with Visual Basic Application 6.0 (VBA) in Microsoft Excel. The WMT, adapted from^29,30^ consisted of 22 pairs of German nouns (e.g., climate—storm). 4 additional dummy word pairs at the beginning and the end were used to avoid the primacy and recency effect. Each word pair was presented for 5 s. In the recall phase, subjects had to type the second word of the pair upon presentation of the first word without time pressure. SPAN contained 12 random single-digit numbers that were displayed for 5 s. Thereafter subjects were requested to type the number in the same order without time pressure. A total of 8 different lists for 8 sessions were prepared for both tasks. The order of the lists, word pairs, and numbers within each list was randomized for each subject. The spatial triple N-back (3-back) was provided with the Brain workshop software^31^ Version 4.8.4. Two consecutive tests were performed each lasting 72 s (24 trials each 3 s). In a sequence, squares appeared on a 3 × 3 matrix and the button had to be pressed if the actual square presentation matched the third before. Correct and wrong answers were counted.

##### Cognitive multiple-choice tests

Covered were the categories language (21 tasks), logic (17 tasks), and numeric (8 tasks), chosen from the IQ-Test training^32^. Language sub-categories comprised finding analogies (5 tasks), arranging 5 letters to one word (7 tasks), finding words with common generic terms (4 tasks) and those not matching to a list (5 tasks). Logical tasks were the completion of figure patterns (8 tasks), mental rotation such as rotating and flipping figures (4 tasks), mapping and folding figures (3 tasks), and turning and tilting of dice (2 tasks). Numeric tasks were completing number sequences by finding the pattern (4 tasks) and addition of numbers (4 tasks). A total of 8 different batteries were prepared for 8 runs and divided into series A and B (each containing 4 batteries) for the two measurement nights. The order of both series was randomized and balanced over subjects.

### MRI/MRS measurement

A 3.0 Tesla Magnetom Prisma Scanner (Siemens) was equipped with a double tuned ^31^P-^1^H head coil from Rapid biomedical (Würzburg, Germany). Subjects had to fix a spot with open eyes and were permanently monitored via mirrors, camera, and pulse oximeter. Anatomical reference images were acquired using T_1_-weighted 2D Flash sequences.

#### ^31^P and ^1^H MRS sequences

After shimming (5 min), two 2D chemical shift imaging (CSI) ^31^P-MRS sequences were applied to acquire axial grids of 8 × 8 voxels (25 mm)^3^ over 22 min each, first in a middle plane centered onto the thalamus, second above the corpus callosum (Fig. 1B). The first axial CSI slice was positioned in the isocenter of the scanner, with its row No. 5 centered mid-sagittally in the thalamus region and tilted parallel to the ac-pc plane. Manual shimming was performed to ensure FWHM ≤ 25 Hz. Parameters were: field of view (FOV) = 20 cm × 20 cm, echo time (TE) = 2.3 ms, repetition time (TR) = 3500 ms, averages = 43, acquisition time (TA) = 22.35 min, tip angle = 90°, complex points = 1.024 and bandwidth (BW) = 2 kHz. The second CSI grid was placed adjacent above the corpus callosum (Fig. 1B) using the same parameters and manual shimming. Each of the very silent CSI-sequences was applied over 20 min. Acquisition of the middle CSI-grid was started 20 min after stopping ad libitum activity and positioning in the scanner and only 5 min after ending the noise and vibrations of the preceding 2D flash sequence, respectively. The upper CSI-grid was acquired immediately after that.

Then, in one (50 × 25 × 25 mm^3^) frontal—and two (25 mm)^3^ medial parietal single voxels, ^1^H-spectroscopy was carried out (Fig. 1B) using a PRESS (WS) sequence with the parameters: repetition time (TR) = 3000 ms, echo time (TE) = 80.0 ms averages = 45, acquisition time (TA) = 2.21 min, tip angle = 90°, complex points = 2.048 and bandwidth (BW) = 2 kHz.

### MRS data analysis

In vivo as well as phantom DICOM ^31^P-MRS and ^1^H-MRS data were analyzed with TARQUIN 4.3.10^33^ resulting in single or combined peak integrals. All data underwent a Fourier transformation as well as zero and first order phase correction.

#### ^31^P-CSI

**Figure 2:**
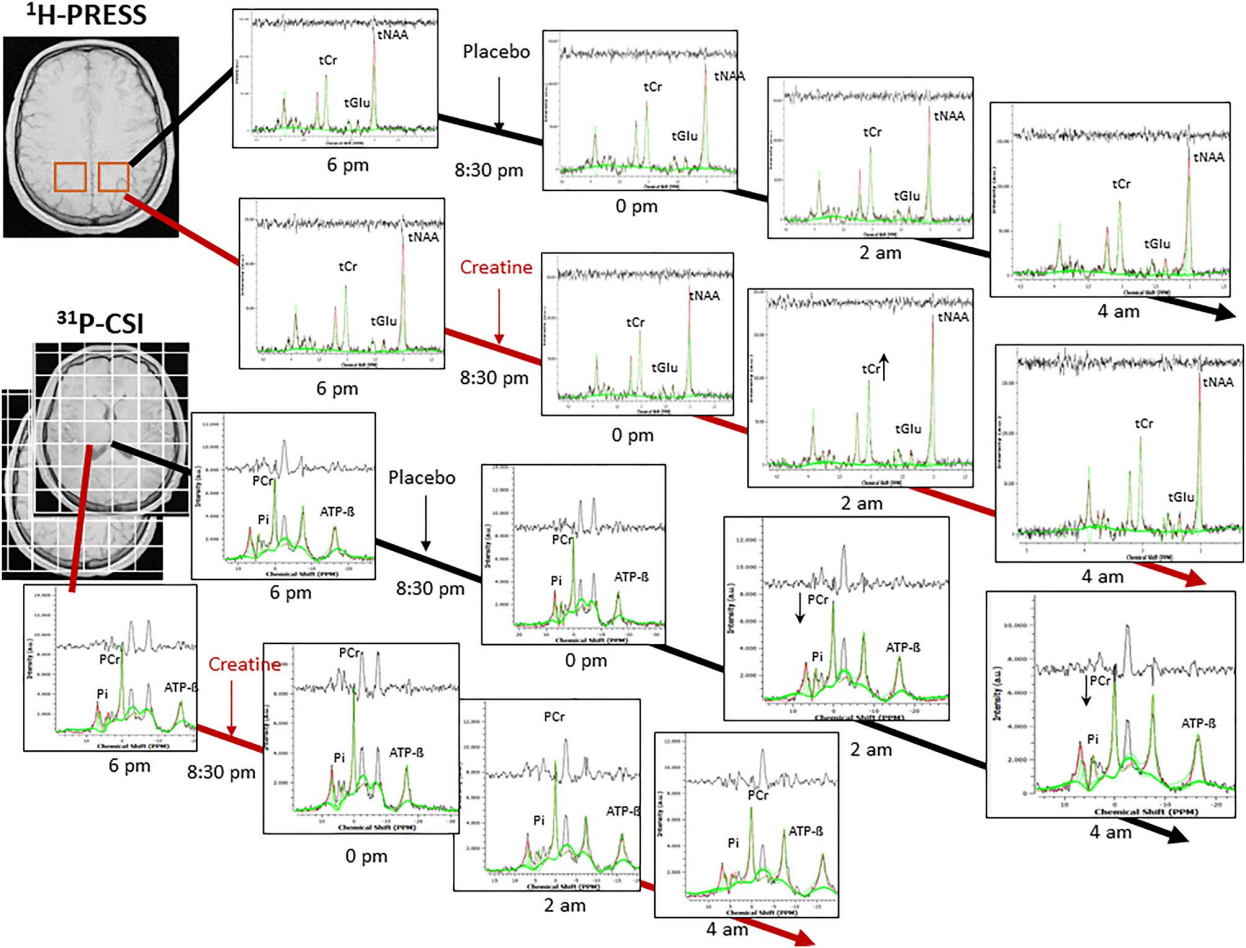
Plots of ^1^H and ^31^P spectra over time, including processed signal, fit (in green), baseline and residual of left parietal voxel (PRESS), and from one CSI voxel (R5C4) located in the middle grid.

From two 8 × 8 CSI grids, spectra of 2 × 9 voxels, each, were evaluated (listed in Tables  S2–S6, 2). Voxels not entirely containing brain tissue were excluded. All data were fitted as linear combinations of the simulated metabolic basis set including PCr, ATP-ß, Pi, PE, TCho, GPC, and GPE. While PCr, Pi, PE, GPC, and GPE appear as singlet Lorentzian peak, signals from the α- and γ phosphor atoms in ATP were modeled as doublet and ATP-ß as triplet accounting for the homonuclear j-coupling (16 Hz) with one or two neighbors respectively. Since the in vivo signals at the ATP-ß chemical shift position originate exclusively from ATP, only these were considered to evaluate ATP levels, referred to as NTP by others. As by convention, PCr was set at 0 ppm as spectral reference. In addition, all ^31^P spectra were ^1^H decoupled. The k-space filter required for CSI-MRS was switched on resulting in a voxel size of 25 mm^3^. Regarding fitting parameters, the initial ß value, which determines the mixing ratio of Lorentzian and Gaussian (Voigt) line shape, was chosen 600 Hz^2^ for the middle and 1000 Hz^2^ for the upper CSI slice. The max. metabolite—as well as the max. broad shift, both were set at 3.0 ppm. Regarding the post processing, a zero-filling factor of 2 and a line broadening of 10 Hz were used. Figures 1B, 2 shows an individual ^31^P spectrum of one CSI voxel covering the left thalamus (row 5, column 5) and one ^1^H spectrum, located in the left superior parietal region acquired at 6 p.m.

For this study, the ratio to total phosphorus signal of the Tarquin reliable data, including the sum of the signal integral of PCr, ATP-ß, Pi, PE, and TCho (declared ^31^P) was chosen to assess changes within each metabolite.

#### ^1^H-PRESS

Voxel signals were fitted as linear combinations of the simulated basis set including tCr, tNAA, Cho, Glx and GABA for ^1^H-MRS. Pre water shift and lipid filter were switched on and fit parameter were chosen: ß = 200 Hz^2^, water attenuation = 0.7, metabolite shift = 1.0, broad shift = 1.0 ppm. Regarding the post processing, a zero-filling factor of 2 and a line broadening of 5 Hz were used.

#### pH calculations

The pH values were calculated from the chemical shift of Pi, according to the derivation of the Henderson-Hasselbalch equation^34^

1 $$pH=6.66+{\text{log}}\frac{\delta pi-3.08}{5.57-\delta pi}$$

Before pH means were calculated, the logarithmic pH values were transformed to linear [H_3_O] concentrations and thereafter back-transformed to the pH scale.

### Statistics and evaluation

Since the study was longitudinal, the within subject version of the two tailed T-test was applicable for the comparison of means in results of MRS and cognitive performances. For the intraindividual comparison, a chosen statistical power of 0.90 and effect size of 1.33 (mean difference = 0.04, pooled standard deviation = 0.025) required a minimum sample size of n = 12 calculated by statistical power tool G*Power (Version 3.1.9.7, HHU-Düsseldorf).

Changes versus baseline in cognitive and metabolic parameters in each voxel were calculated by the mean difference between BL (6 p.m.) and another session (0 a.m., 2 a.m. or 4 a.m.). Regarding metabolic changes, SD-related variations in the spectral quality between time points were included as a weighting factor to allow a more robust and precise analysis (Supp. 2.5). Changes versus placebo were calculated as the difference between the changes (${\Delta }_{iCr}$-${\Delta }_{iPl}$) in the creatine and placebo condition. Due to the limited sample size, possible false Type error II results have to be considered. Significance thresholds were adjusted for multiple testing according to the Bonferroni approach. As measurements were carried out at eight conditions (*k* = 8, 2 sessions each 4 runs), the α thresholds were adjusted to α = 0.05/8 = 0.0063.

Correlations in changes of metabolic parameters and cognitive scores were analyzed by calculating Pearson correlation coefficient (r) (Supp. 2.5).

### Segmentation, reproducibility and calibration

The composition of gray matter (GM), white matter (WM), and cerebrospinal fluid CSF were segmented (Supp. 2.6, Table S8), and the contribution of signal changes for each voxel due to the spatial displacement between the time points was calculated (Supp. 2.7, 3.4, Tables S9–S12). Furthermore, subject variability across all regions, conditions, and baseline differences between both conditions were compared. Multiple phantom measurements on different nights were performed to calculate the signal drifts from the baseline of each time point (Supp. 3.5).

## Results

The Insomnia Severity Index (ISI) resulted in ISI = 4.6 ± 1.6 (ISI_max_ ≤ 6). The requested sleep time schedule revealed an average sleep/wake up time of 11:25 p.m. (± 33 min) and 7:12 a.m. (± 53 min) with an average sleep duration of 07 h:54 min (± 43 min) for subjects starting with placebo and 07 h:39 min (± 31min) for those with creatine. Differences in sleep duration between the first and the second session revealed a nonsignificant deviation of + 8 min (± 45 min, *p* = 0.62, *t* = 0.53) for the placebo and + 14 min (± 37 min, *p* = 0.34, *t* = 1.07) for the creatine group. All 15 subjects completed the study. The SD was effective, significantly impacting established parameters. Nobody fell asleep during scans and sessions. Creatine was well tolerated. No gastric discomfort or other physical complaint was signalized. Wakefulness was steadily confirmed by monitoring responsiveness.

**Figure 3:**
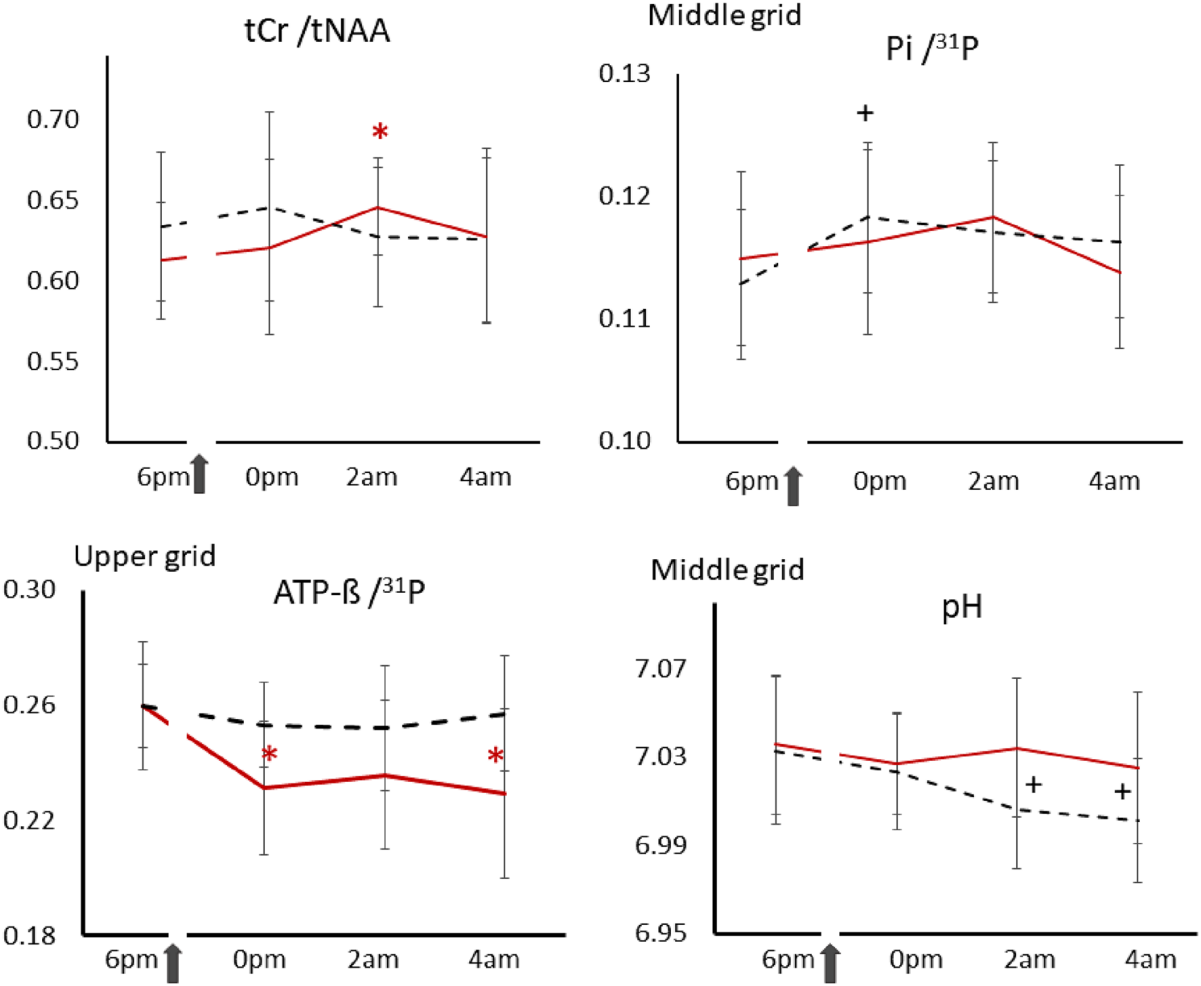
Time courses of selected metabolic parameters during sleep deprivation after oral administration of creatine (red solid lines) or placebo (black dashed lines). Shown are tCr/tNAA of the voxel located in the left medial parietal region, and averages of Pi/^31^P, ATP-ß/^31^P and pH levels of the middle and upper grid voxels. ^31^P represents the total phosphorus signal, including PCr, Pi, ATP-ß, PE, and TCho. Arrows indicate administration of creatine or placebo at 8:30 p.m. Asterisks (*) represent significant changes versus baseline after creatine, and (+) after placebo administration (*p* ≤ 0.005) that survived the Bonferroni correction. Bars denote standard errors (SE).

**Figure 4:**
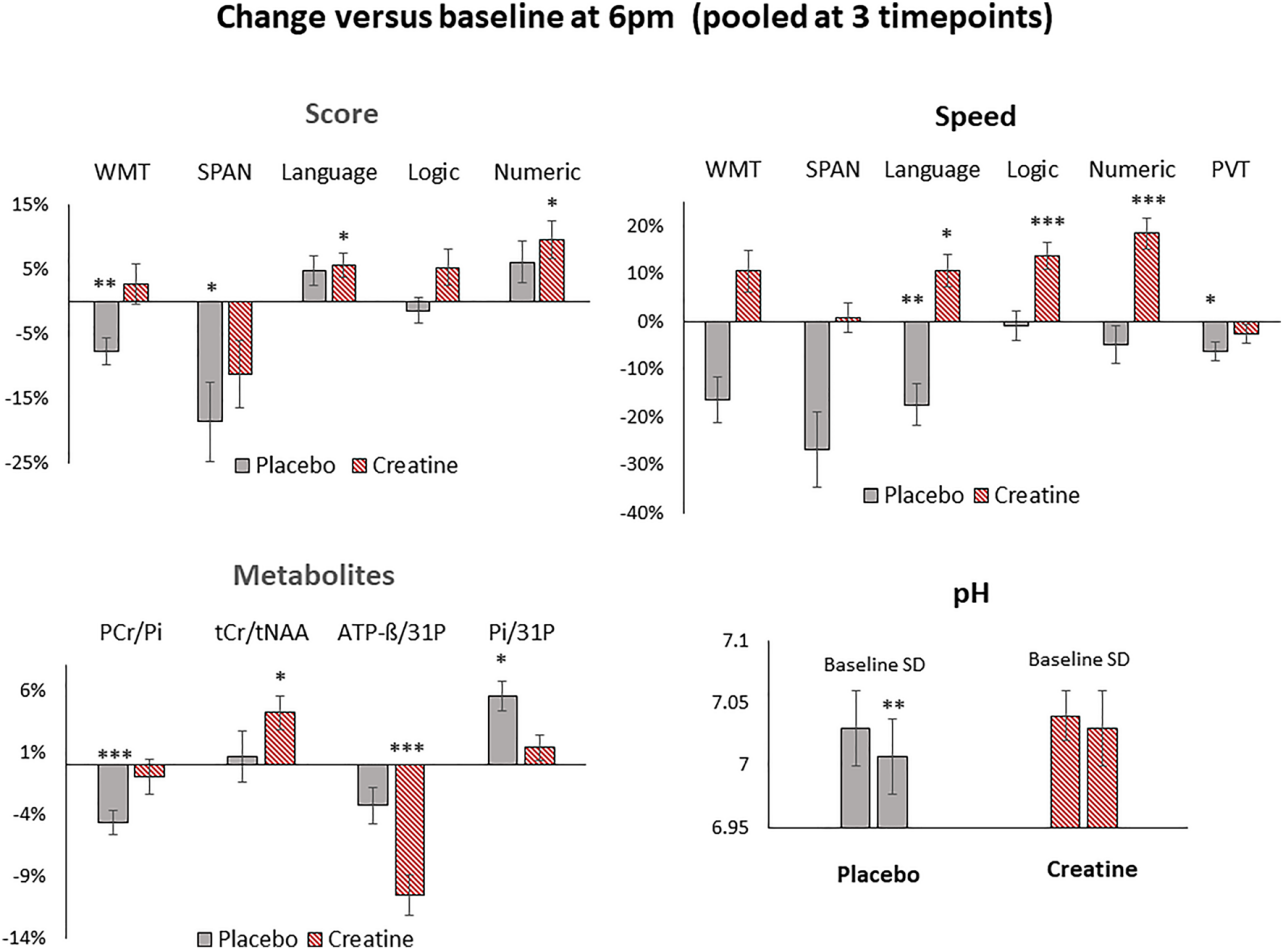
Changes in cognitive performance and metabolites versus baseline (6 p.m.) during sleep deprivation under placebo (grey) and creatine (red) when pooled at all 3 timepoints (0 p.m., 2 a.m., 4 a.m.). Shown are changes in cognitive tasks (Language, Logic, Numeric), forward digit span (SPAN), word memory tasks (WMT), psychomotor vigilance test (PVT, reaction speed) and selected metabolic parameters of tCr/tNAA from the left medial parietal region, of PCr/Pi, Pi/^31^P and pH level from the averaged middle.-, and ATP-ß/^31^P from the averaged upper grid. Significance levels are presented by *p_43_ < 0.005, **p_43_ < 0.0005 and ***p_43_ < 0.00005. Bars represent standard errors (SE).

**Figure 5:**
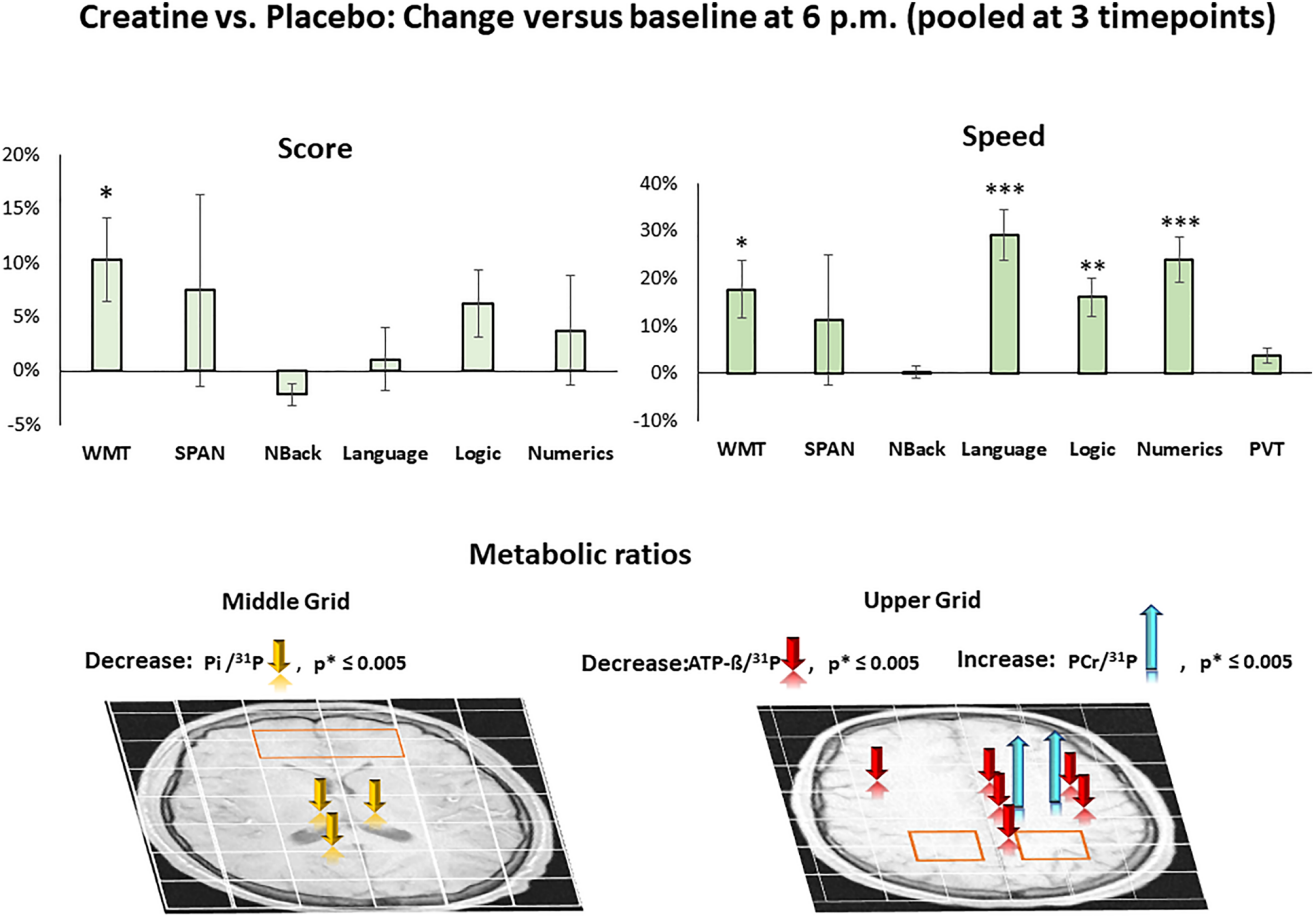
Baseline (6 p.m.) related changes in cognitive performance, speed in processing time and metabolic parameters after oral administration of creatine versus placebo when pooled at 3 points (0 p.m., 2 a.m., 4 a.m.). Creatine administration led to significant improvements in word memory task (WMT), speed in processing time in WMT, language, logic, and numeric tasks, and induced declines in ATP-ß/^31^P, Pi/^31^P, and increase in PCr/^31^P. Significance levels are color coded and indicated by arrows onto axial brain slices in radiological orientation.

Cognitive parameters and mean within changes versus baseline in both conditions are given in Tables 1, S1, Figs. 4, S3, S4. Baseline-related changes in cognitive parameters of creatine versus placebo are given in Figs. 5, S5 and are presented in “Cognitive and metabolic response of creatine versus placebo”. Ratios of MRS-signals and mean within changes versus baseline of PCr/^31^P, ATP-β/^31^P, Pi/^31^P, PCr/Pi, ATP-β/PCr, PE/^31^P, TCho/^31^P, tCr/tNAA, Glu/tNAA and pH level in both conditions are given in Tables S2–S5, Figs. 3, 4, S2–S4. Baseline-related changes in PCr/^31^P, ATP-β/^31^P, Pi/^31^P, PCr/Pi, ATP-β/PCr, tCr/tNAA, and Glu/tNAA levels of creatine versus placebo are given in Tables 2, S6, Figs. 5, S5. Correlations of cognitive and metabolic response are shown in Table S7a–d, Fig. S6. PANAS did not differ significantly between sessions, with positive and negative subscales amounting to 3.2 ± 0.1 and 1.2 ± 0.1 in the placebo and 3.1 ± 0.2 and 1.3 ± 0.1 in the creatine session. Only data that withstand the Bonferroni correction were presented.

**Table 1:** Outcome of cognitive tasks and scales. Mean number of correct results, processing time and intra-individual percentual changes versus baseline (6 p.m.) after creatine or placebo. Changes in Karolinska Sleepiness Scale (KSS) and Fatigue scale before (bef.) and after (aft.) each session are shown below. WMT, Word memory test. Data are given ± SD.

Task	No. of trials	Number of correct results (score)														
		6 p.m.		0 a.m.		2 a.m.		4 a.m.								
		Placebo	Creatine	Placebo	Creatine	Placebo	Creatine	Placebo	Creatine							
WMT	22	18.1 ± 3	16.7 ± 4.2	17.0 ± 3.0	17.4 ± 2.8	16.4 ± 3.4	18.1 ± 2.9	16.6 ± 4.0	16 ± 4							
vs. 6 p.m. (%)				− 5.9%	3.9%	− 9.1%^+^	8.2%	− 8.3%^+^	− 3.9%							
Digit span	12	8.9 ± 2.6	8.9 ± 2.2	8.0 ± 2.6	7.4 ± 2.1	6.7 ± 2.5	9.0 ± 2.2	7.1 ± 2.3	7.2 ± 2.7							
vs. 6 p.m. (%)				− 10.4%	− 16.9%	− 24.8%^+^	1.6%	− 20.8%^+^	− 18.5%							
N-back	13.8 (6–30)	^1^4.2/6.9	4.3/7.5	4.4/6.3	4/6.6	4.7/7.0	4.7/7.3	4.7/7.1	4.5/7.3							
vs. 6 p.m. (%)				12 ± 28%	9 ± 26%	4 ± 24%	14 ± 28%	13 ± 25%	10 ± 39%							
Language	21	16.6 ± 2	17.1 ± 1.7	17.3 ± 2.3	18.3 ± 1.9	17.6 ± 2.2	17.3 ± 1.7	17.3 ± 1.7	18.2 ± 1.8							
vs. 6 p.m. (%)				4.0%	7.8%^+^	6.0%	2.0%	4.0%	7.1%^+^							
Logic	17	14.5 ± 1	13.6 ± 2.5	14.4 ± 1.3	14.5 ± 1.7	14.9 ± 1.2	14.3 ± 1.5	13.7 ± 1.7	14.2 ± 1.2							
vs. 6 p.m. (%)				− 0.9%	6.4%	2.3%	4.9%	− 5.5%	4.4%							
Numeric	8	6.5 ± 1	6.7 ± 1.6	6.9 ± 1.1	7.4 ± 1.1	7.3 ± 1.2	7.7 ± 0.9	6.6 ± 1.7	7.1 ± 1.3							
vs. 6 p.m. (%)				6.1%	9.9%	11.2%	13.9%^+^	1.0%	5.0%							

Task	No. of trials	Processing time (t)														
		6 p.m.		0 a.m.		2 a.m.		4 a.m.								
		Placebo	Creatine	Placebo	Creatine	Placebo	Creatine	Placebo	Creatine							
WMT	22	101 ± 37 s	112 ± 42 s	107 ± 26 s	100 ± 25 s	116 ± 36 s	101 ± 27 s	98 ± 22 s	99 ± 42 s							
vs. 6 p.m. (%)				5.7%	− 10.3%	15.1%	− 10.8%	− 2.6%	− 12.9%							
Digit SPAN	12	26.8 ± 10 s	30.2 ± 8 s	31.2 ± 13 s	34.6 ± 20 s	27.3 ± 7 s	28.4 ± 10 s	30.2 ± 10 s	27.0 ± 15 s							
vs. 6 p.m. (%)				16.5%	14.3%	2.0%	− 6.0%	12.7%	− 10.6%							
Language	21	137 ± 35s	165 ± 32 s	161 ± 33 s	139 ± 33 s	162 ± 35 s	150 ± 31 s	159 ± 34 s	152 ± 31 s							
vs. 6 p.m. (%)				17.5%^+^	− 15.6%^+^	18.7%^+^	− 8.7%	16.0%^+^	− 7.5%							
Logic	17	214 ± 63 s	227 ± 60s	219 ± 54 s	198 ± 52 s	214 ± 54 s	193 ± 46 s	214 ± 52 s	196 ± 45							
vs. 6 p.m. (%)				2.4%	− 13%^+^	0.2%	− 15%^+^	0.2%	− 14%^+^							
Numeric	8	127 ± 37 s	147 ± 27 s	126 ± 34 s	111 ± 25 s	131 ± 28 s	121 ± 23 s	139 ± 38 s	128 ± 33 s							
vs. 6 p.m. (%)				− 0.4%	− 24% **	3.4%	− 18% ^+^	9.6%	− 13%^+^							

Karolinska Sleepiness Scale (KSS)^a^																
	6 p.m.				0 a.m.				2 a.m.				4 a.m.			
	Placebo		Creatine		Placebo		Creatine		Placebo		Creatine		Placebo		Creatine	
	bef.	aft.	bef.	aft.	bef.	aft.	bef.	aft.	bef.	aft.	bef.	aft.	bef.	aft.	bef.	aft.
Score	2.7	2.7	3.1	3.1	6.1	5.9	5.8	5.8	6.8	6.8	7.1	6.9	7.4	7.4	7.8	7.5
aft vs. bef	0		0.07		− 0.2		0		0		0.28		− 0.1		0.33*	
vs. 6 p.m. (%)					122%***		92%***		150%***		128%***		172%***		145%***	

Inverted Samn & Perelli Fatigue score (FAT)^b^																
	6 p.m.				11 p.m.				2 a.m.				4 a.m.			
	Placebo		Creatine		Placebo		Creatine		Placebo		Creatine		Placebo		Creatine	
	bef.	aft.	bef.	aft.	bef.	aft.	bef.	aft.	bef.	aft.	bef.	aft.	bef.	aft.	bef.	aft.
Score	6.4	6.6	6.5	6.6	10.7	10.5	10.9	10.7	13.8	14.1	13.2	12.4	15.7	15.6	14.9	14.2
aft vs. bef	0.1		0.1		− 0.2		− 0.1		0.4		− 0.8		− 0.1		− 0.6	
vs. 6 p.m. (%)					63%**		65%**		115%***		96%**		141%***		122%**	

^a^Correct/total trials; ^b^scales acquired before and after passing the battery of 7 tests *p = values of *p* ≤ 0.005, **p = values of *p* ≤ 0.0005, *** p = values of *p* ≤ 0.00005,^+^p = values of 0.0063 ≤ *p* ≤ 0.05, that did not survive Bonferroni correction.

**Table 2:** Mean within-subject response to creatine versus placebo: Changes versus baseline (6 p.m) of PCr/Pi, ATP− ß/PCr, and PCr, ATP− ß to total phosphorus signal ^31^P (including PCr, Pi. ATP-ß, PE and Tcho) in middle and upper ^31^P-CSI-slice, and of tCr/NAA and Glu/tNAA in single voxels (1H-SVS) at 0 a.m., 2 a.m. and 4 a.m.

Middle slice			Creatine versus placebo						Creatine versus placebo					
Voxel no	Hs	Anatomical label	0 p.m. vs. 6 p.m.		2 a.m. vs. 6 p.m.		4 a.m. vs. 6 p.m.		0 p.m. vs. 6 p.m.		2 a.m. vs. 6 p.m.		4 a.m. vs. 6 p.m.	
			PCr/^31^P	PCr/Pi	PCr/^31^P	PCr/ Pi	PCr/^31^P	PCr/Pi	ATP-ß/ ^31^P	ATP-ß/PCr	ATP-ß/ ^31^P	ATP-ß/PCr	ATP-ß/ ^31^P	ATP-ß/PCr
R4C3	r	Anterior insula	3%	1%	2%	0%	4%	2%	0%	− 5%	− 1%	− 6%	− 1%	− 9%
R4C6	l		− 4%	1%	3%	0%	0%	3%	3%	9%	− 3%	− 10%	1%	2%
R5C3	r	Temporal transversal	− 1%	0%	0%	6%	5%^+^	9%	2%	5%	4%	8%	1%	− 2%
R5C6	l		2%	10%	1%	0%	1%	3%	1%	− 2%	− 1%	− 6%	0%	− 2%
R6C3	r	Temporal medulla	− 3%	− 3%	2%	4%	3%	2%	3%	7%	1%	− 1%	2%	− 1%
R6C6	l		0%	7%	1%	1%	− 2%	1%	1%	0%	− 4%	− 11%	1%	2%
R7C3	r	Occipito− temporal	− 7%	− 16%	− 2%	− 5%	0%	4%	3%	14%^+^	1%	4%	3%	5%
R7C6	l		− 6%	− 1%	− 4%	− 4%	− 4%	1%	1%	1%	− 5%^+^	− 10%	− 1%	− 2%
R3C4	r	Anterior cingulum	− 4%	− 4%	0%	− 3%	2%	5%	4%^+^	14%*	− 3%	− 4%	1%	− 1%
R3C5	l		− 1%	0%	0%	− 3%	3%	2%	5%	9%	4%	5%	5%	4%
R4C4	r	Capsulo− striatal	0%	3%	0%	1%	0%	5%	3%	4%	3%	4%	3%	6%
R4C5	l		− 1%	6%	0%	− 1%	− 2%	5%	2%	8%	1%	5%	3%	12%
R5C4	r	Thalamo− capsular	− 2%	7%	0%	3%	1%	8%	4%^+^	9%^+^	2%	5%	2%	2%
R5C5	l		2%	10%^+^	4%	7%	1%	11%^+^	1%	0%	− 3%	− 11%	2%	2%
R6C4	r	Corpus callosum	− 2%	4%	0%	5%	1%	8%^+^	2%	4%	1%	− 1%	2%	− 1%
R6C5	l		1%	6%	4%	6%	2%	7%	0%	− 1%	− 4%	− 12%	0%	− 1%
R7C4	r	Occipito− medial	− 6%	− 8%	− 1%	0%	− 1%	3%	3%	12%	1%	5%	3%	6%
R7C5	l		− 3%	1%	− 1%	3%	− 2%	1%	− 1%	− 2%	− 4%	− 9%	− 1%	− 2%

Upper slice			0 p.m. vs. 6 p.m.		2 a.m. vs. 6 p.m.		4 a.m. vs. 6 p.m.		0 p.m. vs. 6 p.m.		2 a.m. vs. 6 p.m.		4 a.m. vs. 6 p.m.	
			PCr/^31^P	PCr/ Pi	PCr/ ^31^P	PCr/ Pi	PCr/^31^P	PCr/ Pi	ATP-ß/^31^P	ATP-ß/PCr	ATP-ß/^31^P	ATP-ß/PCr	ATP-ß/ ^31^P	ATP-ß/PCr
R4C3	r	Lateral premotor	2%	− 2%	− 5%	− 2%	− 4%	− 6%	− 6%	− 8%	− 9%	− 17%	− 10%^+^	− 11%
R4C6	l		− 2%	− 11%	1%	− 15%	0%	− 2%	− 13%^+^	− 25%^+^	− 13%^+^	− 29%^+^	− 12%^+^	− 28%^+^
R5C3	r	Motor	7%^+^	− 6%	− 2%	− 4%	7%^+^	− 10%	− 10%^+^	− 13%	− 6%	− 7%	− 4%	− 5%
R5C6	l		2%	− 6%	1%	− 1%	1%	5%	− 11%^+^	− 21%^+^	− 8%^+^	− 15%^+^	− 7%	− 20%^+^
R6C3	r	Ant. later. Parietal	2%	− 3%	− 1%	0%	8%^+^	5%	− 7%	− 9%	1%	4%	− 3%	− 6%
R6C6	l		0%	3%	3%	2%	2%	10%^+^	− 5%	− 9%^+^	− 8%^+^	− 16%^+^	− 6%	− 14%
R7C3	r	Post. lateral parietal	− 2%	0%	0%	3%	6%	16%^+^	− 8%	− 11%	0%	5%	− 7%	− 16%
R7C6	l		− 3%	8%	− 3%	− 3%	1%	2%	− 13%	− 16%	− 11%	− 12%	− 15%^+^	− 24%
R3C4	r	Anterior F1	− 8%	− 6%	− 7%	− 8%	− 8%	− 6%	4%	14%	− 7%	− 8%	− 1.3%	8%
R3C5	l		1%	− 3%	− 5%	− 8%	− 2%	1%	0%	4%	− 16%^+^	− 26%	− 8%	− 7%
R4C4	r	Posterior F1	0%	− 6%	− 3%	− 6%	− 4%	− 5%	− 7%	− 10%	− 6%	− 8%	− 6%	− 10%
R4C5	l		2%	− 4%	− 3%	− 8%	− 3%	1%	− 8%^+^	− 14%^+^	− 8%	− 14%	− 6%^+^	− 15%^+^
R5C4	r	Medial premotor	2%	− 3%	0%	2%	4%	4%	− 7%^+^	− 11%	− 4%	− 7%	− 8%	− 15%^+^
R5C5	l		3%	− 3%	4%	− 1%	3%	4%	− 8%*	− 14%^+^	− 7%^+^	− 14%^+^	− 8%^+^	− 20%*
R6C4	r	Medial central	4%	− 6%	2%	1%	6%	9%	− 8%^+^	− 11%	− 2%	− 5%	− 7%^+^	− 13%
R6C5	l		3%	− 5%	2%	− 4%	6%	5%	− 9%^+^	− 11%	− 6%	− 9%	− 12%^+^	− 23%^+^
R7C4	r	Precuneus	− 1%	− 6%	− 1%	− 10%	7%	7%	− 8%	− 10%	− 1%	3%	− 10%^+^	− 15%
R7C5	l		− 2%	− 6%	− 2%	− 9%	4%	6%	− 8%	− 9%	− 4%	− 1%	− 11%^+^	− 22%

Δ tCr/tNAA					Δ Glu/tNAA									
Anatomical label		0 a.m. vs. 6 p.m.	2 a.m. vs. 6 p.m.	4 a.m. vs. 6 p.m.	0 a.m. vs. 6 p.m.	2 a.m. vs. 6 p.m.	4 a.m. vs. 6 p.m.							
r	Med. post. parietal	0.3%	− 3.0%	6.1%	6.1%	− 3.9%	4.9%							
l		2.4%	6.2%^+^	4.8%	17.3%	− 11.1%	− 25.4%							
Frontal		^1^15.8%	^2^4.0% 8%	^3^– 6.5% − 8.4%										

^1,2,3^Data from only ^1^6 subjects or ^2,3^7 subjects were available, *p = values of *p* ≤ 0.0064, that survived Bonferroni correction, ^+^p = values of 0.0063 ≤ *p* ≤ 0.05, that did not survive Bonferroni correction.

### Cognitive and metabolic response to SD

Under placebo KSS and FAT progressively increased in all subjects except FAT in one, terminally at 4 a.m. by 172 ± 17% and 141 ± 23%, significant at each versus every preceding time point (*p*_12_ < 0.0002, *t*_12_ > 3.2, vs. baseline and all other comparisons; Table 1).

Furthermore, deteriorations in memory tasks versus baseline appeared significant in WMT (− 7.8 ± 2.1%, *p*_43_ = 0.0002, *t*_43_ = − 3.8); SPAN (− 18.7 ± 6.1%, *p*_43_ = 0.002, *t*_43_ = − 3.0) and in speed in processing time in the language task (− 17.4 ± 4.4%, *p*_43_ = 0.0001, *t*_43_ = − 4.0) and in PVT (0.9-Pc) (− 6.2 ± 1.9%, *p*_43_ = 0.001, *t*_43_ = − 3.3) when pooled at all 3 time points (Fig. 4).

Significant increase Pi/^31^P versus baseline revealed regional and in the averaged middle grid at 0 p.m. (5.5 ± 1.2%, *p*_13_ < 0.001, *t*_13_ = 4.39) (Table S3, Figs. S3, 3). Decrease in PCr/Pi revealed regional at all timepoints (0 p.m., 2 a.m., 4 a.m.) and in the averaged middle grid (− 4.7 ± 1.0%, *p*_43_ < 0.00005, *t*_43_ = − 4.53) when pooled at all 3 time points (Table S3, Fig. 4). pH level of 7.03 (95% Cl 7.00 − 7.07) dropped here significantly by 0.026 ± 0.03, *p*_13_ = 0.004, *t*_13_ = − 3.43 (95% Cl 7.03 − 6.98) at 2 a.m. and 0.032 ± 0.029, *p*_13_ = 0.001, *t*_13_ = − 4.19 (95% Cl 7.03 − 6.97) at 4 a.m. (Table S4, Figs. 3, 4).

Positive correlations was found in the right hemisphere between cognitive performance (SPAN, PVT, numeric and logic task) and metabolic response in PCr/Pi and ATP-ß/^31^P shown in Table S7a,b, Fig. S6.

### Cognitive and metabolic response to SD after creatine administration

Under Creatine KSS and FAT progressively increased terminally at 4 a.m. by 145 ± 9.9% and 122 ± 25%, significant at each versus every preceding time point (*p*_12_ < 0.009 vs. baseline and all other comparisons, *t*_12_ > 3.1, Table 1). Significant improvements versus baseline revealed in  processing time in numeric task at 0 p.m. (24.5 ± 4.3%, *t*_13_ = 5.0, *p*_13_ = 0.0003) (Table 1, Fig. S4) . Further improvements revealed in the language (5.6 ± 2.0%, *t*_43_ = 2.9, *p*_43_ = 0.003) and numeric (9.6 ± 3.2%, *t*_43_ = 3.2, *p*_43_ = 0.001) task and in processing time in language (10.6 ± 1.7%, *t*_43_ = 3.1, *p*_43_ = 0.002), logic (13.7 ± 2.8%, *t*_43_ = 4.8, *p*_43_ = 0.00001) and numeric (18.4 ± 3.0%, *t*_43_ = 5.9, *p*_43_ = 2.8 × 10^–7^) task when pooled at all 3 time points (Fig. 4).

Supplemented creatine yielded significant increase of cerebral tCr/tNAA versus baseline in the left medial parietal region at 2 a.m. (5.3 ± 4.1%, *t*_12_ = 4.1, *p*_12_ = 0.003) and when pooled at all 3 time points (4.2 ± 1.4%, t_42_ = 2.9, *p*_42_ = 0.006) (Table S3, Fig. 3, 4). Due to the challenging shimming in the frontal lobe region, ^1^H spectra fit values from only 6 subjects were available.

Global and regional extensive and progressive change over time revealed in the ATP-ß involved metabolic parameters (Tables S2, S4, Figs. 3, 4, S4). Significant declines occurred in the averaged upper grid in ATP-ß/^31^P (− 10.9 ± 2.6%, *p*_13_ = 0.002 at 0 p.m., − 12.8 ± 3.0%, *p*_13_ = 0.002, *t*_13_ = − 3.87 at 4 a.m.) and ATP-ß/PCr (− 12.4 ± 3.1%, *p*_13_ = 0.002, *t*_13_ = − 3.89 at 0 p.m., − 14.9 ± 3.0%, *p*_13_ = 0.001, *t*_13_ = − 4.45 at 4 a.m.) (Fig. 3).

Positive correlations were found between cognitive performance (WMT, SPAN, PVT and numeric) and response in HEP. ATP-ß/^31^P also revealed a negative correlation between logic task (Table S7c,d, Fig. S6).

### Cognitive and metabolic response of creatine versus placebo

Creatine significantly reduced fatigue in terms of FAT by 8 ± 7% (Wilcoxon’s *Z*_26_ = − 2.84, *p* = 0.002) at 2 a.m. and 4 a.m. pooled.

Significant improvements revealed in WMT (10.3 ± 3.8%, *t*_43_ = 2.7, *p*_43_ = 0.005) and speed in processing time in WMT (17.7 ± 6.0%, *t*_43_ = 3.0, *p*_43_ = 0.002), language (29.1 ± 5.3%, *t*_43_ = 5.3, *p*_43_ = 2.0 × 10^–6^), logic (16.0 ± 4.0%, *t*_43_ = 3.9, *p*_43_ = 0.0002) and numeric (24.0 ± 4.9%, *t*_43_ = 4.8, *p*_43_ = 1.02 × 10^–5^) task when pooled at all 3 time points (Fig. 5).

Creatine prevented the SD-induced changes in PCr/Pi and pH level. The prevention effect was regionally most pronounced in Pi/^31^P by a decline versus placebo in capsulo-thalamic (right − 8.5 ± 2.8%, *t*_43_ = − 3.63, *p*_43_ = 0.001; left − 9.6 ± 2.8%, *t*_43_ = − 2.34, *p*_43_ = 0.001), right corpus callosum (− 8.3 ± 2.6%, *t*_43_ = − 3.26, *p*_43_ = 0.002) and increase in PCr/^31^P in the left medial motor (4.2 ± 1.1%, *t*_43_ = 3.87, *p*_43_ = 0.0005) and left motor (5.7 ± 1.3%, *t*_43_ = 4.44, *p*_43_ = 0.0001) region when pooled at all 3 time points (Fig. 5).

Global and regional extensive and progressive change over time revealed in the ATP-ß involved metabolic parameters (Fig. 5, Table 2). Significant declines were reached in the upper grid when pooled at all 3 time points (ATP-ß/^31^P: − 8.5 ± 2.8%, *p*_43_ = 0.004, *t* = − 3.00; ATP-ß/PCr: − 11.3 ± 3.9%, *p*_43_ = 0.006, *t* = − 2.88). The declines in ATP-ß/^31^P were regionally most pronounced in the right lateral premotor (ATP-ß/^31^P: − 15.6 ± 4.9%, *t*_43_ = − 3.21, *p*_43_ = 0.003; ATP-ß), left posterior F1 (ATP-ß/^31^P: − 13.2 ± 4.0%, *t*_13_ = − 3.32, *p*_43_ = 0.001), left medial central (ATP-ß/^31^P: − 11.5 ± 3.5%, *t*_43_ = − 3.29, *p*_43_ = 0.002), left lateral premotor (ATP-ß/^31^P: − 18.4 ± 6.2%, *t*_13_ = − 4.23, *p*_43_ = 0.0001), left motor (ATP-ß/^31^P: − 18.2 ± 4.4%, *t*_43_ = − 4.12, *p*_43_ = 0.0002) and left medial premotor (left: ATP-ß/^31^P − 14.0 ± 3.0%, *t*_43_ = − 4.67, *p*_43_ = 0.00003) region. Declines in ATP-ß/PCr occurred in the same extent and degree and are shown in (Table S4, Fig. 5).

Positive correlation revealed between improvements in numeric tasks and regional increase in PCr/Pi while in turn improvemnts in PVT were associated with regional decreases in ATP-ß (Fig. S6).

## Discussion

In this randomized, controlled, double-blinded cross-over trial, we studied the response of cerebral PCr, ATP, Pi, tCr, Glu levels, and cognitive performance (i) to partial sleep deprivation (SD) versus baseline and (ii) to a single high dose of creatine versus baseline and placebo during SD. The SD led to a profound cognitive and metabolic response. Acute creatine was bio-available to the brain as suggested by increased tCr/tNAA and reduced subjective fatigue compared to the placebo condition. Creatine alleviated changes in phosphates, pH levels and fading of cognitive performance evoked by SD. Creatine induced increases in PCr/Pi, declines in ATP, and improvements in cognitive perfomance and processing speed exceeding wake baseline.

### Origin of MRS signals and neurochemical aspects

The measured signals in our study originate from metabolites involved in CK-BB in the cytosol. Due to the low viscosity, the resulting high mobility, and associated short correlation time of PCr, ATP, Cr, and Pi, most observed ^31^P-MRS and ^1^H signals are restricted to this location. With this, the total creatine tCr, as measured by ^1^H-MRS can be considered the sum of Cr and PCr signals. In energy demand, ATPase induces the exergonic breakdown of ATP to ADP and inorganic phosphate (Pi). The ATP deficit, in turn, is mainly compensated by the ADP-to-ATP conversion via CK-BB. In this process, the phosphate donor PCr brakes down to Cr, leading to a transient decline of PCr.

2 $${\text{PCr}} + {\text{ADP}} + {\text{H}}^{ + } \underset{{{\text{CK-MT}}1{\text{a}}}}{\overset{{\text{CK-BB}}}{\rightleftharpoons}}{\text{Cr}} + {\text{ATP}}$$

Hence, a temporary decrease of PCr associated with an increase in inorganic phosphate (Pi) is a well-established indicator of enhanced cellular energy consumption.

### Cognitive and metabolic response to sleep deprivation

The partial SD of this study significantly fatigued the subjects (by a factor of 3.2 in terms of FAT and KSS), effectively compromised the subject’s vigilance (PVT), and deteriorated short-term memory performance in digit SPAN and Word Memory Test (WMT). These are tasks known to be sensitive to sleep deprivation.

The observed decreases in PCr/Pi and pH levels during sleep deprivation in our study agree with outcomes previously reported by others^10,12,13,15^. The quiet wake state supine in the scanner during ^31^P-MRS did not bring PCr/Pi back to baseline as did a longer period of rest or recovery sleep^10,35^. Apparently, in contrast to muscular tissue, a longer time and deeper rest are required in brain tissue to regain baseline levels, respectively.

The changes in PCr/Pi in the right hemisphere showed a performance-related dependency between higher levels of PCr/Pi in the temporal region, and scores in PVT (0.1 percentile) and SPAN task (Fig. S6). SD and alertness-related studies showed a right hemisphere dependency in neuronal activation in the right hemisphere^36–40^.

These results along partial SD of 21 h considerably add to existing knowledge since ^31^P-MRS in humans had only been acquired after 36 and 40 h of SD^12,13^. Data on shorter SD only relay on ex vivo studies in animals ^35^.

### Cognitive and metabolic response to creatine during SD

Cognitive and metabolic effects of creatine could be observed starting from the first time point 3.5 h after creatine administration, well after reaching the reported plateau of serum levels^22^.The observed effects lasted or were augmented until the last measurement run ending 9 h after administration.

Creatine alleviated SD-induced fatigue and improved short-term memory tasks (WMT, SPAN) and reaction speed in PVT after oral creatine administration. In cognitive tests, processing time appears to be improved particularly in numeric and language performance 4 h after creatine administration. Improvements in complex deductive and executive tasks, memory tasks such as digit span, and reaction speed after SD are well-established effects of sub-chronical creatine^2,4,19^.

The high single dose of creatine in the oral formulation chosen was readily bioavailable to the brain, as suggested by increased tCr/tNAA in the left medial parietal region until 5.5 h after ingestion.

The observation was restricted to this region due to the limited voxel selection and thus could also occur elsewhere. However, a boosting effect here may have been caused by the higher demand due to the cognitive tests. This is supported by a positive correlation between a higher PCr/Pi level in the left antero.-lateral and posterior lateral parietal region and SPAN score at 2 a.m.

In the global average, except for an increase in some regions, PCr was similar to the placebo condition, while Pi and ATP showed significant differences versus placebo throughout the 3 time points assessed. Sleep deprivation-induced decreases in PCr/Pi were absent or alleviated in most regions. At the last time point, regional enhancements of PCr versus placebo were observed, amounting to up to 7% in the medial central and medial premotor region suggesting a progression of accumulation over time.

Creatine-induced decreases of Pi and ATP at relatively constant PCr are also well established for muscular tissue. 5 g creatine/day for 9 days at resting state reduced ATP by 9.1% and increased PCr by 6.6% at constant Pi, while under mild exercise (32% maximal), Pi was decreased by 32% and increased by 22% under intense exercise (79% maximal) versus un-supplemented baseline^41^.

The responses of PCr/Pi and ATP-β to creatine and SD differed on the average of the upper and middle CSI-grid, respectively. As this study was double-blinded and placebo-controlled, technical artifacts can be ruled out as a cause. We conclude that the differential responses of ^31^P-signals in the upper and middle grid reflect different biological states of the probed brains.

As with the placebo, the SD-sensitive tasks (WMT, SPAN, and PVT) also showed a performance dependency with higher levels of PCr/Pi after creatine administration. This time, it shows a positive correlation between the PCr/Pi levels in the left parietal region and SPAN. A negative correlation of ATP-ß/^31^P in PVT versus placebo indicates a higher high-boosting effect in this region.

All outcomes above indicate an increased creatine uptake^16–18^. In an equilibrium process, externally added creatine will shift the equilibrium of creatine kinases (Eq. 2) toward less ATP^18^. The equation of the law of mass action illustrates the quantitative relations:

3 $$K=\left(\frac{PCr}{ATP}\right)*\left(\frac{{ADP}^{*}H}{free Cr}\right)$$

**Figure 6:**
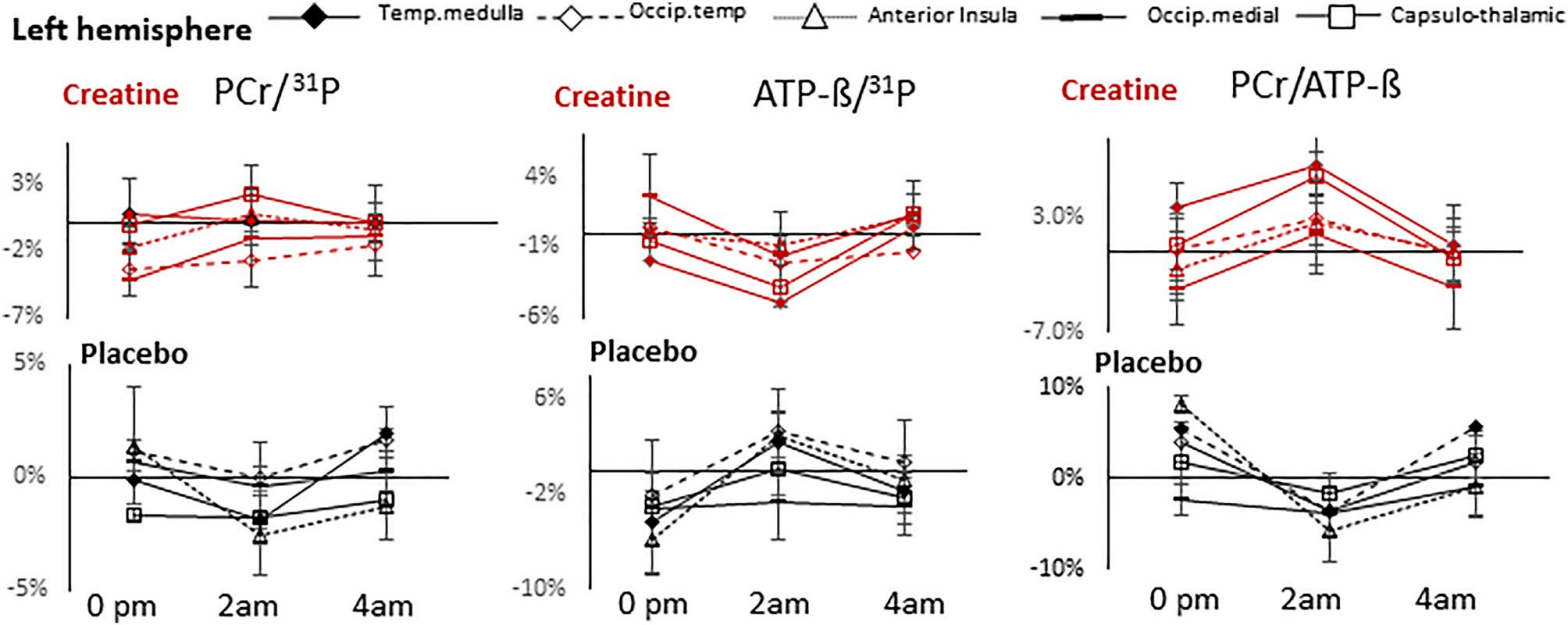
Time course of percentual changes from baseline of PCr/^31^P, ATP-ß/^31^P and PCr/ATP in selected voxels from left hemisphere at 0 a.m., 2 a.m. and 4 a.m. versus baseline 6 p.m. after creatine (red lines) and placebo (black lines).

Increasing total creatine will enable more transport of available ATP to sites of consumption. Regions close to equilibrium will yield increases upon external addition of free Cr, while regions not at equilibrium due to comparatively high energy demand might show decreases of PCr/ATP throughout the time points assessed. In line with^18^ was the observation of increasing ATP-ß in regions with low ATP-ß/PCr and a decrease in regions with high ATP-ß/PCr baseline levels. This becomes evident in (Fig. 6) by following the inverse course of ATP-ß and PCr/ATP after creatine throughout the creatine session (red lines in ATP-ß vs. PCr/ATP), as well as comparing placebo versus creatine-associated changes in ATP-ß and PCr/ATP separately (black vs. red lines in ATP-ß, black vs. red lines in PCr/ATP). Based on the mechanism above, creatine appears to compensate for reduced ATP-ß. Since the changes in ATP-ß are higher than in PCr and tCr, it cannot be ruled out that the circadian effect contributes as an additional factor to the amount of creatine and leads to a more substantial ATP decrease.

In contrast to placebo, creatine causes a substantial decrease in ATP-ß in the left relative to the right hemisphere at 2 a.m., indicating increasing energy demand in this hemisphere during the session.

Further evidence for the increased Cr uptake is the observed stabilization or decrease of Pi and a prevented drop of the pH level during SD. Increased Pi, such as in the case of increased cellular energy consumption, is a robust indicator of increased acidity in the cytosol. An effect of creatine preventing acidification was reported by Rico-Sanz et al.^41^.

The increase in creatine uptake in such a short period of time is remarkable. Given the absence of SLC6A8 in astrocytic feet’s surrounding the blood–brain-barrier (BBB) and the resulting very limited import from the periphery, creatine in CNS is mainly ensured by the endogenous synthesis driven by AGAT and GAMT^42,43^. This explains why CNS creatine replenishment takes a long time in patients with AGAT or GAMT deficiency treated with creatine. Hence, the brain is resistant and less reliant on exogenous creatine ingestion.

In view of these results, the question arises of what mechanism caused the increase in intracellular creatine uptake in our study. We suggest the SD condition combined with cognitive activity as a crucial factor triggering multiple mechanisms. SD has been reported to increase cerebral ammonia levels^44^, induce abnormalities in adenosine and metabolic response in Hyperammonemia^45^, and lead to altered expression and activity changes of genes involved in AGAT, GAMT and SLC6A8^42^. An excess of ammonia, such as in hyperammonemia, induces an increase in creatine transport^46^ and activates the SLC6A8 expression in astrocytes, leading to an increased Cr uptake in microcapillary endothelial cells (MCEC)^42^. The SD in our study would induce an increased SLC6A8 expression in astrocytes, thus preparing a condition for an increased Cr uptake to prevent ammonia toxicity, provided there is a high extracellular availability. This is ensured by the administration of a high dose creatine. The property of protecting the brain from excess ammonium is why creatine has been proposed as a suitable candidate for treating hyperammonemia patients to protect their developing CNS^20^.

A further explanation for the increased Cr uptake concerns the cerebral intra—or extracellular activity. Changes in intra—or extracellular creatine concentrations were investigated by Loike et al., Perasso et al.^23,47^. The passage of creatine against the concentration gradient between plasma and brain cells is assured by active transport via the Na^+^—and insulin-dependent creatine transporter creaT (SLC6A8)^48^. Hence, it can be assumed that creatine uptake is more effective at higher transmembrane Na^+^/K^+^ gradient and higher insulin. We suggest that increased acidification triggers higher activity in sodium hydrogen antiporter protein (NHE and NHE-1), which has the main aim to transport the excess protons into the extracellular space^49–52^. This mechanism, in turn, will increase the ATPase-related Na^+^-K^+^ electrochemical gradient, a crucial condition for an increased Na^+^ dependent creatine influx. According to Baldini et al.^49^, higher acidification-induced NHE activity has also the potential to release more insulin, a further condition for an increased creatine uptake.

In conclusion, administering a high dose of creatine has been shown to reverse partially cellular stress-induced effects caused by sleep deprivation. Our study showed a maximum effect 4 h after administration.

## Conclusion

Our outcomes show that administering a high single dose of creatine can partially reverse metabolic alterations and fatigue-related cognitive deterioration. The results revise the established assumption that creatine supplementation only works over a longer period. The crucial factor appears to be the increased energy demand of the neuronal cells in combination with an increased extracellular creatine availability. This condition could overcome the main obstacle, namely the marginal intracellular creatine uptake. It can be concluded that creatine has the potential to be used in prolonged cognitive activity during sleep deprivation. Our study showed the effect of a high dose of creatine against sleep deprivation-induced deterioration in cognitive performance, lasting up to 9 h and showing its maximum cognitive effect at 4 h after oral administration. Future research needs to investigate the appropriate dose and determine more accurately the time point at which creatine reaches its maximum effect.

### Supplementary Information

Supplementary Information.

## Data Availability

All data associated with this study are present in the paper. Raw data are available upon request. In case of request please contact (corresponding author): a.gordjinejad@fz-juelich.de.
