# Supplementary material for: Single dose creatine improves cognitive performance and induces changes in cerebral high energy phosphates during sleep deprivation
Source: Sci Rep. 2024 Feb 28;14:4937. doi: 10.1038/s41598-024-54249-9 (PMC10902318; doi:10.1038/s41598-024-54249-9)
Supplement: Supplementary file 1 — Supplementary Information. [file 41598_2024_54249_MOESM1_ESM.docx]

**Supplements**

1. **Material and methods**

**2.2 Experimental procedure**

Anatomical MRI sequences

T_1_-weighted 2D flash sequence were performed for anatomical images of the CSI and PRESS sequences and segmentation of PRESS voxels using following parameters: field of view (FOV) = 20.2 cm × 20.2 cm, voxel size = 0.9 × 0.7 x 4 mm^3^, slices = 35, echo time (TE) = 2.46 ms, repetition time (TR) = 250 ms, averages = 1, acquisition time (TA) = 1.25 min, flip angle = 70 degrees, bandwidth (BW) = 330 Hz/Px and base resolution = 320.

Lastly, for the aim of segmentation of the CSI Grid voxels and to exclude morphological pathology only at the initial run a 3D-MPRAGE sequence was performed using the following parameters: field of view (FOV) = 25.6 cm × 25.6 cm, echo time (TE) = 3.37 ms, repetition time (TR) = 1900 ms, averages = 1, acquisition time (TA) = 3.55 min, TI = 900ms, flip angle = 9°, bandwidth (BW) = 200 Hz/Px, in plane matrix size = 256 × 256, voxel size = 1 × 1 x 1 mm³ and PAT mode = GRAPPA, Accel. factor =1.

All sequence orders and parameters were kept constant throughout all *in vivo* and phantom measurements in both sessions.

**2.5 Statistics**

The weighted mean difference $\bar{MD}$ of a peak integral between BL (6 pm) $x_{i}$and another session $y_{i}$ (0 am, 2 am or 2 am) of a given voxel for *n* (n=15) subjects, was calculated as:

$\bar{MD}=\frac{\sum_{i=1}^{n} w_{i_{xy}}\cdot\left( y_{i}-x_{i} \right)}{\sum_{i=1}^{n} w_{i_{xy}}}$ $\bar{\mathrm{MD}}=\frac{\sum_{i=1}^{n} w_{i_{\mathrm{xy}}}\cdot\left( y_{i}-x_{i} \right)}{\sum_{i=1}^{n} w_{i_{\mathrm{xy}}}}$ $\bar{\mathrm{MD}}=\frac{\sum_{i=1}^{n} w_{i_{\mathrm{xy}}}\cdot\left( y_{i}-x_{i} \right)}{\sum_{i=1}^{n} w_{i_{\mathrm{xy}}}}$

(1)

and the weighted SD of differences (SDD) as:

$SDD=\sqrt{\frac{\left( n-1 \right)\cdot\sum_{i=1}^{n} w_{i_{xy}}\cdot\left[ \left( y_{i}-x_{i} \right)-\bar{MD} \right]^{2}}{n\cdot\sum_{i=1}^{n} w_{i_{xy}}}}$

(2)

Hereby $w_{i_{xy}}=1/\sigma_{i_{xy}}^{2}$ $w_{i_{\mathrm{xy}}}=1/\sigma_{i_{\mathrm{xy}}}^{2}$ with $\sigma_{i_{xy}}^{2}=\sigma_{i_{x}}^{2}+\sigma_{i_{y}}^{2}+2\cdot Cov(x,y)$, $\sigma_{i_{\mathrm{xy}}}^{2}=$is the combined weighting factor of two sessions that considers the variation in spectra quality, while (*σ*_i_) expresses the absolute SD of the calculated fit, processed by Tarquin (Gordji-Nejad et al. 2018).

The weighted mean difference $\bar{MD}$*_Cr_Pl_* for a given voxel of *n* (n=15) subjects administered with creatine (${\Delta_{iCr}=y}_{iCr}-x_{iCr}, w_{i_{xy\_Cr}}$) versus placebo$({\Delta_{iPl}=y}_{iPl}-x_{iPl},w_{i_{xy\_Pl}})$ was calculated as:

$\bar{MD}_{Cr\_Pl}=\frac{\sum_{i=1}^{n} w_{i_{Cr\_Pl} \cdot}\left[ \Delta_{iCr}\cdot({X_{iCr}/X}_{iPl})-\Delta_{iPl} \right]}{\sum_{i=1}^{n} w_{i_{Cr\_Pl}}}$ $\bar{\mathrm{MD}}=\frac{\sum_{i=1}^{n} w_{i_{\mathrm{xy}}}\cdot\left( y_{i}-x_{i} \right)}{\sum_{i=1}^{n} w_{i_{\mathrm{xy}}}}$ , $\bar{\mathrm{MD}}=\frac{\sum_{i=1}^{n} w_{i_{\mathrm{xy}}}\cdot\left( y_{i}-x_{i} \right)}{\sum_{i=1}^{n} w_{i_{\mathrm{xy}}}}$

(3)

(4)

with $w_{i_{Cr\_Pl}}=1/(\frac{1}{w_{i_{xy_{Cr}}}}+\frac{1}{w_{i_{xy_{Pl}}}})$

and the weighted SD of differences (SDD) as:

(5)

$${SDD}_{Cr-Pl}=\sqrt{\frac{\left( n-1 \right)\cdot\sum_{i=1}^{n} w_{i_{Cr\_Pl}}\cdot\left[ \Delta_{iCr}\cdot({X_{iCr}/X}_{iPl})-\Delta_{iPl}-\bar{MD}_{Cr\_Pl} \right]^{2}}{n\cdot\sum_{i=1}^{n} w_{i_{Cr\_Pl}}}}$$

The *T* value is then defined by:

(6)

$T=\sqrt{n}\cdot\frac{\bar{MD}}{SDD}$

The corresponding *p* value was calculated using the *t*, *p* conversion table integrated in Microsoft Excel as tvert(abs(*t*); *n*-2;2), with *n*-2 degrees of freedom and two sides (NIST 1996). Grid averages were calculated without weighting, each region was equally accounted for.

Correlations across n subjects in changes of metabolic parameters and cognitive calculating Spearman correlation coefficient (r) and the T value defined as:

(7)

$$T= \frac{r \cdot\sqrt{n-2}}{\sqrt{1-r^{2}}}$$

**2.6 Segmentation of WM, GM and CSF**

To understand the composition of grey matter (GM), white matter (WM), and cerebrospinal fluid CSF of the 1H-MRS (PRESS) voxels, 1 mm³ 3D-MPRAGE datasets were segmented using the segment routine in default settings provided in SPM12 **Tab.S8** (statistical parametric mapping, The Welcome Trust Centre for Neuroimaging).

Regarding CSI, fractions of WM, GM, and CSF of selected voxels with changes withstanding the Bonferroni corrections were determined using N30R83 atlas-based segmentation of 1 mmT³ 3D-MPRAGE datasets provided by Pmod Neurotool 4.0 (Pmod, Zurich Switzerland). A fixed set of cubic 8 × 8 (25 mm)³ volumes of interest, identical to the CSI grid, was constructed and placed at the exact position displayed by TARQUIN. The desired regional fraction of WM, GM, and CSF was calculated by the intersection with the segmented region. The results, averaged across all subjects, are shown in (**Tab.S8)**.

**2.7 Signal changes due to the displacement of voxels or grids**

The signal measured in a voxel is considered as the sum of contributions from different concentrations of a specific metabolite found in WM, GM, and CSF. Concentration ratios found in the literature are in ranges of 1.18 ≤ GM/WM ≤ 1.87 for tCr, GM/WM = 1.4 for Glu, GM/WM = 0.75 for Pi, 1.05 ≤ GM/WM ≤ 1.22 for PCr, 0.69 ≤ GM/WM ≤ 1 for ATP and for all metabolites ratios of CSF ≤ 0.0007 ( Krukowski 2010; W Pan 1998; Zhu 2004; Loreen Ruhm 2021; Hetherington HP. Spencer DD 2001; Dudley 2014; Y Wang 1998; Nukui 2021; Ågren 1988;).The contribution of signal changes for a given voxel due to the spatial displacement between a time point (0 pm, 2 am or 4 am) and baseline (6pm) were calculated by the sum in fractional change of ΔWM, ΔGM and ΔCSF multiplied by the concentration ratios of the metabolite in the respective fractions. Regarding tCr, PCr, and ATP, the maximum ratio, namely GM/WM = 1.87 for tCr, GM/WM = 1.22 for PCr, and GM/WM = 0.69 for ATP were chosen to calculate the highest possible effect for a given voxel.

1. **Results**
   1. **Metabolic response to SD versus baseline**

**Regional changes**

Regional decrease in PCr/Pi occurred in capsulo-thalamic at 0 pm (right -9.7 ±2.6%, *t*_13_ = -3.74, *p*_13_ = .002; left -13.5 ±2.6%, *t*_13_ = -5.26, *p*_13_ = .0002), 2 am (left -10.7 ±2.0%, *t*_13_ = -4.11, *p*_13_ = .001), 4 am (left -11.6 ±2.9%, *t*_13_ = -2.64, *p*_13_ = .002) and in left corpus callosum (-9.8 ±2.8%, *t*_13_ = -3.52, *p*_13_ = .004).

A decline in ATP-ß/ ^31^P occurred at 0 pm in the left medial central region ( -8.3 ±3.84%, *t*_13_ = -3.84, *p*_13_ = .002).

Pi/^31^P increased in capsulo-thalamic (right 9.0 ±2.4%, *t*_13_ = 3.85, *p*_13_ = .002; left 13.2 ±2.2%, *t*_13_ = 5.97, *p*_13_ = 4.7x10^-5^, at 0pm; left 9.0 ±1.8%, *t*_13_ = 4.96, *p*_13_ = .0003 at 2am; left 11.9 ±3.1%, *t*_13_ = 3.88, *p*_13_ = .002 at 4am). Further increases occurred at 0 pm in the left corpus callosum (7.4 ±2.1%, *t*_13_ = 3.51, *p*_13_ = .004) and left striatum (8.6 ±2.3%, *t*_13_ = 3.66, *p*_13_ = .003) and at 4am in the right posterior lateral parietal region (16.4 ±4.2%, *t*_13_ = 3.39, *p*_13_ = .002).

Glu/tNAA increased during SD, reaching significance at 4 am in the left anterior medial parietal region (19.4 ±7%, *p*_12_ =0.05, *t*_12_ = 2.18) not withstanding the Bonferroni correction (**Table S3)**. No significant changes occurred in PE/^31^P and TCho/^31^P **(Table S5).**

**Global changes that did not withstand the Bonferroni correction**

Changes in HEP were observed throughout all runs after placebo administration. Significant decrease versus baseline occurred in the averaged of the middle grid in PCr/Pi at 0 pm (-5.7 ± 1.8%, *p*_13_ < .01, *t*_13_ = -3.14), 2 am (-4.5 ± 2.1%, *p*_13_ < .04, *t*_13_ = -2.24) and 4 am (-4.1 ± 1.5%, *p*_13_ < .02, *t*_13_ = -2.64) and in ATP-ß/ ^31^P at 0 pm (-4.0 ± 1.5%, *p*_13_ < .02, *t*_13_ = -2.64).

Pi/^31^P significantly increased in the averaged middle grid at 0 pm (5.5 ± 1.2%, *p*_13_ < .001, *t*_13_ = 4.39), 2am (3.8 ± 1.3%, *p*_13_ < .01, *t*_13_ = 2.90) and 4 am (3.5 ± 1.4%, *p*_13_ < .02, *t*_13_ = 2.61).

In the upper grid pH (pH=7.08 at 6pm, 95% Cl, 7.05 ꟷ7.12) dropped by 0.022±0.04, *p*_13_= 0.05, *t*_13_ =-2.12 (95% Cl, 7.03 ꟷ7.09) at 4am**.** Of these changes, only an increase in the averaged middle grid in Pi at 0 pm and a drop in pH level at 0 pm and 4 am withstand the Bonferroni correction. These global response patterns were of differential regional expression withstanding the Bonferroni correction, as shown in **Table S2-S4.**

*Correlation of cognitive and metabolic response*

Increases in PCr/Pi correlated with improvements in SPAN in right temporal transversal (*r*_13_ = 0.71, t_13_=3.46, *p*_13_ = .005, at 4 am), PVT (Speed 0.1 percentile) in right temporal transversal (*r*_13_ = 0.77, t_13_=4.12, *p*_13_ = .001, at 0 pm; *r*_13_ = 0.74, t_13_=3.86, *p*_13_ = .002, at 2 am) and right insula (*r*_13_ = 0.71, t_13_=3.50, *p*_13_ = .004, at 2 am) and numeric in medial premotor (*r*_13_ = 0.74, t_13_=4.0, *p*_13_ = .002 at 0 pm). Increases in ATP-ß/31P correlates with improvements in logic task in right anterior cingulum (*r*_13_ = 0.74, t_13_=4.01, *p*_13_ = .001, at 0 pm), numeric in right anterior F1 (*r*_13_ = 0.69, t_13_=3.46, *p*_13_ = .004 at 4am) and right posterior F1 (*r*_13_ = 0.70, t_13_=3.57, *p*_13_= .003 at 4am). Significant correlations notwithstanding the Bonferroni correction are shown in **Tab.S7 a,b.**

- 1. **Metabolic response to SD after creatine administration**

**Regional** **Changes**

PCr/^31^P increased at 0 pm in bilateral medial central (right 4.1 ± 0.9%, *t*_13_ = 4.72, *p*_13_ = .0004, left 5.3 ± 1.0%, *t*_13_ = 5.3, *p*_13_ = .0001) and decreased in the right occipito-medial region (-4.2 ± 1.1%, *t*_13_ = -3.79, *p*_13_ = .002).

ATP-ß/^31^P decreased at 0 pm in the left medial central (-16.8 ± 2.7%, *t*_13_ = -6.22, *p*_13_ = .00003), left motor (-16.9 ± 4.3%, *t*_13_ = -3.9, *p*_13_ = .002), and right medial premotor region (-12.8 ± 3.7%, *t*_13_ = -3.43, *p*_13_ = .004), at 2 am in the right posterior F1 (-14.6 ± 4.2%, *t*_13_ = -3.48, *p*_13_ = .004) and at 4am in right lateral premotor (-16.7 ± 4.1%, *t*_13_ = -3.96, *p*_13_ = .002), left medial central (-16.1 ± 4.2%, *t*_13_ = -3.59, *p*_13_ = .003) region.

ATP-ß/PCr decreased at 0 pm in right insula (-8.8 ± 2.5%, *t*_13_ = -3.55, *p*_13_ = .004), left medial central (-18.5 ± 3.8%, *t*_13_ = -4.88, *p*_13_ = .0003) and left motor (-20.1 ± 4.9%, *t*_13_ = -4.1, *p*_13_ = .0001) and at 4am in right insula (-10.2 ± 2.4%, *t*_13_ = -4.23, *p*_13_ = .001), right lateral premotor (-17.2 ± 5.3%, *t*_13_ = -3.24, *p*_13_ = .006), bilateral medial central (right -16.7 ± 4.2%, *t*_13_ = -3.99, *p*_13_ = .002, left -21.3 ± 4.9%, *t*_13_ = -4.33, *p*_13_ = .001) and left medial premotor region (-20.3 ± 5.2%, *t*_13_ = -3.89, *p*_13_ = .002).

Supplemented creatine yielded significant increase of cerebral tCr/tNAA versus baseline in the left medial parietal region at 0 pm (3.9 ± 0.2%, *t*_12_ = 2.66, *p*_12_ =0.02) not withstanding the Bonferroni correction **(Table S3).**

**Global changes that did not withstand the Bonferroni correction**

Significant increase in PCr/^31^P versus baseline occurred in the averaged upper grid at 0 pm (2.2 ± 0.9%, *t*_13_ = 2.29, *p*_13_ =0.04) and in Pi/^31^P at 2am (6.8 ± 0.9%, *t*_13_ = 3.02, *p*_13_ =0.01).

Declines occurred in the averaged upper grid in ATP-ß/ ^31^P (-9.2 ± 2.8%, *p*_13_ =0.01, *t*_13_ =-3.25 at 2am) and ATP-ß/PCr (-9.1 ± 3.2%, *p*_13_ =0.01, *t*=-2.87 at 2am).

No significant changes occurred in the pH level, PE/^31^P, and TCho/^31^P (**Table S4, S5**).

Correlation of cognitive and metabolic response

Positive correlation between improvements in cognitive performance and changes in HEP versus baseline revealed withstanding the Bonferroni correction. Increases in PCr/Pi correlates with improvements in WMT in right motor (*r*_13_ = 0.71, t_13_=3.52, *p*_13_ = .004, at 4am), in SPAN in left medial central (*r*_13_ = 0.73, t_13_=3.67, *p*_13_= .003 at 2am), left anterior lateral parietal (*r*_13_ = 0.76, t_13_=4.06, *p*_13_ = .002 at 2am), and left posterior lateral parietal (*r*_13_ = 0.73, t_13_=3.74, *p*_13_ = .003 at 2am) and in PVT in posterior F1 (*r*_13_ = 0.72, t_13_=3.63, *p*_13_ = .003 at 2am). Increases in ATP-ß/31P correlates with improvements in SPAN in left corpus callosum (*r*_13_ = 0.78, t_13_=4.31, *p*_13_ = .001 at 0 pm), in numeric in left anterior cingulum (*r*_13_ = 0.69, t_13_=3.47, *p*_13_ = .004 at 0 pm), and in PVT in right motor (*r*_13_ = 0.73, t_13_=-3.66, *p*_13_= .003 at 0pm). Negative correlations i.e. decreases in ATP-ß/31P and improvements in logic task revealed in left anterior F1 (*r*_13_ = -0.70, t=-3.55, *p*_13_ = .004 at 4am) and left posterior F1 (*r*_13_ = -0.79, t=-4.6, *p* = .0005 at 4am) region.

- 1. **Metabolic response to SD after creatine administration versus placebo**

**Regional changes**

An increase in PCr/^31^P versus placebo occurred at 0pm in the left motor cortex (6.0 ± 0.0%, *t*_13_ = 4.2, *p*_13_ = .001) and at 4am in left medial central (6.1 ± 0.1%, *t*_13_ = 3.59, *p*_13_ = .003), left precnues (6.4 ± 0.1%, *t*_13_ = 2.29, *p*_13_ = .04), and right premotor (6.6 ± 0.1%, *t*_13_ = 5.53, *p*_13_ = .0001) region.

PCr/Pi was significantly increased versus placebo at all three time points regionally in the left capsulo-thalamic at all 3 time points pooled (13.1 ±0.2%, *t*_43_ = 3.91, *p*_43_ = .0003) and in right callosal (8.2 ±0.1%, *t*_43_ = 2.33, *p*_43_ = .04) region at 4am. In the upper grid Δ_VP_PCr/Pi amounted to 0.2% ± 2.6%, reaching significance only at 4 am in right posterior lateral parietal region (25.8 ±0.7%, *t*_13_ = 2.14, *p*_13_ = .05).

The declines in ATP-ß/PCr when pooled at all 3 time points were regionally most pronounced in the left posterior F1 (ATP-ß/ PCr: (-18.7 ±5.2%, *t*_13_ = -3.59, *p*_43_ = .001), left medial central (ATP-ß/ PCr:-18.7 ±5.2%, *t*_43_ = -3.67, *p*_43_ = .001), left lateral premotor (ATP-ß/ PCr: -22.2 ±9.3%, *t*_13_ = -3.99, *p*_43_ = .0003), left motor (ATP-ß/ PCr: -21.2 ±5.8%, *t*_43_= -4.66, *p*_43_= .00001), medial premotor (left ATP-ß/ PCr: -21.5 ±4.1%, *t*_43_= -5.22, *p*_43_= .10^-6^, right:. ATP-ß/ PCr: -13.8 ±4.8%, *t*_43_= -2.89, *p*_43_= .006) and left anterior lateral parietal (ATP-ß/ PCr: -18.1 ±5.6%, *t*_43_= -3.21, *p*_43_= .002) region.

In the middle grid pH level of 7.03 (95% Cl, 7.00 ꟷ7.07) at 6 pm dropped by 0.008±0.033, *p*_13_= 0.32, *t*_13_ =-1.02 (95% Cl, 7.00 ꟷ7.05) at 0pm, by 0.001 ±0.036, *p*_13_= 0.89, *t*_13_ =-0.137 (95% Cl, 7.00 ꟷ7.06) at 2am and 0.01 ±0.043, *p*_13_= 0.37, *t*_13_ =-0.92 (95% Cl, 6.99 ꟷ7.06) at 4am. In the upper grid pH level of 7.08 (95% Cl, 7.05 ꟷ7.1) at 6 pm dropped by 0.012±0.033, *p*_13_= 0.19, *t*_13_ =-1.39 (95% Cl, 7.03 ꟷ7.09) at 0pm, increaed by 0.001±0.035, *p*_13_= 0.87, *t*_13_ = 1.06 (95% Cl, 7.05 ꟷ7.1) at 2am and dropped by 0.005±0.034, *p*_13_= 0.59, *t*_13_ =-5.4 (95% Cl, 7.04 ꟷ7.10) at 4am.

*Correlation of cognitive and metabolic response*

Versus placebo, positive correlation revealed between improvements in numeric task and increases in PCr/Pi in right lateral premotor (*r*_13_ = 0.76, t_13_= 4.25, *p*_13_ = .001 at 2 am; *r*_13_ = 0.75, t_13_= 3.95, *p*_13_ = .002 at 4 am) and negative correlation in temporal medulla (*r*_13_ = -0.75, t_13_=-3.89, *p*_13_ = .002 at 4 am). Furthermore, negative correlation occurred between improvements in PVT and ATP-ß level in temporal medulla (*r*_13_ = -0.73, t_13_=-3.65, *p*_13_ = .003 at 2 am), left posterior lateral parietal (*r*_13_ = -0.82, t_13_=-4.95, *p*_13_ = .0003 at 0 pm), left precuneus (*r*_13_ = -0.74, t_13_=-3.86, *p*_13_ = .002 at 0 pm, *r*_13_ = -0.77, t_13_=-4.18, *p*_13_ = .001 at 2 am) and left anterior F1 (*r*_13_ = -0.80, t_13_=-4.62, *p*_13_ = .001 at 4 am). Significant correlations notwithstanding the Bonferroni correction are shown in **Tab.S7 c,d, Fig.S5.**

**3.4 Signal changes due to the displacement of voxels or grids**

The contribution of signal changes for a given voxel due to the spatial displacement between a time point (0 pm, 2 am or 4 am) and baseline (6pm) were calculated by the sum in fractional change of ΔWM, ΔGM and ΔCSF multiplied by the concentration ratios of the metabolite in the respective fractions (Sup.2.7). Results of changes in 1H-PRESS and CSI for each voxel and time point are shown in (**Tabs. S9-S12**). Highest changes versus baseline and placebo included non-significant ranges of -1.3% ≤ ΔtCr ≤ 1.7%, -1.7 %≤ ΔGlu≤ 1.2%, -1.7% ≤ ΔPCr ≤1.1%, -1.4%≤ ΔATP ≤0.6%, and -1.5%≤ ΔPi 0.9%.

**3.5 Reproducibility, reliability and calibration**

The average between subject variability across all regions, conditions, and subjects was characterized by coefficients of variation of 9.4% for PCr, 15.5% for Pi, 18.9% for ATP-ß, and 8.6% for tCr/NAA. Baseline levels non-significantly deviated in the creatine and placebo session with test-re-test-cv of PCr = -0.6%, Pi = -5.5%, ATP-ß = -1%, tCr/NAA = +1.7%. Phantoms containing 2.5 mM PCr and 2.5 mM ATP-ß were measured in two nights and in three nights those containing 1.6 mM, 2.0 mM, and 2.3 mM of PCr, respectively. Drifts starting from 6 pm to 0 am of ΔPCr = 2.2 ±2.6% and ΔATP-ß = 2.2 ±6.5% were found, to 2 am of ΔPCr = 1.2 ±3.9%, ΔATP-ß = -2.0 ±1.0%, and to 4 am of ΔPCr = 1.6 ±2.4%, ΔATP-ß = 2.2 ±0.8%. Within the invivo meauserments, due to the different z- position from the iso center, a slight shift occurred in the upper grid for PCr/^31^P and ATP-ß/^31^P which were scaled with factors of 1.1 (PCr/^31^P) and 1.3 (ATP/^31^P) for better visual compensation**.**

**Figure S1**


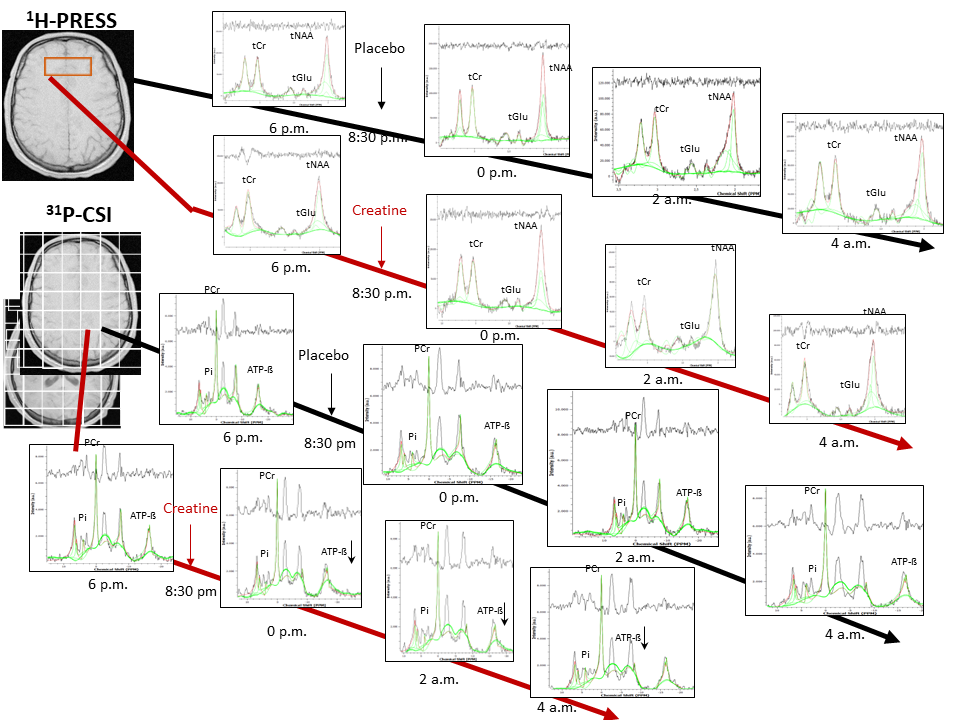


**Fig.S1** Plots of ^1^H and ^31^P spectra over time including the processed signals, fits (in green), baseline and residuals of the frontal located voxel (PRESS) and from one CSI voxel (R6C5) located in medial central region of the upper grid.

**Figure S2**


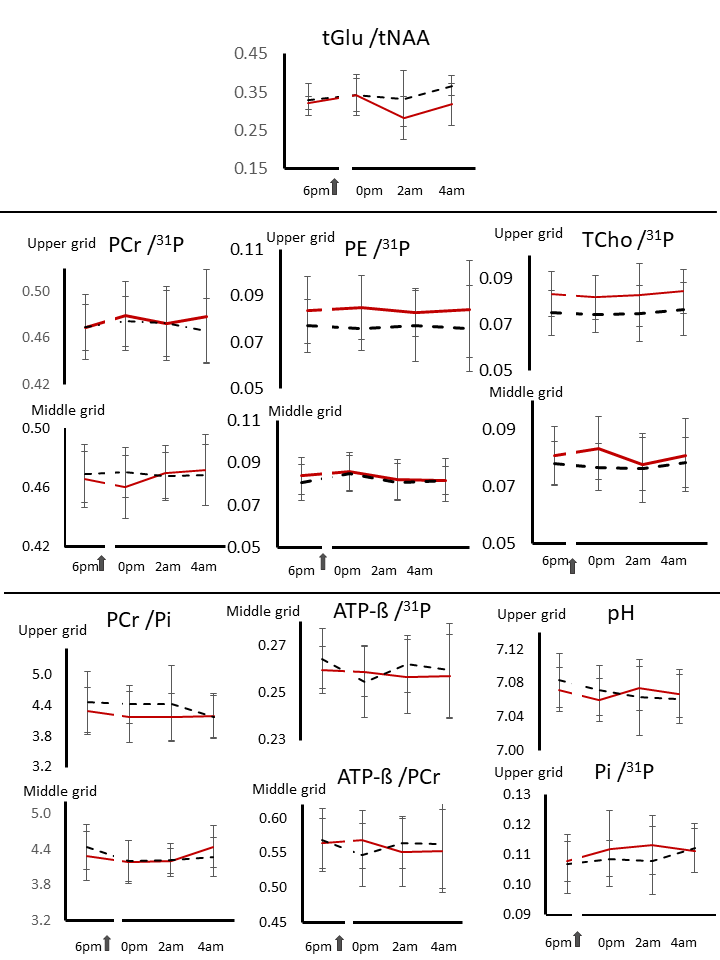


**Fig.S2** **Time courses of** averaged PCr/^31^P, PCr/Pi, Pi/^31^P, ATP-ß/^31^P, PE/^31^P, and TCho/^31^P of middle and upper grid voxels during sleep deprivation under placebo (black dashed lines) and creatine (red solid lines). ^31^P represents the total phosphorus signal, including PCr, Pi, ATP-ß, PE, and TCho. Arrows indicate administration of creatine or placebo at 8:30 pm.

**Figure S3**
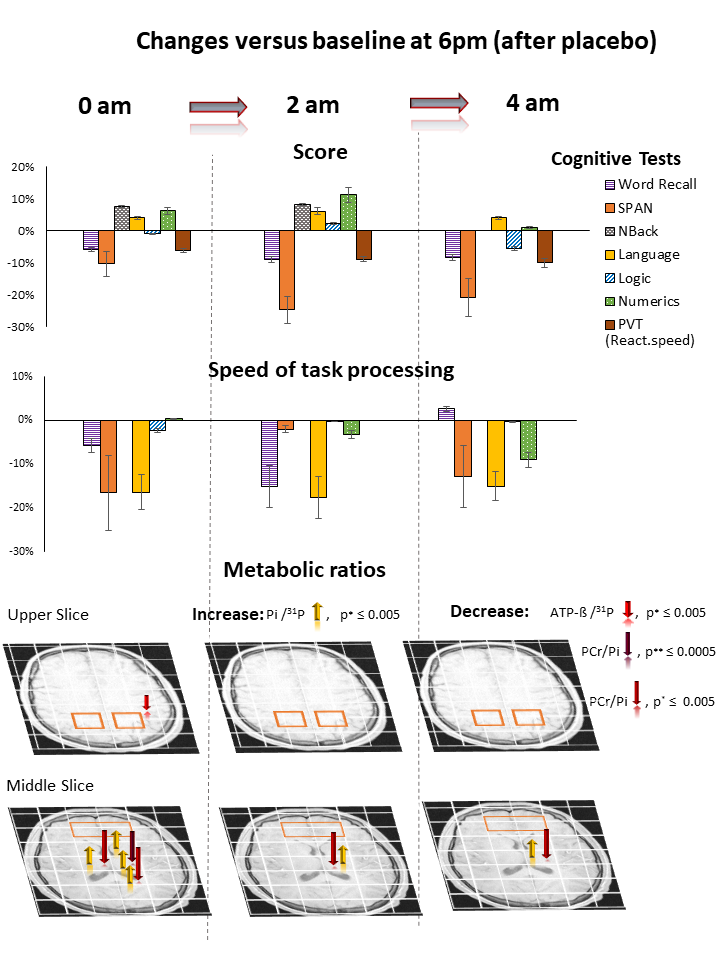


**Fig. S3 Baseline (6 pm) related changes** in cognitive performance, speed in processing time, and voxels yielding significant changes of metabolic parameters after oral administration of placebo (8:30 pm) during sleep deprivation. Sleep deprivation induced significant declines in PCr/Pi (large arrow), ATP-ß/^31^P (red middle arrow) and increase in Pi /^31^P (yellow middle arrow) at 0 pm, 2am and 4am versus baseline (6pm). Significance levels are color coded and indicated by arrows onto axial brain slices in radiological orientation.

**Figure S4**


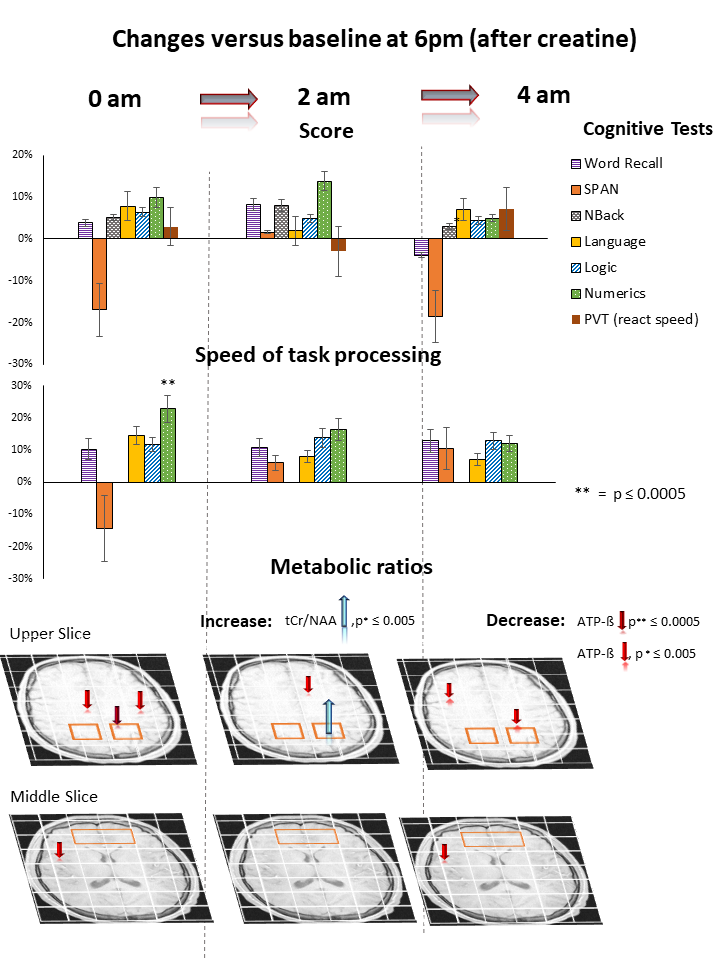


**Fig. S4 Baseline (6 pm) related changes** in cognitive performance, speed in processing time, and voxels yielding significant changes of metabolic parameters after oral administration of creatine (8:30 pm) during sleep deprivation. Creatine led to significant improvements in speed in the processing time of numeric task at 0pm, induced an increase in regional tCr/tNAA (large arrow) at 2am and declines in ATP-ß/31P (middle arrow) at 0 pm, 2am, and 4am versus baseline (6pm). Significance levels are color coded and indicated by arrows onto axial brain slices in radiological orientation.

**Figure S5**


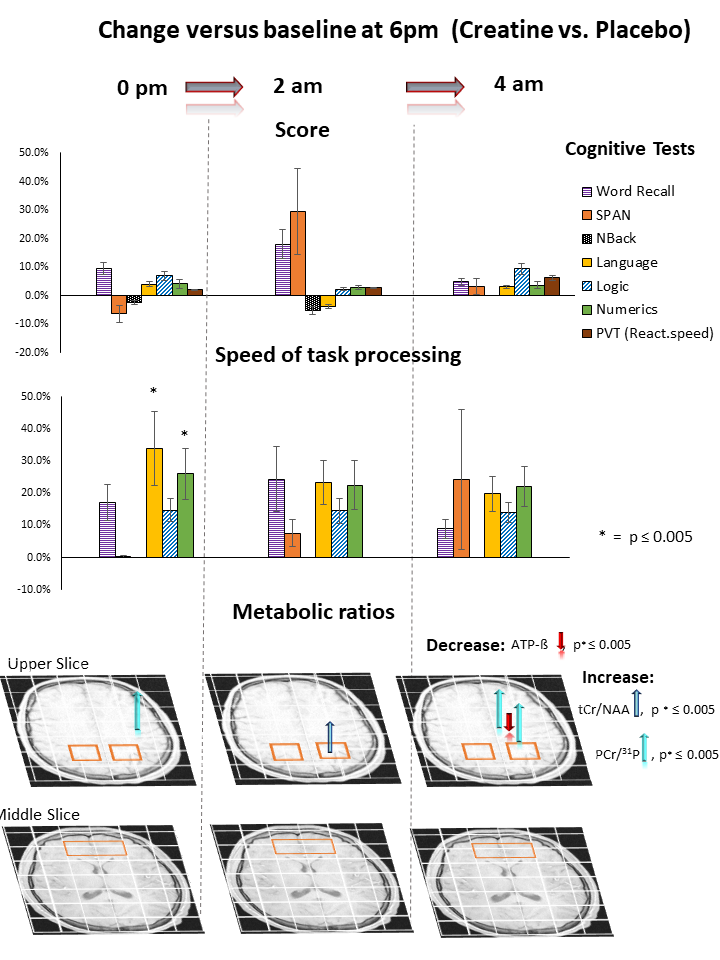


**Fig.S5 Baseline (6 pm) related changes** in cognitive performance, speed in processing time and voxels yielding significant changes of metabolites parameters after oral administration (8:30 pm) of creatine versus placebo. Creatine led to improvements in speed in processing time of language and numeric tasks at 0pm, induced regional increases in PCr at 0pm and 4am and in tCr/tNAA at at 2am and decline in ATP at 4am.Significance levels are color coded and indicated by arrows onto axial brain slices in radiological orientation.

**Figure S6**


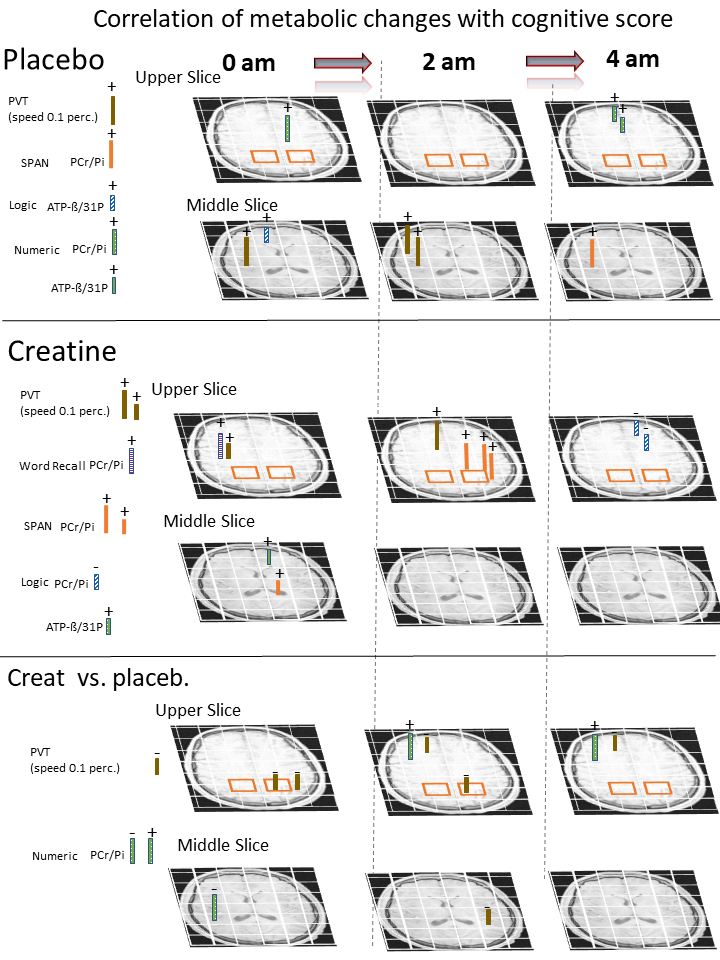


**Fig. S5** Visualization of voxels yielding significant correlations of changes in ΔPCr/Pi and ΔATP-ß/^31^P with cognitive parameters at 0 am, 2 am and 4 am. Positive correlations (+) denotes PCr/Pi or ATP-ß level dependent score, i.e. higher PCr/Pi or ATP-ß are associated with better performance while negative correlations (-) indicates an inverse relation.

**Figure S7**


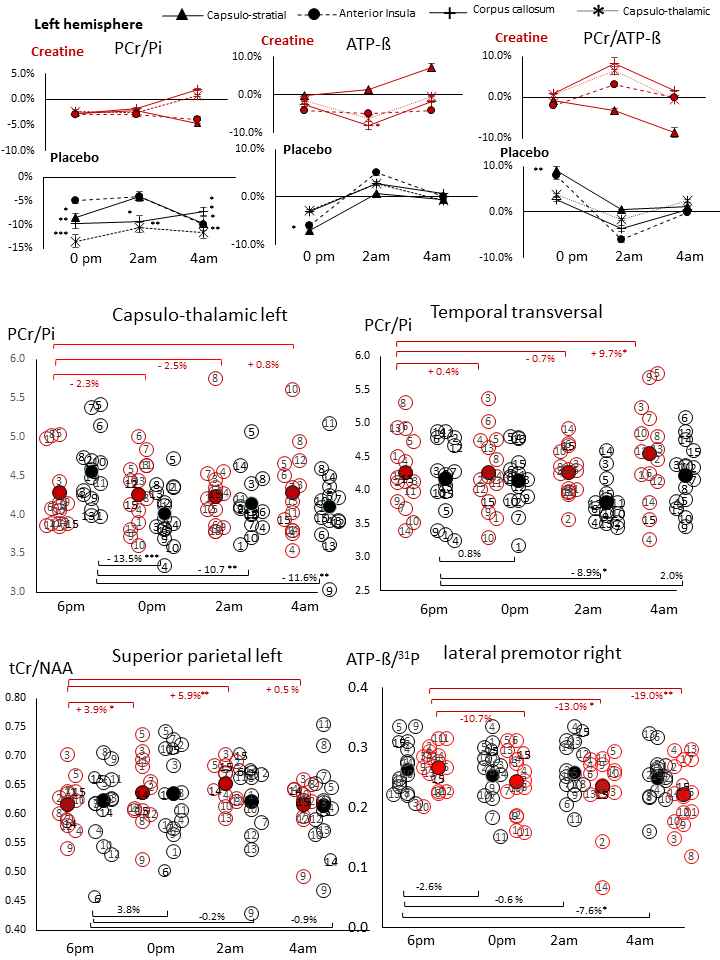


**Fig. S7** Plots of individual ^31^P-MRS-signal ratios PCr/Pi in left capsulo- thalamic and right temporal transversal, tCr/tNAA in left superior parietal and ATP-ß/31P level in the right medial premotor region throughout all sessions in case of placebo (black) or creatine (red). Filled circles indicate average values.

**Table S1.** Results in psychomotor vigilance tests (PVT) in the placebo and creatine session. Two tests of 3 min were conducted each. Trials with reaction times ≥ 850ms were considered as lapses.

| **PVT (Placebo)** | | | | | | | | | | | | | | | | | | | | | | | |
| --- | --- | --- | --- | --- | --- | --- | --- | --- | --- | --- | --- | --- | --- | --- | --- | --- | --- | --- | --- | --- | --- | --- | --- |
|  | **Average react. time**  **w/o lapses** | **Median react. time**  **w/o lapses** | **10% best w/o lapses** | **10% worst**  **w/o lapses** | **Lapses**  **>850ms** | **Reaction speed w/o lapses** | **Average react. time**  **w/lapses** | **0.1 perc. w /lapses** | | **0.25 perc. w /lapses** | | **Median react. time**  **w/ lapses** | | **0.75 perc. w /lapses** | | **0.9 perc. w /lapses** | | **Reaction speed w/lapses** | **0.1 perc. react. speed** | **0.25 perc.**  **react. speed** | **Median reaction speed** | **0.75 perc. react. speed** | **0.9 perc. react. speed** |
| 6pm | 225 ±23 ms | 218 ±21 ms | 188 ±17 ms | 305 ±44 ms | 0 | 4.7 ± 0.5 (1/s) | 227±26 ms | 192 ±18 ms | | 202 ±19 ms | | 218±21 ms | | 239±30 ms | | 273±45 ms | | 4.9±1.2 (1/s) | 3.8±0.5 (1/s) | 4.3±0.5 (1/s) | 4.6±0.4 (1/s) | 5.0±0.4 (1/s) | 5.2±0.4 (1/s) |
| 0 am | 239 ±29 ms | 231 ±28 ms | 198 ±22 ms | 326 ±53 ms | 0.07 | 4.5±1.2 (1/s) | 242 ±34 ms | 203±25 ms | | 213 ±25 ms | | 231 ±28 ms | | 257±37 ms | | 292±49 ms | | 4.45±1.2 (1/s) | 3.5±0.5 (1/s) | 4.0±0.5 (1/s) | 4.4±0.4 (1/s) | 4.7±0.4 (1/s) | 5.0±0.6 (1/s) |
| vers. 6pm | 6.2%**^+^** | 6.0%**^+^** | 4.9%**^+^** | 6.8% |  | -5.2%**^+^** | 6.6%**^+^** | 5.7%**^+^** | | 5.1%**^+^** | | 6%**^+^** | | 7.4%**^+^** | | 7.1% | | -8.7% | -6.4%**^+^** | -6.4%**^+^** | -5.3%**^+^** | -5.3%**^+^** | -4.9%**^+^** |
| 2 am | 245 ±37 ms | 236 ±35 ms | 201 ±28 ms | 336 ±57 ms | 0.07 | 4.4 ± 0.6 (1/s) | 249 ±44 ms | 206 ±28 ms | | 216 ±30 ms | | 237 ±30 ms | | 267±54 ms | | 306±69 ms | | 4.5±0.6 (1/s) | 3.5 ±0.6 (1/s) | 3.9±0.7 (1/s) | 4.3±0.6 (1/s) | 4.7±0.6 (1/s) | 4.9±0.6 (1/s) |
| vers. 6pm | 9%**^+^** | 8.3%**^+^** | 6.6%**^+^** | 9.9%**^+^** |  | -6.7%**^+^** | 9.7%**^+^** | 7.4%**^+^** | | 6.8%**^+^** | | 8.7%**^+^** | | 12%**^+^** | | 12%**^+^** | | -8.5% | -8.5%**^+^** | -8.2%**^+^** | -6.5%**^+^** | -5.4%**^+^** | -7.4%**^+^** |
| 4apm | 248 ±34 ms | 239 ±35 ms | 353 ±46 ms | 306 ±69 ms | 0.07 | 4.3± 0.6(1/s) | 255 ±37 ms | 248 ±34 ms | | 239 ±35 ms | | 240 ±36 ms | | 353±46 ms | | 306±69 ms | | 4.2±0.6 (1/s) | 3.4±0.5 (1/s) | 3.9±0.6 (1/s) | 4.3±0.6 (1/s) | 4.7±0.6 (1/s) | 4.9±0.6 (1/s) |
| vers. 6pm | 10.1%**^+^** | 9.8%**^+^** | 5.9%**^+^** | 15.5%**^+^** |  | -7.5%**^+^** | 12.2**^+^** | 6.5%**^+^** | | 6.8%**^+^** | | 10.2%**^+^** | | 12.8%**^+^** | | 15%**^+^** | | -13.5%**^+^** | -10.2%**^+^** | -9.1%**^+^** | -7.7%**^+^** | -5.4%**^+^** | -13.5%**^+^** |
| **PVT (Creatine)** | | | | | | | | | | | | | | | | | | | | | | | |
|  | **Average react. time**  **w/o lapses** | **Median react. time**  **w/o lapses** | **10% best w/o lapses** | **10% worst**  **w/o lapses** | **Lapses**  **>850ms** | **Reaction speed w/o lapses** | **Average react. time**  **w/lapses** | | **0.1 perc. w /lapses** | | **0.25 perc. w /lapses** | | **Median react. time**  **w/ lapses** | | **0.75 perc. w /lapses** | | **0.9 perc. w /lapses** | **Reaction speed w/lapses** | **0.1 perc. react. speed** | **0.25 perc.**  **react. speed** | **Median reaction speed** | **0.75 perc. react. speed** | **0.9 perc. react. speed** |
| 6pm | 229 ±31 ms | 221 ±30 ms | 189 ±23 ms | 311±54 ms | 0 | 4.7 ± 1 (1/s) | 230 ±32 ms | | 192±23 ms | | 203±25 ms | | 221 ±31 ms | | 246±37 ms | | 276±46 ms | 4.7± 1 (1/s) | 3.7 ± 1 (1/s) | 4.2 ± 1 (1/s) | 4.6 ± 1 (1/s) | 5.0 ± 1 (1/s) | 5.3 ± 1 (1/s) |
| 0 am | 237 ±33 ms | 228 ±30 ms | 193 ±22 ms | 331±52 ms | 0.04 | 4.5 ± 1 (1/s) | 240 ±36 ms | | 196±23 ms | | 209±26 ms | | 229 ±32 ms | | 253±44 ms | | 290±58 ms | 4.8± 1 (1/s) | 3.6 ± 1 (1/s) | 4.1 ± 1 (1/s) | 4.5 ± 1 (1/s) | 4.8 ± 1 (1/s) | 5.1 ± 1 (1/s) |
| vers. 6pm | 3.4% | 3.4% | 2.3% | 6.4% |  | -3.1% | 4% | | 2.3% | | 2.8% | | 3.7% | | 2.9% | | 5.1% | 3% | -3% | -2.5% | -3.7% | -2.9% | -2.7% |
| 2 am | 241 ±35 ms | 231 ±35 ms | 197 ±22 ms | 336 ±59 ms | 0.11 | 4.5 ± 1 (1/s) | 246 ±38 ms | | 201±23 ms | | 213±26 ms | | 232 ±36 ms | | 259±44 ms | | 301±58 ms | 4.6± 1 (1/s) | 3.5 ± 1 (1/s) | 4.4 ± 1 (1/s) | 4.4 ± 1 (1/s) | 4.8 ± 1 (1/s) | 5.0 ± 1 (1/s) |
| vers. 6pm | 5.45%**^+^** | 4.7%**^+^** | 4.3%**^+^** | 8.2% |  | -4.7% | 6.7%**^+^** | | 4.8%**^+^** | | 4.8%**^+^** | | 5%**^+^** | | 5.3%**^+^** | | 9.2%**^+^** | -3% | -6.5%**^+^** | -4.2%**^+^** | -4.5%**^+^** | -4.4%**^+^** | -4.6% |
| 4apm | 242 ±28 ms | 232 ±27 ms | 195 ±18 ms | 343 ±59 ms | 0.07 | 4.4 ± 1 (1/s) | 247±30 ms | | 200±19 ms | | 212±21 ms | | 233 ±28 ms | | 263±39 ms | | 311±58 ms | 5.0± 1 (1/s) | 3.4 ± 1 (1/s) | 3.9 ± 1 (1/s) | 4.4 ± 0.5 (1/s) | 4.8 ± 1 (1/s) | 5.3 ± 1 (1/s) |
| vers. 6pm | 5.9%**^+^** | 5.1%**^+^** | 3.4% | 10.4%* |  | -5.4%**^+^** | 7.2%**^+^** | | 4%**^+^** | | 4.1%* | | 5.5%**^+^** | | 6.7%**^+^** | | 12.7%**^+^** | 7.1% | -8.1%**^+^** | -5.9%**^+^** | -5.6%* | -4.4%**^+^** | -0.3% |

* p= values *p* ≤ 0.005, **^+^** p= values of 0.0063 ≤ *p* ≤ 0.05, that did not survive Bonferroni correction.

**Table S2**

Mean within-subject response to SD versus 6 pm-BL during the placebo and the creatine session. Shown are changes of ratios of PCr and ATP-ß to total phosphorus signal ^31^P (including PCr, Pi. ATP-ß, PE and Tcho) in middle and upper ^31^P-CSI-slice and of tCr and Glu in single voxels (^1^H-PRESS) at 0 pm, 2 am and 4 am.

^1, 2, 3^ Data from only ^1^7 subjects, ^2^10 or ^3^8 subjects were available

* p= values of *p* ≤ 0.005; **^+^** p = values of 0.0063 ≤ *p* ≤ 0.05, that did not survive Bonferroni correction,

| **Middle Slice** | | | | **Δ (PCr/^31^P)** | | | | | |  | **Δ (ATP-ß/ ^31^P)** | | | | | | | | | | | | |
| --- | --- | --- | --- | --- | --- | --- | --- | --- | --- | --- | --- | --- | --- | --- | --- | --- | --- | --- | --- | --- | --- | --- | --- |
| Voxel No | Hs | Anatomical label | | **0 pm vs. 6 pm** | | **2am vs. 6 pm** | | **4 am vs. 6 pm** | |  | **0 pm vs. 6 pm** | | | | **2 am vs. 6 pm** | | | | | **4 am vs. 6 pm** | | | |
|  |  |  |  | **Placebo** | **Creat.** | **Placebo** | **Creat.** | **Placebo** | **Creat.** |  | **Placebo** | | **Creat.** | | **Placebo** | | **Creat.** | | | **Placebo** | **Creat.** | |  |
| R4C3 | r | Anterior insula | | 1% | 4% | 0% | 3% | 0% | 5%**^+^** |  | -5% | | -6%**^+^** | | 2% | | -2% | | -4% | | -5%**^+^** | |  |
| R4C6 | l |  |  | 1% | -2% | -3% | 1% | -1% | 0% |  | -6%**^+^** | | 0% | | 3% | | -1% | | -1% | | 1% | |  |
| R5C3 | r | Temporal transversal | | 2% | 1% | 1% | 2% | 0% | 5%**^+^** |  | -5%**^+^** | | -2% | | -7%**^+^** | | -1% | | -3% | | -4% | |  |
| R5C6 | l |  |  | -2% | 0% | -3% | -2% | -2% | -1% |  | -2% | | 1% | | 5% | | 4% | | 2% | | 2% | |  |
| R6C3 | r | Temporal medulla | | 2% | -1% | 1% | 2% | 0% | 3% |  | -6% | | -1% | | -4% | | -2% | | -3% | | 0% | |  |
| R6C6 | l |  |  | 0% | 1% | -2% | 0% | 2% | 0% |  | -4% | | -2% | | 2% | | -5% | | -2% | | 0% | |  |
| R7C3 | r | Occipito-temporal | | 3% | -3% | 4% | 1% | 1% | 3% |  | -3% | | 4% | | -3% | | -1% | | -2% | | 0% | |  |
| R7C6 | l |  |  | 1% | -4% | 0% | -3% | 2% | -2% |  | -2% | | 1% | | 3% | | -2% | | 1% | | -1% | |  |
| R3C4 | r | Anterior cingulum | | 0% | -2% | 1% | 2% | -2% | 0% |  | -6%* | | -2% | | 2% | | -1% | | -4% | | -3% | |  |
| R3C5 | l |  |  | 1% | 0% | 1% | 3% | -3% | 1% |  | -7%* | | 3% | | -1% | | 4% | | -6%**^+^** | | 1% | |  |
| R4C4 | r | Capsulo-striatal | | 0% | 1% | 1% | 2% | 0% | 1% |  | -7%**^+^** | | -2% | | -4% | | 1% | | -5% | | 0% | |  |
| R4C5 | l |  |  | -1% | -2% | -1% | -2% | -2% | -4%**^+^** |  | -6%**^+^** | | -1% | | -1% | | 2% | | -3% | | 5% | |  |
| R5C4 | r | Thalamo-capsular | | -1% | -3%**^+^** | 1% | 0% | -1% | 0% |  | -4% | | 6%**^+^** | | -5% | | 1% | | -4% | | 1% | |  |
| R5C5 | l |  |  | -2% | 0% | -2% | 2% | -1% | 0% |  | -3% | | -1% | | 0% | | -4% | | -2% | | 1% | |  |
| R6C4 | r | Corpus callosum | | -1% | -2% | 0% | 1% | 1% | 2% |  | -2% | | 3% | | -4% | | -2% | | -3% | | 1% | |  |
| R6C5 | l |  |  | -2% | -1% | -2% | 3% | -1% | 1% |  | -1% | | -1% | | 1% | | -5% | | -1% | | 0% | |  |
| R7C4 | r | Occipito-medial | | 1% | -4%**^+^** | 0% | -1% | 0% | 0% |  | -3% | | 3% | | -3% | | -2% | | -3% | | 1% | |  |
| R7C5 | l |  |  | -1% | -2% | 0% | 0% | 0% | -1% |  | 2% | | 0% | | 3% | | -3% | | 2% | | 2% | |  |
| **Upper Slice** | | | | **0 pm vs. 6 pm** | | **2am vs. 6 pm** | | **4 am vs. 6 pm** | |  | **0 pm vs. 6 pm** | | | | **2 am vs. 6 pm** | | | | | **4 am vs. 6 pm** | | | |
|  |  |  |  | **Placebo** | **Creat.** | **Placebo** | **Creat.** | **Placebo** | **Creat.** |  | **Placebo** | | **Creat.** | | **Placebo** | | **Creat.** | | | **Placebo** | **Creat.** | |  |
| R4C3 | r | | Lateral  premotor | 3% | 1% | -1% | 2% | 2% | 1% |  | -2% | | -10%** | | -1% | | -12%**^+^** | | | -8% | -17%* | |  |
| R4C6 | l | |  | -2% | 1% | -1% | 2% | -4%**^+^** | -1% |  | 15% | | -10% | | 7% | | -16%**^+^** | | | 17% | -3% | |  |
| R5C3 | r | | Motor | 3% | 3% | 1% | 1% | 2% | 3% |  | -9%* | | -17%**^+^** | | -3% | | -7% | | | -9%* | -13%**^+^** | |  |
| R5C6 | l | |  | -3% | 4%**^+^** | -3%* | 3% | -7%**^+^** | 2% |  | 3% | | -17%* | | 3% | | -14%**^+^** | | | 8% | -11% | |  |
| R6C3 | r | | Ant. later. parietal | 2% | 4% | 1% | 1% | 1% | 4% |  | -3% | | -18%**^+^** | | -5% | | -2% | | | -10% | -13%**^+^** | |  |
| R6C6 | l | |  | -1% | 1% | -2% | 3% | -3% | 1% |  | 1% | | -5% | | 1% | | -12%**^+^** | | | 3% | -7% | |  |
| R7C3 | r | | Post. late-ral parietal | 4% | 5% | 0% | 0% | -2% | 3% |  | -6% | | -16%**^+^** | | -4% | | -1% | | | -9% | -14% | |  |
| R7C6 | l | |  | 1% | 4%**^+^** | 3% | 5% | 1% | 5%**^+^** |  | -2% | | -7% | | -6% | | -12% | | | 3% | -13% | |  |
| R3C4 | r | | Anterior F1 | 4% | -3% | 3% | 1% | 2% | -2% |  | -8% | | 4% | | -12%* | | -16%**^+^** | | | -9% | -12% | |  |
| R3C5 | l | |  | 2% | 0% | 0% | 4% | 1% | 0% |  | -10% | | -1% | | -6% | | -21%**^+^** | | | -7% | -15%**^+^** | |  |
| R4C4 | r | | Posterior F1 | 2% | 2% | 0% | 1% | 0% | 1% |  | -2% | | -9%**^+^** | | -6% | | -10%**^+^** | | | -7% | -12%**^+^** | |  |
| R4C5 | l | |  | -1% | 1% | 0% | 2% | -3% | 0% |  | 3% | | -8%**^+^** | | -8% | | -15%* | | | 0% | -10%**^+^** | |  |
| R5C4 | r | | Medial  premotor | 2% | 3% | -1% | 2% | 0% | 4%**^+^** |  | -6%* | | -13%* | | -3% | | -7% | | | -5%* | -17%**^+^** | |  |
| R5C5 | l | |  | -1% | 2% | -1% | 4% | -3% | 4% |  | 1% | | -10%**^+^** | | -1% | | -10%**^+^** | | | 5% | -9%**^+^** | |  |
| R6C4 | r | | Medial central | 2% | 2% | 0% | 2% | 0% | 4%**^*^** |  | -6% | | -13%**^+^** | | -3% | | -6% | | | -4% | -12%**^+^** | |  |
| R6C5 | l | |  | 2% | 3% | 1% | 4% | -1% | 5%* |  | -8%* | | -17%* | | -5% | | -8%**^+^** | | | 0% | -16%* | |  |
| R7C4 | r | | Precuneus | 2% | 4% | 2% | -1% | -1% | 5% |  | -5% | | -13%**^+^** | | -5% | | -3% | | | -4% | -15%**^+^** | |  |
| R7C5 | l | |  | 2% | 1% | 2% | 0% | -2% | 4% |  | 0% | | -5% | | -2% | | -3% | | | 5% | -8% | |  |
| **Δ tCr** | | | | | | | | | |  | **Δ Glu** | | | | | | | | | | | | |
|  | **Anatomical label** | | | 0pm vs 6pm | | 2am vs 6pm | | 4am vs 6pm | |  | 0pm vs 6pm | | | 2am vs 6pm | | | | 4am vs 6pm | | | | | |
|  |  |  |  | placebo | Creat. | placeb | creat. | placebo | Creat. |  | placeb | creat. | | placeb | | creat. | | placeb | | | | creat. |  |
|  | r | | Ant. med. parietal | 2.1% | -2.6% | -0.3% | -4.2% | 2.0% | -10.0% |  | -1.0% | -3.5% | | -8.1% | | -12.8% | | -3.0% | | | | -21.1% |  |
|  | l | |  | -3.4% | -2.9% | -2.2% | -1.6% | -3.8% | 3.7% |  | -2.1% | 0.4% | | 2.0% | | -35.3% | | 12.5% | | | | -31% |  |
|  | Frontal | | | ^1^-12.0% | ^1^1.0% | ^2^-25.7% | ^2^24.4% | ^3^4.4% | ^3^37.2% |  | -  -  -  -  - | | | | | | | | | | | |  |

**Table S3**

Mean within-subject response to SD versus 6 pm-BL during the placebo and the creatine session. Shown are changes of ratios of Pi to total phosphorus signal ^31^P and PCr/Pi in middle and upper ^31^P-CSI-slice and of tCr/NAA and Glu/tNAA in single voxels (^1^H-PRESS) at 0 pm, 2 am and 4 am.

* p= values of *p* ≤ 0.005, ** p= values of *p* ≤ 0.0005, **^+^**p = values of 0.0063 ≤ *p* ≤ 0.05, that did not survive Bonferroni correction,

^1, 2, 3^ Data from only ^1^7 subjects, ^2^10 or ^3^8 subjects were available

| **Middle Slice** | | | | **Δ (Pi/^31^P)** | | | | | | |  | **Δ (PCr/Pi)** | | | | | | | | | | | |
| --- | --- | --- | --- | --- | --- | --- | --- | --- | --- | --- | --- | --- | --- | --- | --- | --- | --- | --- | --- | --- | --- | --- | --- |
| Voxel No | Hs | Anatomical label | | **0 pm vs. 6 pm** | | **2am vs. 6 pm** | | | **4 am vs. 6 pm** | |  | **0 pm vs. 6 pm** | | | | **2 am vs. 6 pm** | | | | | **4 am vs. 6 pm** | | |
|  |  |  |  | **Placebo** | **Creat.** | **Placebo** | | **Creat.** | **Placebo** | **Creat.** |  | **Placebo** | | **Creat.** | | **Placebo** | | **Creat.** | | | **Placebo** | | **Creat.** |
| R4C3 | r | Anterior insula | | 6% | 5%**^+^** | 4% | | 5% | 2% | 3% |  | -4% | | -2% | | -5% | | -2% | | -2% | | | 2% |
| R4C6 | l |  |  | 6%**^+^** | 0% | 3% | | 4% | 10%**^+^** | 6% |  | -5% | | -2% | | -4% | | -3% | | -10%**^+^** | | | -4% |
| R5C3 | r | Temporal transversal | | 2% | 0% | 9%**^+^** | | 1% | -3% | -6% |  | 0% | | 0% | | -9%* | | -1% | | 2% | | | 12%**^+^** |
| R5C6 | l |  |  | 7% | -2% | 4% | | 4% | 6% | 1% |  | -11%**^+^** | | 2% | | -9% | | -5% | | -8% | | | -1% |
| R6C3 | r | Temporal medulla | | 7% | 6% | 7%* | | 1% | -2% | -7% |  | -5% | | -7% | | -6% | | 0% | | 4% | | | 10% |
| R6C6 | l |  |  | 7% | -3% | 4% | | 4% | 6% | -2% |  | -5% | | 5% | | -6% | | -2% | | -3% | | | 2% |
| R7C3 | r | Occipito-temporal | | 2% | 9%**^+^** | 3% | | 2% | -5% | -8%**^+^** |  | 2% | | -12%**^+^** | | 1% | | -1% | | 3% | | | 9% |
| R7C6 | l |  |  | 3% | 1% | -2% | | 2% | 5% | -4% |  | 0% | | 2% | | 1% | | -1% | | -1% | | | 4% |
| R3C4 | r | Anterior cingulum | | 3% | 3% | -1% | | -1% | 3% | 0% |  | -3% | | -5% | | 1% | | 3% | | -2% | | | 3% |
| R3C5 | l |  |  | 3% | 0% | -3% | | 1% | 7% | 5% |  | -2% | | 1% | | 3% | | 2% | | -7% | | | -4% |
| R4C4 | r | Capsulo-striatal | | 8%**^+^** | 4% | 3% | | 1% | 5% | 0% |  | -7%**^+^** | | -4% | | -2% | | 1% | | -3% | | | 0% |
| R4C5 | l |  |  | 9%* | 1% | 1% | | 1% | 9%* | 0% |  | -9%**^+^** | | -3% | | -1% | | -2% | | -10%**^+^** | | | -5% |
| R5C4 | r | Thalamo-capsular | | 9%* | -1% | 7%**^+^** | | 2% | 6%**^+^** | -4% |  | -10%* | | -2% | | -6%**^+^** | | -1% | | -6%**^+^** | | | 3% |
| R5C5 | l |  |  | 13%** | 1% | 9%* | | 5% | 12%* | -1% |  | -13%** | | -2% | | -11%* | | -3% | | -12%* | | | 1% |
| R6C4 | r | Corpus callosum | | 6%**^+^** | 0% | 9%**^+^** | | 2% | 4%**^+^** | -5% |  | -8%**^+^** | | -2% | | -8%**^+^** | | -1% | | -3% | | | 7%**^+^** |
| R6C5 | l |  |  | 7%** | 1% | 8%**^+^** | | 4% | 7%**^+^** | -2% |  | -10%* | | -3% | | -9%**^+^** | | -2% | | -7%**^+^** | | | 2% |
| R7C4 | r | Occipito-medial | | 5% | 2% | 7%**^+^** | | 5% | 1% | -3% |  | -3% | | -8%* | | -9%**^+^** | | -6% | | -1% | | | 2% |
| R7C5 | l |  |  | 1% | -1% | 5% | | 2% | 1% | -1% |  | -3% | | -2% | | -5% | | -3% | | -2% | | | 0% |
| **Upper Slice** | | | | **0 pm vs. 6 pm** | | **2am vs. 6 pm** | | | **4 am vs. 6 pm** | |  | **0 pm vs. 6 pm** | | | | **2 am vs. 6 pm** | | | | | **4 am vs. 6 pm** | | |
|  |  |  |  | **Placebo** | **Creat.** | **Placebo** | | **Creat.** | **Placebo** | **Creat.** |  | **Placebo** | | **Creat.** | | **Placebo** | | **Creat.** | | | **Placebo** | | **Creat.** |
| R4C3 | r | | Lateral  premotor | 3% | 3% | 1% | | 7% | -2% | 7%**^+^** |  | 0% | | 1% | | -3% | | -5% | | | 0% | | -3% |
| R4C6 | l | |  | 2% | 8% | -2% | | 12%**^+^** | 5% | 3% |  | -2% | | -7% | | 6% | | -8% | | | -7% | | -4% |
| R5C3 | r | | Motor | 2% | 9% | 2% | | 8%**^+^** | 2% | 11%**^+^** |  | -1% | | -6% | | 0% | | -6% | | | 1% | | -7% |
| R5C6 | l | |  | -1% | 6% | 4% | | 7% | 8% | 3% |  | -1% | | -5% | | -5% | | -4% | | | -14%**^+^** | | -5% |
| R6C3 | r | | Ant. later. parietal | 5% | 5% | 2% | | 7% | 10% | 9%* |  | -3% | | -6% | | -2% | | -3% | | | -8% | | -3% |
| R6C6 | l | |  | 1% | -3% | 4% | | 6% | 10%**^+^** | 0% |  | -3% | | 1% | | -7% | | -5% | | | -13%**^+^** | | -2% |
| R7C3 | r | | Post. lateral parietal | 1% | 5% | 0% | | 8% | 15%**^+^** | 8% |  | -3% | | -6%**^+^** | | -10% | | -5% | | | -17%**^+^** | | -2% |
| R7C6 | l | |  | 2% | -9%**^+^** | 9% | | 9% | 8% | 5% |  | 0% | | 6% | | -2% | | -5% | | | -4% | | -6% |
| R3C4 | r | | Anterior F1 | 1% | 1% | 1% | | 4% | -2% | 3% |  | -3% | | -3% | | 1% | | -3% | | | -1% | | -3% |
| R3C5 | l | |  | 8% | 2% | 0% | | 7% | 6% | 1% |  | -4% | | -5% | | 3% | | -3% | | | -5% | | 1% |
| R4C4 | r | | Posterior F1 | 1% | 5% | 1% | | 6% | -2% | 4% |  | 2% | | -2% | | 2% | | -3% | | | 1% | | -3% |
| R4C5 | l | |  | 3% | 5% | 0% | | 5% | 5% | 3% |  | -4% | | -4% | | 2% | | -4% | | | -7% | | -2% |
| R5C4 | r | | Medial  premotor | 1% | 5% | 3% | | 6%**^+^** | 1% | 3% |  | 0% | | -4% | | -2% | | -1% | | | -4% | | 3% |
| R5C5 | l | |  | 2% | 7% | 2% | | 7%**^+^** | 3% | 5% |  | -3% | | -5% | | -4% | | -3% | | | -7% | | -3% |
| R6C4 | r | | Medial central | 3% | 8%**^+^** | 4% | | 5% | 8% | 5% |  | -2% | | -7% | | -4% | | -3% | | | -10% | | -1% |
| R6C5 | l | |  | 2% | 4% | 3% | | 6% | 5% | 3% |  | 1% | | -4% | | -3% | | -4% | | | -6% | | -1% |
| R7C4 | r | | Precuneus | 0% | 6% | 0% | | 9% | 12%**^+^** | 9% |  | 3% | | -4% | | 0% | | -8% | | | -11%**^+^** | | -4% |
| R7C5 | l | |  | -1% | 3% | 2% | | 5% | 8% | 4% |  | 1% | | -3% | | -1% | | -7% | | | -9% | | -2% |
| **Δ tCr/tNAA** | | | | | | | | | | |  | **Δ Glu/tNAA** | | | | | | | | | | | |
|  | **Anatomical label** | | | 0 pm vs 6 pm | | 2am vs 6 pm | | | 4am vs 6 pm | |  | 0 pm vs 6 pm | | | 2 am vs 6 pm | | | | 4 am vs 6 pm | | | | |
|  |  |  |  | Placebo | Creat. | Placeb | Creat. | | Placeb | Creat. |  | Placebo | Creat. | | Placeb | | Creat. | | Placeb | | | Creat. | |
|  | r | | Ant. med. parietal | 2.8% | 0.5% | 3.7% | 1.0% | | 2.1% | -0.1% |  | 5.4% | -4.7% | | 11.6% | | -10.0% | | -7.3% | | | -6.1% | |
|  | l | |  | 3.8% | 3.9%**^+^** | -0.2% | 5.3%* | | -0.9% | 0.6% |  | 5.6% | 14.5% | | 3.2% | | -3.3% | | 19.4%^+^ | | | -1.1% | |
|  | Frontal | | | ^1^1.4% | ^1^23.8% | ^2^6.3% | ^2^11.4% | | ^3^0.0% | ^3^-7.1% |  |  | | | | | | | | | | | |

**Table S4**

Mean within-subject response to SD versus 6 pm-BL during the placebo and the creatine session. Shown are changes of ATP-ß/PCr and of pH levels in middle and upper ^31^P-CSI-slice at 0 pm, 2 am and 4 am.

| Middle Slice | | | | **Δ (ATP-ß/PCr)** | | | | | |  | **Δ(pH)** | | | | | | |
| --- | --- | --- | --- | --- | --- | --- | --- | --- | --- | --- | --- | --- | --- | --- | --- | --- | --- |
| Voxel No | Hs | Anatomical label | | 0 pm vs. 6 pm | | 2am vs. 6 pm | | 4 am vs. 6 pm | |  | 0 pm vs. 6 pm | | 2 am vs. 6 pm | | | 4 am vs. 6 pm | |
|  |  |  |  | Placebo | Creat. | Placebo | Creat. | Placebo | Creat. |  | Placebo | Creat. | Placebo | Creat. | | Placebo | Creat. |
| R4C3 | r | Anterior insula | | -6% | -9%* | 2% | -6%**^+^** | -3% | -10%* |  | -0.1% | 0.0% | -0.2% | -0.1% | -0.3% | | -0.1% |
| R4C6 | l |  |  | -7%**^+^** | 2% | 6% | -1% | 1% | 2% |  | -0.1% | -0.3% | -0.2% | -0.2% | -0.3% | | -0.1% |
| R5C3 | r | Temporal transversal | | -8%**^+^** | -2% | -7% | -2% | -2% | -8%**^+^** |  | -0.3% | 0.1% | -0.5%**^+^** | 0.1% | -0.9%* | | -0.1% |
| R5C6 | l |  |  | 0% | 0% | 9% | 5% | 4% | 3% |  | 0.0% | -0.3% | -0.4%**^+^** | -0.2% | -0.4% | | -0.6% |
| R6C3 | r | Temporal medulla | | -8% | 0% | -4% | -4% | -3% | -4% |  | -0.3% | 0.1% | -0.3% | 0.2% | -0.7% | | 0.2% |
| R6C6 | l |  |  | -4% | -2% | 4% | -5% | -2% | 0% |  | -0.2% | -0.3% | -0.7%* | -0.1% | -0.5%**^+^** | | -0.3% |
| R7C3 | r | Occipito-temporal | | -5% | 4%**^+^** | -5% | -1% | -2% | 0% |  | -0.2% | 0.1% | -0.4%**^+^** | 0.2% | -0.5%**^+^** | | 0.0% |
| R7C6 | l |  |  | -2% | 0% | 3% | -1% | 0% | 1% |  | -0.2% | -0.4%**^+^** | -0.3% | -0.3% | -0.7%* | | -0.3% |
| R3C4 | r | Anterior cingulum | | -6%**^+^** | 1% | 0% | -3% | -2% | -3% |  | -0.1% | -0.4% | -0.5%**^+^** | -0.3% | -0.4% | | -0.5% |
| R3C5 | l |  |  | -7% | 2% | -2% | 0% | -3% | -1% |  | -0.4%**^+^** | -0.4% | -0.5%**^+^** | -0.3% | -0.2% | | -0.6%**^+^** |
| R4C4 | r | Capsulo-striatal | | -5% | -2% | -5% | -1% | -5% | 1% |  | 0.0% | -0.3% | -0.5%* | -0.1% | -0.4% | | -0.1% |
| R4C5 | l |  |  | -5% | 2% | -1% | 4% | -1% | 9% |  | 0.1% | -0.1% | -0.4% | -0.1% | -0.3% | | -0.3% |
| R5C4 | r | Thalamo-capsular | | -4% | 7%**^+^** | -6% | 1% | -3% | 1% |  | 0.0% | 0.1% | -0.4%**^+^** | 0.1% | -0.4%**^+^** | | 0.1% |
| R5C5 | l |  |  | -1% | 0% | 2% | -6% | -1% | 1% |  | -0.2% | 0.0% | -0.5%**^+^** | 0.1% | -0.4%**^+^** | | 0.1% |
| R6C4 | r | Corpus callosum | | -2% | 4% | -4% | -2% | -3% | -1% |  | -0.1% | 0.1% | -0.3% | 0.2% | -0.4%**^+^** | | -0.1% |
| R6C5 | l |  |  | 0% | 0% | 3% | -7%**^+^** | 0% | 0% |  | 0.0% | 0.0% | -0.3% | 0.1% | -0.5%**^+^** | | -0.1% |
| R7C4 | r | Occipito-medial | | -4% | 8%**^+^** | -4% | 1% | -4% | 1% |  | -0.2% | 0.1% | -0.1% | 0.2% | -0.4% | | 0.1% |
| R7C5 | l |  |  | 2% | 3% | 2% | -2% | 1% | 3% |  | -0.1% | -0.1% | -0.1% | 0.1% | -0.4%**^+^** | | -0.1% |
| Upper Slice | | | | 0 pm vs. 6 pm | | 2am vs. 6 pm | | 4 am vs. 6 pm | |  | 0 pm vs. 6 pm | | 2 am vs. 6 pm | | | 4 am vs. 6 pm | |
|  |  |  |  | Placebo | Creat. | Placebo | Creat. | Placebo | Creat. |  | Placebo | Creat. | Placebo | Creat. | | Placebo | Creat. |
| R4C3 | r | | Lateral  premotor | -8% | -11% | 0% | -14%**^+^** | -14% | -17%* |  | -0.3% | 0.0% | -0.4%**^+^** | 0.0% | | -0.4% | 0.1% |
| R4C6 | l | |  | 21%**^+^** | -12%**^+^** | 11% | -18%**^+^** | 27%**^+^** | -2% |  | 0.1% | -0.1% | -0.3% | 0.1% | | -0.2% | -0.1% |
| R5C3 | r | | Motor | -12%**^+^** | -21%**^+^** | -4% | -8% | -11%**^+^** | -15%**^+^** |  | -0.2% | -0.4%* | 0.1% | -0.1% | | -0.3% | -0.1% |
| R5C6 | l | |  | 5% | -20%* | 6% | -16%**^+^** | 20%**^+^** | -15% |  | -0.2% | -0.1% | -0.7%* | 0.2% | | -0.3%**^+^** | -0.1% |
| R6C3 | r | | Ant. later. parietal | -4% | -21%**^+^** | -5% | -2% | -11% | -15%**^+^** |  | -0.6%**^+^** | -0.4% | -0.4% | 0.0% | | -0.5% | 0.0% |
| R6C6 | l | |  | 2% | -5% | 5% | -14%**^+^** | 6% | -8%**^+^** |  | 0.2% | 0.0% | -0.1% | 0.3% | | 0.0% | 0.2% |
| R7C3 | r | | Post. lateral parietal | -11% | -17%**^+^** | -6% | 1% | -9% | -17%**^+^** |  | -0.4% | -0.4% | -0.3% | -0.4% | | -0.7%**^+^** | -0.2% |
| R7C6 | l | |  | -2% | -10%**^+^** | -7% | -14%**^+^** | 3% | -17% |  | 0.1% | -0.4% | -0.1% | -0.2% | | 0.0% | -0.1% |
| R3C4 | r | | Anterior F1 | -11% | 7% | -20% | -18%**^+^** | -14% | -4% |  | -0.2% | -0.3% | 0.0% | -0.1% | | -0.1% | -0.5%**^+^** |
| R3C5 | l | |  | -15% | -1% | -9% | -23%**^+^** | -11% | -15% |  | 0.0% | -0.1% | -0.2% | 0.1% | | -0.2% | -0.3% |
| R4C4 | r | | Posterior F1 | -5% | -11%**^+^** | -6% | -11% | -8% | -12%**^+^** |  | -0.1% | 0.0% | -0.2% | 0.2% | | -0.2% | 0.0% |
| R4C5 | l | |  | 5% | -10%**^+^** | -7% | -15%**^+^** | 2% | -11%**^+^** |  | 0.0% | 0.1% | -0.5%**^+^** | 0.3% | | -0.4%**^+^** | 0.1% |
| R5C4 | r | | Medial  premotor | -8%**^+^** | -15%**^+^** | -3% | -9% | -6% | -20%* |  | -0.3% | -0.2% | -0.5%**^+^** | 0.1% | | -0.4%**^+^** | -0.2% |
| R5C5 | l | |  | 3% | -12% | 1% | -13%**^+^** | 10%* | -13% |  | -0.3% | 0.1% | -0.5% | 0.4% | | -0.4% | 0.1% |
| R6C4 | r | | Medial central | -9%**^+^** | -15%**^+^** | -3% | -8% | -5% | -17%* |  | -0.4% | 0.0% | -0.4%**^+^** | 0.0% | | -0.5% | 0.0% |
| R6C5 | l | |  | -10%**^+^** | -19%** | -5% | -11%**^+^** | 2% | -21%* |  | -0.1% | -0.1% | -0.3% | 0.0% | | -0.3% | 0.0% |
| R7C4 | r | | Precuneus | -7% | -14%**^+^** | -9% | 0% | -5% | -17% |  | -0.1% | -0.2% | 0.0% | -0.1% | | -0.4% | -0.1% |
| R7C5 | l | |  | -3% | -2% | -5% | 0% | 7% | -11% |  | -0.2% | -0.5%**^+^** | -0.3% | -0.4% | | -0.2% | 0.0% |

* p= values of *p* ≤ 0.005, ** p= values of *p* ≤ 0.0005, **^+^**p = values of 0.0063 ≤ *p* ≤ 0.05, that did not survive Bonferroni correction.

**Table S5**

Mean within-subject response to SD versus 6 pm-BL during the placebo and the creatine session. Shown are changes of ratios of PE and TCho to total phosphorus signal ^31^P in middle and upper ^31^P-CSI-slice at 0 pm, 2 am and 4 am.

| **Middle Slice** | | | | **Δ (PE/^31^P)** | | | | | |  | **Δ (TCho/^31^P)** | | | | | | |
| --- | --- | --- | --- | --- | --- | --- | --- | --- | --- | --- | --- | --- | --- | --- | --- | --- | --- |
| Voxel No | Hs | Anatomical label | | **0 pm vs. 6 pm** | | **2am vs. 6 pm** | | **4 am vs. 6 pm** | |  | **0 pm vs. 6 pm** | | **2 am vs. 6 pm** | | | **4 am vs. 6 pm** | |
|  |  |  |  | **Placebo** | **Creat.** | **Placebo** | **Creat.** | **Placebo** | **Creat.** |  | **Placebo** | **Creat.** | **Placebo** | **Creat.** | | **Placebo** | **Creat.** |
| R4C3 | r | Anterior insula | | 6% | 3% | -14% | 1% | 4% | 4% |  | -2% | -13% | 0% | -15% | 7% | | -13% |
| R4C6 | l |  |  | 3% | -4% | 1% | -6% | -4% | -12% |  | -1% | 15% | 0% | -1% | 3% | | 3% |
| R5C3 | r | Temporal transversal | | 9% | 2% | 7% | 0% | 10% | -3% |  | -5% | 0% | -3% | -8% | 7% | | -6% |
| R5C6 | l |  |  | 12% | -4% | -1% | -1% | -4% | -6% |  | -1% | 1% | 2% | -5% | 2% | | 3% |
| R6C3 | r | Temporal medulla | | 4% | -3% | 4% | -3% | 9% | -6% |  | -6% | 5% | -7% | -4% | 4% | | -1% |
| R6C6 | l |  |  | 11% | 3% | -1% | 6% | -10% | 1% |  | -7% | 5% | 0% | 7% | -5% | | 4% |
| R7C3 | r | Occipito-temporal | | -1% | -6% | -6% | -3% | 4% | -6% |  | -6% | 3% | -10% | 0% | -3% | | -5% |
| R7C6 | l |  |  | 4% | 9% | -4% | 6% | -9% | 13% |  | -8% | 11% | -2% | 17% | -8% | | 9% |
| R3C4 | r | Anterior cingulum | | 7% | 6% | -13% | 3% | 6% | 13% |  | 9% | 5% | 0% | -7% | 12% | | -5% |
| R3C5 | l |  |  | 4% | -9% | 6% | -13% | 7% | -12% |  | 7% | 3% | -5% | -11% | 16% | | -3% |
| R4C4 | r | Capsulo-striatal | | 11% | 5% | 6% | -1% | 9% | 1% |  | 2% | -7% | 1% | -10% | 2% | | -5% |
| R4C5 | l |  |  | 7% | 8% | 9% | 2% | 5% | 3% |  | 5% | 7% | 2% | -1% | 1% | | 7% |
| R5C4 | r | Thalamo-capsular | | 7% | 3% | 2% | -1% | 5% | -3% |  | 2% | 1% | 0% | -6% | 6% | | 3% |
| R5C5 | l |  |  | 9% | 3% | 2% | 0% | 1% | -6% |  | -4% | -3% | -3% | -7% | -2% | | 1% |
| R6C4 | r | Corpus callosum | | 10% | 4% | 5% | 1% | 5% | -5% |  | -5% | 3% | -8% | -1% | -4% | | -1% |
| R6C5 | l |  |  | 11% | 3% | 0% | -1% | 1% | -7% |  | -5% | 3% | -4% | -6% | -2% | | 1% |
| R7C4 | r | Occipito-medial | | 5% | 7% | 11% | 1% | 6% | 3% |  | -3% | 2% | -12% | 4% | -1% | | -4% |
| R7C5 | l |  |  | 7% | 4% | -5% | 1% | -4% | 0% |  | -8% | 12% | -11% | 10% | -6% | | 7% |
| **Upper Slice** | | | | **0 pm vs. 6 pm** | | **2am vs. 6 pm** | | **4 am vs. 6 pm** | |  | **0 pm vs. 6 pm** | | **2 am vs. 6 pm** | | | **4 am vs. 6 pm** | |
|  |  |  |  | **Placebo** | **Creat.** | **Placebo** | **Creat.** | **Placebo** | **Creat.** |  | **Placebo** | **Creat.** | **Placebo** | **Creat.** | | **Placebo** | **Creat.** |
| R4C3 | r | | Lateral  premotor | -6% | 8% | 5% | -1% | 7% | 16% |  | -10% | -1% | 0% | 7% | | -2% | 4% |
| R4C6 | l | |  | -12% | 7% | -9% | 6% | -8% | 1% |  | -2% | 2% | 8% | 3% | | 0% | 16% |
| R5C3 | r | | Motor | -2% | 7% | 3% | -5% | 3% | -2% |  | -1% | -2% | -3% | 2% | | 4% | 2% |
| R5C6 | l | |  | 5% | 4% | -2% | 2% | 4% | -2% |  | 4% | 2% | 6% | 8% | | 11% | 12% |
| R6C3 | r | | Ant. later. parietal | -4% | 7% | 6% | -7% | 6% | 4% |  | -5% | -4% | -4% | -2% | | 1% | -10% |
| R6C6 | l | |  | 6% | 0% | -2% | -2% | -4% | -3% |  | -1% | 3% | 5% | -3% | | 4% | 8% |
| R7C3 | r | | Post. lateral parietal | -3% | 0% | -2% | -1% | 9% | -2% |  | -4% | -4% | 8% | -6% | | 0% | 4% |
| R7C6 | l | |  | 2% | -3% | -4% | -4% | -8% | -10% |  | -5% | -1% | -9% | -11% | | -12% | 3% |
| R3C4 | r | | Anterior F1 | -3% | 7% | 4% | 13% | 1% | 17% |  | 1% | 4% | -1% | 4% | | 6% | 9% |
| R3C5 | l | |  | -2% | 1% | 3% | 6% | -5% | 15% |  | 0% | -4% | 5% | -1% | | 8% | 8% |
| R4C4 | r | | Posterior F1 | -6% | 2% | 4% | 0% | 8% | 5% |  | -3% | 3% | 4% | 11% | | 7% | 8% |
| R4C5 | l | |  | -1% | 3% | 7% | 8% | 1% | 6% |  | -3% | 2% | 5% | 8% | | 9% | 7% |
| R5C4 | r | | Medial  premotor | -3% | 5% | 3% | -4% | 1% | 4% |  | 3% | -1% | 7% | 4% | | 13% | 4% |
| R5C5 | l | |  | 0% | 3% | 2% | -4% | -1% | -6% |  | -1% | 0% | 1% | 0% | | 7% | -1% |
| R6C4 | r | | Medial central | -1% | 6% | 4% | -5% | 1% | 0% |  | -2% | -1% | 1% | 3% | | 4% | 0% |
| R6C5 | l | |  | 6% | 7% | 4% | -8% | -1% | -1% |  | -1% | 7% | 1% | -4% | | 2% | 1% |
| R7C4 | r | | Precuneus | -1% | 1% | 0% | -4% | -2% | -5% |  | 1% | 0% | -3% | 3% | | 0% | -3% |
| R7C5 | l | |  | -1% | 0% | -4% | -5% | -6% | -8% |  | -7% | 5% | -5% | 6% | | -1% | 0% |

**Table S6**

Mean within-subject response to creatine versus placebo. Shown are changes of Pi and PCr to total phosphorus signal ^31^P (including PCr, Pi. ATP-ß, PE and Tcho) in middle and upper ^31^P-CSI-slice.

* p= values of *p* ≤ 0.005, ** p= values of *p* ≤ 0.0005, **^+^**p = values of 0.0063 ≤ *p* ≤ 0.05, that did not survive Bonferroni correction.

| **Middle Slice** | | | | **Creatine versus placebo** | | | | | | |
| --- | --- | --- | --- | --- | --- | --- | --- | --- | --- | --- |
| Voxel No | Hs | anatomical label | | Pi/P^31^ | | |  | PCr/P^31^ | | |
|  |  |  |  | 0 pm vs. 6 pm | 2am vs. 6 pm | 4 am vs. 6 pm |  | 0 pm vs. 6 pm | 2am vs. 6 pm | 4 am vs. 6 pm |
| R4C3 | r | Anterior insula | | -2% | 0% | -1% |  | 3% | 2% | 4% |
| R4C6 | l |  |  | -6% | 1% | -6% |  | -4% | 3% | 0% |
| R5C3 | r | Temporal transversal | | -2% | -8% | -6% |  | -1% | 0% | 5%**^+^** |
| R5C6 | l |  |  | -9% | 1% | -5% |  | 2% | 1% | 1% |
| R6C3 | r | Temporal medulla | | -1% | -4% | -5% |  | -3% | 2% | 2% |
| R6C6 | l |  |  | -13% | 0% | -8% |  | 0% | 1% | -2% |
| R7C3 | r | Occipito-temporal | | 6% | 0% | 0% |  | -6% | -2% | 0% |
| R7C6 | l |  |  | -7% | 8% | -5% |  | -6% | -4% | -4% |
| R3C4 | r | Anterior cingulum | | -1% | 3% | -4% |  | -4% | 0% | 2% |
| R3C5 | l |  |  | -3% | 2% | -3% |  | -2% | 0% | 2% |
| R4C4 | r | Capsulo-striatal | | -4% | -1% | -7%**^+^** |  | 0% | 0% | 0% |
| R4C5 | l |  |  | -6% | 1% | -8%**^+^** |  | -1% | 0% | -2% |
| R5C4 | r | Thalamo-capsular | | -11%**^+^** | -5% | -12%**^+^** |  | -2% | 0% | 1% |
| R5C5 | l |  |  | -12%**^+^** | -4% | -13%**^+^** |  | 2% | 4% | 1% |
| R6C4 | r | Corpus callosum | | -8%**^+^** | -6% | -11% |  | -2% | 0% | 1% |
| R6C5 | l |  |  | -7% | -3% | -8%**^+^** |  | 1% | 4% | 2% |
| R7C4 | r | Occipito-medial | | -1% | -2% | -6% |  | -6% | -1% | -1% |
| R7C5 | l |  |  | -3% | -5% | -3% |  | -2% | -1% | -1% |
| **Upper Slice** | | | | Pi/P^31^ | | |  | PCr/P^31^ | | |
|  |  |  |  | 0 pm vs.6 pm | 2am vs. 6 pm | 4 am vs. 6 pm |  | 0 pm vs.6 pm | 2am vs. 6 pm | 4 am vs.6 pm |
| R4C3 | r | | Lateral  premotor | -1% | 5% | 5% |  | -3% | 3% | -1% |
| R4C6 | l | |  | 2% | 9% | -9% |  | 2% | 4% | 3% |
| R5C3 | r | | Motor | 8% | 4% | 8% |  | 0% | 1% | 1% |
| R5C6 | l | |  | 5% | 2% | -7% |  | 6% | 4% | 7%**^+^** |
| R6C3 | r | | Ant. Later. Parietal | 4% | 3% | 1% |  | 0% | -1% | 3% |
| R6C6 | l | |  | 0% | 3% | -10% |  | 2% | 5% | 5%**^+^** |
| R7C3 | r | | Post. late-ral parietal | 4% | 6% | -8% |  | 0% | -1% | 5% |
| R7C6 | l | |  | -10% | -1% | -2% |  | 4% | 1% | 4% |
| R3C4 | r | | Anterior F1 | 0% | 4% | 4% |  | -8% | -1% | -3% |
| R3C5 | l | |  | -6% | 6% | 0% |  | -1% | 4% | -1% |
| R4C4 | r | | Posterior F1 | 5% | 4% | 5% |  | -1% | 1% | 2% |
| R4C5 | l | |  | -1% | 2% | -4% |  | 2% | 1% | 4%**^+^** |
| R5C4 | r | | Medial  premotor | 4% | 0% | 3% |  | 1% | 2% | 4% |
| R5C5 | l | |  | 5% | 5% | 3% |  | 2% | 4% | 7%* |
| R6C4 | r | | Medial central | 9% | 1% | -1% |  | 0% | 1% | 4% |
| R6C5 | l | |  | 4% | 3% | -2% |  | 1% | 2% | 6%**^+^** |
| R7C4 | r | | Precuneus | 5% | 6% | -8% |  | 1% | -2% | 5% |
| R7C5 | l | |  | 2% | -3% | -5% |  | -2% | -2% | 6%**^+^** |

**Table S7 a** Pearson’s correlation coefficients (*r_p,_* p-value) between changes in PCr/Pi or tCr/tNAA and cognitive score at 0pm, 2am or 4am under SD. Values in bold represents significances that withstand the Bonferroni correction.

| **ΔPCr/Pi**_SD-BL_  **Middle grid** |  | **PVT- Speed 0.1Pc,** | **WMT** | **Digit Span** | **Language** | **Logic** | **Numeric** | |
| --- | --- | --- | --- | --- | --- | --- | --- | --- |
| **Right hemisphere** |  |  |  |  |  |  |  | |
| Anterior insula | R4 C3 | 0.68,._01, 0pm_  **0.71,._004, 2am_**_._ | ‑ 0.62,._02, 0pm_  ‑ 0.61,._02, 4am_ |  |  |  |  | |
| Temporal transversal | R5 C3 | **0.77,._001, 0pm_**  **0.74,._002, 2am._** | ‑ 0.60,._02, 0pm_  ‑ 0.59,._03, 4am_ | **0.71,._005, 4am_** |  |  |  | |
| Capsulo-thalamic | R5 C4 | 0.59,._02, 0pm_ |  |  | 0.57,._03, 0pm_ |  |  | |
| Temporal medulla | R6 C3 | 0.55,._04, 0pm_ |  |  |  |  | 0.55,._03,2am_ | |
| Corpus callosum | R6 C4 |  | ‑ 0.53,._05, 0pm_ |  |  |  |  | |
| Occipito-temporal | R7 C3 | 0.59,._02, 0pm_ |  |  |  |  |  | |
| Mean |  | 0.54,._05, 2am_ |  |  |  |  |  | |
| **Left hemisphere** |  |  |  |  |  |  |  | |
| Capsulo-striatal | R4 C5 |  |  | ‑ 0.57,._03, 0pm_ |  |  |  | |
| **ΔPCr/Pi**_SD-BL_  **Upper grid** |  | **PVT- Speed 0.1Pc,** | **WMT** | **Digit Span** | **Language** | **Logic** | **Numeric** | |
| **Right hemisphere** |  |  |  |  |  |  |  | |
| Lateral premotor | R4 C3 |  |  |  | -0.54,._04, 0pm_ |  |  |  |
| Posterior F1 | R4 C4 | 0.64,._01, 4am_ |  |  |  |  |  |  |
| Motor | R5 C3 |  |  |  | -0.65,._01, 0pm_ |  |  |  |
| Medial premotor | R5 C4 |  |  |  |  | 0.57,._02, 0pm_ | 0.58,._02, 0pm_ |  |
| Antero-lateral parietal | R6 C3 |  | 0.54,._05, 0pm_  0.70,._01, 2am_ |  |  |  |  |  |
| Medial central | R6 C4 |  | 0.66,._01, 0pm_ |  |  |  |  |  |
| Postero-lat. parietal | R7 C3 |  |  |  |  | 0.55,._03, 0pm_  0.52,._04, 4am_ |  |  |
| Precuneus | R7 C4 |  |  |  |  | 0.53,._04, 0pm_ | 0.54,._04, 0pm_ |  |
| Mean |  | 0.54,._05, 0pm_ |  |  |  |  | 0.61,._02, 0pm_ |  |
| **Left hemisphere** |  |  |  |  |  |  |  |  |
| Posterior F1 | R4 C5 |  |  |  |  |  | 0.51,._05, 0pm_ |  |
| Lateral premotor | R4 C6 |  | -0.53,._05, 0pm_ |  | 0.53,._04, 0pm_ |  |  |  |
| Medial premotor | R5 C5 |  |  |  |  |  | **0.74,._002, 0pm_** |  |
| Motor | R5 C6 |  |  |  | 0.67,._01, 0pm_ |  |  |  |
| Antero-lateral parietal | R6 C6 |  |  |  |  |  |  |  |
| Mean |  |  |  |  |  |  | 0.62,._01, 0pm_ |  |
| **ΔtCr/tNAA**_SD-BL_ |  |  |  |  |  |  |  |  |
| Ant. med. parietal | right |  |  |  |  |  |  |  |
| **KSS** |  |  |  |  | -0.60,._02, 0pm_ |  |  |  |

**Table S7 b** Pearson’s correlation coefficients (*r_p,_* p-value) between changes in ATP-ß and cognitive score at 0pm, 2am or 4am. Values in bold represents significances that withstand the Bonferroni correction.

| **ΔATP-ß/^31^Pi**_SD-BL_  **Middle grid** |  | **PVT- Speed 0.1Pc,** | **WMT** | **Language** | **Logic** | **Numeric** |
| --- | --- | --- | --- | --- | --- | --- |
| **Right hemisphere** |  |  |  |  |  |  |
| Anterior cingulum | R3 C4 |  |  |  | **0.74,._001, 0pm_** | 0.57,._03, 0pm_ |
| Anterior insula | R4 C3 |  |  |  |  | 0.56,._03, 0pm_ |
| Capsulo-striatal | R4 C4 |  |  |  | 0.58,._02, 2am_  0.57,._03, 4am_ | 0.60,._02, 2am_ |
| Temporal transversal | R5 C3 |  |  | 0.59,._02, 2am_ |  |  |
| Temporal medulla | R6 C3 |  |  |  |  |  |
| Occipito-temporal | R7 C3 |  |  |  | 0.50,._02, 0pm_ |  |
| Corpus callosum | R6 C4 |  |  |  |  |  |
| Occipitomedial | R7 C4 | -0.53,._05, 0pm_ |  |  |  |  |
| **Left hemisphere** |  |  |  |  |  |  |
| Anterior cingulum | R3 C5 |  | 0.54,._04, 0pm_ |  |  | 0.58,._02, 2am_ |
| Capsulo-striatal | R4 C5 |  |  | -0.56,._03, 0pm_ | 0.57,._03, 2am_ | 0.51,._02, 2am_ |
| Anterior insula | R4 C6 |  |  |  | 0.64,._01, 0pm_ |  |
| Temporal transversal | R5 C6 |  |  |  | 0.54,._03, 0pm_ |  |
| Corpus callosum | R6 C5 |  |  |  |  |  |
| Temporal medulla | R6 C6 |  |  | -0.57,._03, 0pm_ |  |  |
| Occipito medial | R7 C5 | -0.67,._01, 0pm_ | 0.56,._04, 4am_ |  |  |  |
| Occipito-temporal | R7 C6 |  | 0.70,._01, 0pm_ |  |  |  |
| Mean |  |  |  |  | 0.62,._01, 0pm_ |  |
| **ΔATP/^31^P**_SD-BL_  **Upper grid** |  | **PVT- Speed 0.1Pc,** | **WMT** | **Language** | **Logic** | **Numeric** |
| **Right hemisphere** |  |  |  |  |  |  |
| Anterior F1 | R3 C4 | -0.54,._05, 4am_ | -0.61,._02, 4am_ |  |  | **0.69,._004, 4am_** |
| Lateral premotor | R4 C3 | -0.56,._04, 4am_ |  |  |  | 0.56,._03, 4am_ |
| Posterior F1 | R4 C4 | -0.63,._05, 4am_ |  |  |  | **0.70,._003, 4am_** |
| Medial premotor | R5 C4 | -0.56,._04, 4am_ |  |  |  | 0.59,._02, 4am_ |
| Precuneus | R7 C4 | -0.54,._05, 4am_ |  |  |  |  |
| Mean |  | -0.61,._02, 4am_ |  |  |  |  |
| **Left hemisphere** |  |  |  |  |  |  |
| Anterior F1 | R3 C5 |  |  |  |  | 0.53,._04, 4am_ |
| Posterior F1 | R4 C5 | -0.54,._05, 4am_ |  |  |  |  |
| Lateral premotor | R4 C6 |  | -0.53,._05, 4am_ |  |  |  |
| Precuneus | R7 C5 |  |  | -0.61,._02, 0pm_ |  |  |
| Postero-lat. parietal | R7 C6 |  |  | -0.53,._04, 0pm_ |  |  |

**Table S7 c** Pearson’s correlation coefficients (*r_p,_* p-value) between changes in PCr/Pi and cognitive score after creatine administration under SD at 0pm, 2am or 4am. Values in bold represents significances that withstand the Bonferroni correction.

| **ΔPCr/Pi**_SD-BL_  **Middle grid** |  | **PVT- Speed 0.1Pc,** | **WMT** | **DigitSpan** | **Spatial**  **N-Back** | **Language** | **Logic** |
| --- | --- | --- | --- | --- | --- | --- | --- |
| **Right hemisphere** |  |  |  |  |  |  |  |
| Anterior cingulum | R3 C4 |  |  |  |  | 0.54,._04, 2am_ |  |
| Anterior insula | R4 C3 |  |  | 0.56,._04, 4am_ |  |  |  |
| Temporal transversal | R5 C3 |  |  |  |  |  | -0.62,._01, 4am_ |
| Capsulo-thalamic | R5 C4 |  |  | -0.60,._02, 2am_  0.61,._02, 4am_ |  |  |  |
| Temporal medulla | R6 C3 |  |  | 0.61,._02, 4am_ |  |  |  |
| Corpus callosum | R6 C4 |  |  | -0.53,._05, 2am_ |  |  |  |
| **Left hemisphere** |  |  |  |  |  |  |  |
| Anterior cingulum | R3 C5 |  |  |  |  | 0.56,._03, 2am_ |  |
| Capsulo-striatal | R4 C5 |  |  |  |  |  | -0.56,._04, 2am_ |
| Temporal transversal | R5 C6 |  |  |  | 0.35,._02, pooled_ |  |  |
| Occipito-temporal | R7 C6 |  |  | -0.52,._05, 0pm_ |  |  |  |
| **ΔPCr/Pi**_SD-BL_  **Upper grid** |  | **PVT- Speed 0.1Pc,** | **WMT** | **Digit Span** | **Spatial**  **N-Back** | **Language** | **Logic** |
| **Right hemisphere** |  |  |  |  |  |  |  |
| Lateral premotor | R4 C3 |  | 0.65,._01, 2am_  0.57,._03, 4am_ |  |  |  |  |
| Posterior F1 | R4 C4 | **0.72,._003, 2am_** |  |  |  |  |  |
| Motor | R5 C3 |  | **0.71,._004, 4am_** |  |  |  |  |
| Medial premotor | R5 C4 |  |  | 0.58,._03, 2am_ |  |  |  |
| Medial central | R6 C4 |  |  | 0.68,._01, 2am_ |  |  |  |
| **Left hemisphere** |  |  |  |  |  |  |  |
| Medial premotor | R5 C5 |  |  | 0.60,._02, 2am_ |  |  |  |
| Medial central | R6 C5 |  |  | **0.73,._003, 2am_** |  |  |  |
| Antero-lateral parietal | R6 C6 |  |  | **0.76,._002, 2am_** |  |  |  |
| Precuneus | R7 C5 |  |  | 0.63,._02, 2am_ |  | 0.57,._03, 0pm_ |  |
| Posterior-lateral parietal | R7 C6 |  |  | **0.73,._003, 2am_** |  |  |  |
| Mean |  |  |  | **0.77,._001, 2am_** |  |  |  |
| **KSS** |  |  |  | -0.58,._03, 2am_ |  |  | -0.54,._05, 2am_ |

**Table S7d** Pearson’s correlation coefficients (*r_p,_* p-value) between changes in ATP-ß and cognitive score after creatine administration at 0pm, 2am or 4am. Values in bold represents significances that withstand the Bonferroni correction.

| **ΔATP/^31^Pi**_SD-BL_  **Middle grid** |  | **PVT- Speed 0.1Pc,** | **WMT** | **Digit Span** | **Spatial**  **N-Back** | **Language** | **Logic** | **Numeric** |
| --- | --- | --- | --- | --- | --- | --- | --- | --- |
| **Right hemisphere** |  |  |  |  |  |  |  |  |
| Anterior cingulum | R3 C4 |  | -0.56,._04, 2am_ |  |  | 0.59,._02, 2am_ |  |  |
| Temporal transversal | R5 C3 |  |  | 0.58,._03, 0pm_ |  |  |  |  |
| Capsulo-thalamic | R5 C4 |  |  | 0.69,._01, 0pm_ |  |  |  |  |
| Temporal medulla | R6 C3 |  |  | 0.58,._03, 0pm_ |  |  |  |  |
| Corpus callosum | R6 C4 |  |  |  |  | -0.51,._05, 2am_ |  |  |
| Occipito-temporal | R7 C3 |  |  | 0.56,._04, 0pm_  0.54,._05, 2am_ |  |  |  |  |
| Occipito-medial | R7 C4 |  |  | 0.61,._02, 2am_ |  |  |  |  |
| **Left hemisphere** |  |  |  |  |  |  |  |  |
| Anterior cingulum | R3 C5 |  | -0.60,._02, 0pm_  -0.53,._05, 2am_ |  |  |  |  | **0.69,._004, 0pm_** |
| Capsulo-striatal | R4 C5 |  |  |  |  |  | -0.53,._04, 0pm_ |  |
| Temporal transversal | R5 C6 | -0.70,._01, 0pm_ |  |  |  |  |  |  |
| Corpus callosum | R6 C5 |  |  | **0.78,._001, 0pm_** |  |  |  |  |
| Temporal medulla | R6 C6 | -0.65,._01, 0pm_ |  |  |  |  |  | 0.56,._03, 0pm_ |
| Occipito-medial | R7 C5 |  |  | 0.56,._04, 0pm_  0.59,._03, 2am_ |  |  |  |  |
| Occipito-temporal | R7 C6 |  |  | 0.58,._03, 0pm_ |  |  |  |  |
| Mean |  |  |  | 0.70,._01_ |  |  |  |  |
| **ΔATP/^31^Pi**_SD-BL_  **Upper grid** |  | **PVT- Speed 0.1Pc,** | **WMT** | **Digit Span** | **Spatial**  **N-Back** | **Language** | **Logic** | **Numeric** |
| **Right hemisphere** |  |  |  |  |  |  |  |  |
| Motor | R5 C3 | **0.73,._003, 0pm_** |  |  |  |  | 0.62,._01, 0pm_ |  |
| Medial premotor | R5 C4 | 0.60,._02, 0pm_ |  |  |  |  | 0.56,._03, 0pm_ |  |
| Antero-lateral parietal | R6 C3 |  |  |  |  |  | 0.51,._04, 0pm_ |  |
| Mean |  |  |  |  |  |  | 0.51,._05, 0pm_ |  |
| **Left hemisphere** |  |  |  |  |  |  |  |  |
| Anterior F1 | R3 C5 | **-0.74,._003, 4am_** |  |  |  | -0.53,._04, 0pm_ | **-0.70,._004, 4am_** |  |
| Posterior F1 | R4 C5 |  |  |  |  |  | **-0.79,._0005, 4am_** |  |
|  | R4 C5 |  |  |  |  |  |  | -0.57,._03, 0pm_ |
| Medial premotor | R5 C5 | 0.66,._02, 0pm_ |  |  |  |  |  |  |
| Motor | R5 C6 | 0.54,._05, 0pm_ |  |  |  |  | -0.64,._01, 4am_ |  |
| Lateral premotor | R4 C6 |  |  |  |  |  | -0.62,._01, 4am_ | -0.53,._04, 0pm_ |
|  | R6 C6 |  |  |  |  | -0.54,._04, 0pm_ |  |  |

**Table S8**

Fractions of WM, GM and CSF in 1H PRESS voxels and in CSI with significant changes withstanding the Bonferroni correction extracted from segments of co-registered MPRAGE datasets, averaged across all subjects.

| **Middle Slice** | | | | | | |
| --- | --- | --- | --- | --- | --- | --- |
| Voxel No | Hs | Anatomical label | | **WM** | **GM** | **CSF** |
| R5C3 | r | Temporal transversal | | 0.35 | 0.57 | 0.09 |
| R5C6 | l |  |  | 0.32 | 0.58 | 0.10 |
| R3C4 | r | Anterior cingulum | | 0.55 | 0.40 | 0.05 |
| R3C5 | l |  |  | 0.45 | 0.46 | 0.08 |
| R4C4 | r | Capsulo-striatal | | 0.37 | 0.48 | 0.15 |
| R4C5 | l |  |  | 0.33 | 0.44 | 0.21 |
| R5C4 | r | Thalamo-capsular | | 0.39 | 0.50 | 0.11 |
| R5C5 | l |  |  | 0.36 | 0.50 | 0.15 |
| R6C4 | r | Corpus callosum | | 0.40 | 0.29 | 0.30 |
| R6C5 | l |  |  | 0.38 | 0.28 | 0.33 |
| R7C4 | r | Occipito-medial | | 0.37 | 0.55 | 0.08 |
| R7C5 | l |  |  | 0.32 | 0.55 | 0.12 |
| **Upper Slice** | | | | | | |
| Voxel No | Hs | | Anatomical label | **WM** | **GM** | **CSF** |
| R4C3 | r | | Lateral  premotor | 0.25 | 0.64 | 0.11 |
| R4C6 | l | |  | 0.33 | 0.59 | 0.08 |
| R5C3 | r | | Motor | 0.47 | 0.44 | 0.08 |
| R5C6 | l | |  | 0.52 | 0.42 | 0.07 |
| R6C3 | r | | Ant. later. parietal | 0.40 | 0.48 | 0.12 |
| R6C6 | l | |  | 0.45 | 0.44 | 0.11 |
| R7C3 | r | | Post. lateral parietal | 0.17 | 0.70 | 0.14 |
| R7C6 | l | |  | 0.20 | 0.65 | 0.15 |
| R4C4 | r | | Posterior F1 | 0.54 | 0.39 | 0.07 |
| R4C5 | l | |  | 0.48 | 0.38 | 0.13 |
| R5C4 | r | | Medial  premotor | 0.64 | 0.30 | 0.06 |
| R5C5 | l | |  | 0.61 | 0.31 | 0.09 |
|  | | | | | | |
|  | **Anatomical label** | | | **WM** | **GM** | **CSF** |
|  |  |  |  |  |  |  |
|  | r | | Ant. med. parietal | 0.61 | 0.32 | 0.06 |
|  | l | |  | 0.60 | 0.34 | 0.06 |
|  | Frontal | | | 0.48 | 0.44 | 0.09 |

**Table S9**

Mean within-subject changes in PCr, ATP-ß and tCr signals caused by spatial displacement of the grid position between baseline (6 pm) and 0 pm, 2 am and 4 am. Calculated were the shifts related signal changes in each metabolite due to the different signal contributions in the WM, GM and CSF. Selected are voxels with significant changes withstanding the Bonferroni correction.

| **Middle Slice** | | | | **Δ (PCr)** | | | | | |  | **Δ (ATP-ß)** | | | | | |
| --- | --- | --- | --- | --- | --- | --- | --- | --- | --- | --- | --- | --- | --- | --- | --- | --- |
| Voxel No | Hs | Anatomical label | | **0 pm vs. 6 pm** | | **2am vs. 6 pm** | | **4 am vs. 6 pm** | |  | **0 pm vs. 6 pm** | | **2 am vs. 6 pm** | | **4 am vs. 6 pm** | |
|  |  |  |  | **Placebo** | **Creat.** | **Placebo** | **Creat.** | **Placebo** | **Creat.** |  | **Placebo** | **Creat.** | **Placebo** | **Creat.** | **Placebo** | **Creat.** |
| R5C3 | r | Temporal transversal | | -0.05% | -0.1% | -0.35% | -0.1% | -0.08% | -0.9% |  | -0.01% | 0.0% | -0.03% | -0.1% | -0.11% | -0.4% |
| R5C6 | l |  |  | -0.13% | -0.1% | -0.40% | -0.1% | -0.43% | 0.2% |  | -0.09% | 0.1% | -0.14% | 0.0% | -0.11% | 0.1% |
| R3C4 | r | Anterior cingulum | | 0.20% | 0.1% | -0.12% | -0.1% | -0.06% | -0.1% |  | 0.13% | 0.1% | -0.06% | 0.0% | -0.20% | 0.0% |
| R3C5 | l |  |  | -0.30% | -0.2% | -0.29% | -0.2% | 0.05% | 0.0% |  | -0.17% | -0.1% | -0.04% | -0.4% | -0.09% | -0.3% |
| R4C4 | r | Capsulo-striatal | | -0.21% | 0.3% | 0.08% | 0.2% | -0.07% | -0.4% |  | -0.15% | 0.1% | -0.23% | 0.0% | -0.13% | -0.4% |
| R4C5 | l |  |  | 0.13% | 0.1% | -0.06% | -0.3% | -0.27% | -1.0% |  | 0.17% | -0.2% | 0.04% | -0.3% | -0.01% | -0.4% |
| R5C4 | r | Thalamo-capsular | | -0.47% | -0.1% | -0.07% | 0.1% | -0.27% | -0.7% |  | -0.41% | 0.0% | -0.22% | 0.1% | -0.19% | -0.6% |
| R5C5 | l |  |  | -0.16% | **0.7%** | 0.46% | 0.4% | -0.01% | -0.4% |  | -0.10% | 0.4% | 0.28% | 0.2% | 0.01% | -0.3% |
| R6C4 | r | Corpus callosum | | -0.20% | 0.0% | -0.12% | -0.4% | -0.31% | 0.0% |  | -0.17% | 0.0% | -0.14% | -0.5% | -0.24% | -0.1% |
| R6C5 | l |  |  | 0.27% | -0.7% | 0.33% | -0.8% | -0.10% | -0.7% |  | 0.29% | -0.2% | 0.13% | -0.5% | -0.14% | -0.3% |
| R7C4 | r | Occipito-medial | | -0.28% | -0.1% | -0.25% | -0.1% | -0.76% | 0.0% |  | -0.20% | 0.1% | -0.25% | 0.1% | -0.31% | 0.2% |
| R7C5 | l |  |  | 0.18% | -0.2% | 0.03% | 0.0% | -0.61% | -0.5% |  | 0.17% | -0.2% | -0.24% | -0.1% | -0.69% | -0.3% |
| **Upper Slice** | | | | **0 pm vs. 6 pm** | | **2am vs. 6 pm** | | **4 am vs. 6 pm** | |  | **0 pm vs. 6 pm** | | **2 am vs. 6 pm** | | **4 am vs. 6 pm** | |
|  |  |  |  | **Placebo** | **Creat.** | **Placebo** | **Creat.** | **Placebo** | **Creat.** |  | **Placebo** | **Creat.** | **Placebo** | **Creat.** | **Placebo** | **Creat.** |
| R4C3 | r | | Lateral  premotor | -0.15% | -1.3% | -0.06% | 0.2% | -0.10% | -0.8% |  | -0.12% | -0.5% | -0.12% | 0.1% | -0.10% | -0.5% |
| R4C6 | l | |  | 0.26% | -1.2% | 0.49% | 0.0% | -0.38% | -0.8% |  | 0.13% | -0.6% | 0.15% | -0.2% | -0.21% | -0.5% |
| R5C3 | r | | Motor | -0.46% | -0.3% | -0.09% | -0.4% | -0.15% | -0.3% |  | -0.13% | 0.4% | -0.13% | 0.0% | -0.01% | 0.2% |
| R5C6 | l | |  | -0.15% | 0.2% | -0.01% | 0.0% | -0.39% | -0.2% |  | -0.37% | 0.2% | -0.51% | 0.1% | -0.20% | -0.1% |
| R6C3 | r | | Ant. later. parietal | -0.56% | -0.1% | -0.74% | 0.0% | -0.82% | -0.5% |  | -0.13% | 0.2% | -0.36% | 0.0% | -0.33% | -0.1% |
| R6C6 | l | |  | -0.14% | -0.2% | 0.20% | 0.2% | -0.45% | -0.3% |  | 0.08% | 0.2% | 0.06% | 0.1% | 0.03% | -0.2% |
| R7C3 | r | | Post. lateral parietal | -1.61% | -0.7% | -0.79% | -0.7% | -0.26% | 0.4% |  | -0.91% | -0.3% | -0.46% | -0.4% | -0.14% | 0.3% |
| R7C6 | l | |  | -0.28% | -1.0% | -0.50% | -0.2% | -1.49% | 0.8% |  | -0.11% | -0.4% | -0.25% | -0.1% | -0.85% | 0.2% |
| R4C4 | r | | Posterior F1 | 0.03% | -0.4% | -0.63% | -0.1% | -0.05% | 0.0% |  | 0.00% | -0.3% | -0.61% | -0.1% | 0.15% | 0.0% |
| R4C5 | l | |  | 0.62% | -0.1% | 0.59% | -0.2% | 0.48% | -0.3% |  | 0.35% | 0.5% | 0.27% | 0.0% | -0.01% | -0.1% |
| R5C4 | r | | Medial  premotor | -0.26% | -0.6% | -0.55% | 0.0% | -0.22% | -0.2% |  | -0.41% | -0.7% | -0.78% | 0.0% | -0.18% | -0.1% |
| R5C5 | l | |  | 0.30% | 0.5% | -0.07% | -0.1% | 0.11% | 0.2% |  | 0.26% | 0.6% | -0.30% | -0.1% | 0.04% | 0.1% |

| **Δ (tCr)** | | | | | | | | |
| --- | --- | --- | --- | --- | --- | --- | --- | --- |
| Hs | Anatomical label | **0 pm vs. 6 pm** | | **2am vs. 6 pm** | | **4 am vs. 6 pm** | |  |
|  |  | **Placebo** | **Creat.** | **Placebo** | **Creat.** | **Placebo** | **Creat.** |  |
| r | Ant. med. parietal | -0.4% | -0.2% | -0.1% | 0.2% | 0.3% | 0.8% |  |
| l |  | -0.7% | 0.9% | -1.3% | 0.3% | -0.3% | 0.1% |  |
| Frontal | | -0.5% | 0.5% | 0.5% | 0.7% | 0.7% | -0.3% |  |

| **Spatial displacement (mm)** | | | | | | | | | | | | | |
| --- | --- | --- | --- | --- | --- | --- | --- | --- | --- | --- | --- | --- | --- |
| Middle slice | 0 pm vs. 6 pm | | 2am vs. 6 pm | | 4 am vs. 6 pm | | Upper  slice | 0 pm vs. 6 pm | | 2am vs. 6 pm | | 4 am vs. 6 pm | |
|  | **Placebo** | **Creat.** | **Placebo** | **Creat.** | **Placebo** | **Creat.** |  | **Placebo** | **Creat.** | **Placebo** | **Creat.** | **Placebo** | **Creat.** |
| Δx | 0.9 | 1.0 | 1.0 | 0.6 | 1.1 | 0.8 | Δx | -0.2 | 0.6 | 0.3 | 0.5 | 1.0 | 0.8 |
| Δy | 1.2 | 0.7 | -0.7 | 0.5 | 0.7 | 1.1 | Δy | 1.1 | -0.3 | 1.3 | 1.1 | 1.3 | -0.6 |
| Δz | -0.1 | 1.1 | 0.8 | -0.3 | 1.4 | 0.5 | Δz | 0.5 | 1.2 | 0.6 | 0.2 | 0.7 | 0.7 |

**Table S10**

Spatial displacement of the grid position between each timepoints.

**Table S11**

Mean within-subject changes in Pi and Glu signals caused by spatial displacement of the grid position between baseline (6 pm) and 0pm, 2am and 4am. Calculated were the shifts related signal changes in each metabolite due to the different signal contributions in the WM, GM and CSF. Selected are voxels with significant changes withstanding the Bonferroni correction.

| **Middle Slice** | | | | **Δ (Pi)** | | | | | |  | **Δ (Glu)** | | | | | |
| --- | --- | --- | --- | --- | --- | --- | --- | --- | --- | --- | --- | --- | --- | --- | --- | --- |
| Voxel No | Hs | Anatomical label | | **0 pm vs. 6 pm** | | **2am vs. 6 pm** | | **4 am vs. 6 pm** | |  | **0 pm vs. 6 pm** | | **2 am vs. 6 pm** | | **4 am vs. 6 pm** | |
|  |  |  |  | **Placebo** | **Creat.** | **Placebo** | **Creat.** | **Placebo** | **Creat.** |  | **Placebo** | **Creat.** | **Placebo** | **Creat.** | **Placebo** | **Creat.** |
| R5C3 | r | Temporal transversal | | -0.02% | -0.01% | -0.06% | -0.12% | -0.11% | -0.43% |  | 0.06% | 0.06% | 0.37% | -0.11% | -0.15% | 0.35% |
| R5C6 | l |  |  | -0.09% | 0.10% | -0.16% | 0.02% | -0.14% | 0.15% |  | 0.02% | 0.36% | 0.20% | 0.24% | 0.34% | -0.02% |
| R3C4 | r | Anterior cingulum | | 0.16% | 0.11% | -0.06% | 0.01% | -0.18% | -0.02% |  | 0.06% | 0.16% | 0.02% | 0.15% | -0.39% | 0.16% |
| R3C5 | l |  |  | -0.23% | -0.14% | -0.07% | -0.42% | -0.07% | -0.29% |  | 0.00% | -0.06% | 0.28% | -0.76% | -0.27% | -0.71% |
| R4C4 | r | Capsulo-striatal | | -0.13% | 0.13% | -0.18% | 0.02% | -0.12% | -0.37% |  | -0.05% | -0.13% | -0.60% | -0.23% | -0.21% | -0.28% |
| R4C5 | l |  |  | 0.06% | -0.12% | 0.03% | -0.34% | -0.04% | -0.44% |  | 0.20% | -0.50% | 0.17% | -0.47% | 0.35% | 0.49% |
| R5C4 | r | Thalamo-capsular | | -0.37% | -0.05% | -0.19% | 0.10% | -0.20% | -0.64% |  | -0.31% | -0.03% | -0.40% | 0.04% | -0.09% | -0.56% |
| R5C5 | l |  |  | -0.13% | 0.41% | 0.28% | 0.23% | 0.01% | -0.32% |  | -0.07% | 0.02% | 0.03% | -0.13% | 0.04% | -0.27% |
| R6C4 | r | Corpus callosum | | -0.14% | 0.02% | -0.13% | -0.47% | -0.25% | -0.13% |  | -0.07% | 0.00% | -0.16% | -0.67% | -0.15% | -0.36% |
| R6C5 | l |  |  | 0.23% | -0.26% | 0.14% | -0.51% | -0.14% | -0.38% |  | 0.26% | 0.48% | -0.14% | -0.01% | -0.20% | 0.11% |
| R7C4 | r | Occipito-medial | | -0.20% | 0.08% | -0.23% | 0.05% | -0.36% | 0.17% |  | -0.08% | 0.34% | -0.24% | 0.27% | 0.30% | 0.45% |
| R7C5 | l |  |  | 0.17% | -0.24% | -0.19% | -0.07% | -0.68% | -0.34% |  | 0.13% | -0.33% | -0.56% | -0.21% | -0.80% | -0.06% |
| **Upper Slice** | | | | **0 pm vs. 6 pm** | | **2am vs. 6 pm** | | **4 am vs. 6 pm** | |  | **0 pm vs. 6 pm** | | **2 am vs. 6 pm** | | **4 am vs. 6 pm** | |
|  |  |  |  | **Placebo** | **Creat.** | **Placebo** | **Creat.** | **Placebo** | **Creat.** |  | **Placebo** | **Creat.** | **Placebo** | **Creat.** | **Placebo** | **Creat.** |
| R4C3 | r | | Lateral  premotor | -0.12% | -0.62% | -0.11% | 0.10% | -0.10% | -0.50% |  | -0.17% | -1.60% | -0.04% | 0.26% | -0.09% | -0.96% |
| R4C6 | l | |  | 0.13% | -0.67% | 0.17% | -0.15% | -0.23% | -0.49% |  | 0.23% | -1.43% | 0.57% | 0.02% | -0.44% | -0.96% |
| R5C3 | r | | Motor | -0.19% | 0.37% | -0.12% | -0.02% | -0.03% | 0.08% |  | -0.58% | -0.55% | -0.06% | -0.58% | -0.21% | -0.30% |
| R5C6 | l | |  | -0.35% | 0.18% | -0.42% | 0.08% | -0.22% | -0.10% |  | -0.09% | 0.15% | 0.16% | -0.10% | -0.46% | -0.25% |
| R6C3 | r | | Ant. later. parietal | -0.17% | 0.19% | -0.37% | 0.04% | -0.38% | -0.11% |  | -0.67% | -0.19% | -0.82% | 0.02% | -1.00% | -0.60% |
| R6C6 | l | |  | 0.01% | 0.14% | 0.07% | 0.13% | -0.02% | -0.19% |  | -0.28% | -0.28% | 0.23% | 0.24% | -0.63% | -0.30% |
| R7C3 | r | | Post. lateral parietal | -0.86% | -0.32% | -0.46% | -0.43% | -0.15% | 0.27% |  | -1.63% | -0.88% | -0.85% | -0.82% | -0.30% | 0.39% |
| R7C6 | l | |  | -0.24% | -0.45% | -0.26% | -0.12% | -0.92% | 0.29% |  | -0.39% | -1.21% | -0.56% | -0.30% | -1.73% | 0.99% |
| R4C4 | r | | Posterior F1 | 0.06% | -0.28% | -0.57% | -0.07% | 0.13% | 0.02% |  | 0.07% | -0.50% | -0.60% | -0.05% | -0.13% | -0.02% |
| R4C5 | l | |  | 0.36% | 0.40% | 0.29% | -0.03% | 0.04% | -0.16% |  | 0.65% | -0.32% | 0.66% | -0.25% | 0.66% | -0.43% |
| R5C4 | r | | Medial  premotor | -0.35% | -0.73% | -0.70% | -0.03% | -0.18% | -0.12% |  | -0.17% | -0.60% | -0.43% | -0.03% | -0.23% | -0.18% |
| R5C5 | l | |  | 0.27% | 0.60% | -0.26% | -0.08% | 0.05% | 0.10% |  | 0.31% | 0.46% | 0.01% | -0.08% | 0.14% | 0.23% |

**Table S12**

Mean between-subject changes in PCr, ATP-ß, Pi, Glu and tCr signals caused by spatial displacement of the grid position of creatine versus placebo. Calculated were the shifts related signal changes in each metabolite due to the different signal contributions in the WM, GM and CSF. Selected are voxels with significant changes withstanding the Bonferroni correction.

| **Middle Slice** | | | | | | | | | | | | | | | |
| --- | --- | --- | --- | --- | --- | --- | --- | --- | --- | --- | --- | --- | --- | --- | --- |
| Voxel No | Hs | Anatomical label | **0 pm vs. 6 pm** | | **2am vs. 6 pm** | | **4 am vs. 6 pm** | |  | **0 pm vs. 6 pm** | | **2 am vs. 6 pm** | | **4 am vs. 6 pm** | |
|  |  |  | **Δ PCr** | **Δ ATP-ß** | **Δ PCr** | **Δ ATP-ß** | **Δ PCr** | **Δ ATP-ß** |  | **Δ Pi** | **ΔGlu** | **Δ Pi** | **ΔGlu** | **Δ Pi** | **ΔGlu** |
| R5C3 | r | Temporal transversal | -0.9% | -0.5% | -1.2% | -0.9% | 0.6% | 0.6% |  | -0.5% | -1.0% | -1.0% | -1.4% | 0.6% | 0.6% |
| R5C6 | l |  | -0.3% | 0.0% | -0.6% | -0.3% | -1.1% | -0.8% |  | 0.0% | -0.4% | -0.3% | -0.7% | -0.8% | -1.2% |
| R3C4 | r | Anterior cingulum | 0.5% | 0.4% | 0.1% | 0.1% | 1.0% | 0.8% |  | 0.5% | 0.5% | 0.1% | 0.0% | 0.8% | 1.0% |
| R3C5 | l |  | -0.4% | -0.2% | -1.7% | -1.4% | 0.0% | -0.2% |  | -0.2% | -0.5% | -1.4% | -1.8% | -0.2% | 0.0% |
| R4C4 | r | Capsulo-striatal | -0.1% | -0.1% | 1.1% | 0.8% | 0.1% | 0.1% |  | -0.1% | -0.2% | 0.9% | 1.3% | 0.1% | 0.1% |
| R4C5 | l |  | -0.7% | -0.7% | -0.8% | -0.7% | -0.4% | -0.1% |  | -0.7% | -0.7% | -0.8% | -0.9% | -0.2% | -0.5% |
| R5C4 | r | Thalamo-capsular | 0.6% | 0.5% | 0.8% | 0.7% | -0.7% | -0.6% |  | 0.5% | 0.6% | 0.7% | 0.9% | -0.6% | -0.7% |
| R5C5 | l |  | 0.8% | 0.6% | 0.6% | 0.4% | -0.1% | 0.0% |  | 0.7% | 0.8% | 0.4% | 0.7% | 0.0% | -0.1% |
| R6C4 | r | Corpus callosum | 0.1% | 0.1% | -0.5% | -0.5% | -0.3% | -0.3% |  | 0.1% | 0.1% | -0.5% | -0.5% | -0.3% | -0.3% |
| R6C5 | l |  | 0.7% | 0.7% | 0.9% | 0.7% | 0.7% | 0.6% |  | 0.7% | 0.7% | 0.7% | 1.0% | 0.6% | 0.7% |
| R7C4 | r | Occipito-medial | 1.1% | 0.8% | 0.6% | 0.6% | -0.9% | -0.4% |  | 0.8% | 1.2% | 0.6% | 0.6% | -0.5% | -1.0% |
| R7C5 | l |  | -0.2% | -0.3% | 1.0% | 0.7% | 1.0% | 0.9% |  | -0.3% | -0.1% | 0.7% | 1.1% | 0.9% | 1.0% |
| **Upper Slice** | | | | | | | | | | | | | | | |
| Voxel No | Hs | Anatomical label | **0 pm vs. 6 pm** | | **2am vs. 6 pm** | | **4 am vs. 6 pm** | |  | **0 pm vs. 6 pm** | | **2 am vs. 6 pm** | | **4 am vs. 6 pm** | |
|  |  |  | **Δ PCr** | **Δ ATP-ß** | **Δ PCr** | **Δ ATP-ß** | **Δ PCr** | **Δ ATP-ß** |  | **Δ Pi** | **ΔGlu** | **Δ Pi** | **ΔGlu** | **Δ Pi** | **ΔGlu** |
| R4C3 | r | Lateral  premotor | -1.2% | -0.4% | 0.5% | 0.4% | -0.5% | 0.1% |  | -0.5% | -1.3% | 0.4% | 0.5% | -0.2% | 0.1% |
| R4C6 | l |  | -1.4% | -0.6% | -0.4% | -0.2% | -0.1% | 0.0% |  | -0.8% | -1.6% | -0.2% | -0.5% | 0.0% | 0.0% |
| R5C3 | r | Motor | -0.1% | 0.3% | -0.2% | 0.2% | -0.1% | 0.1% |  | 0.3% | -0.3% | 0.2% | -0.3% | 0.3% | 0.1% |
| R5C6 | l |  | 0.3% | 0.5% | 0.0% | 0.6% | 0.3% | 0.0% |  | 0.5% | 0.2% | 0.6% | -0.2% | 0.2% | 0.0% |
| R6C3 | r | Ant. later. parietal | 0.3% | 0.2% | 0.9% | 0.5% | 0.5% | 0.0% |  | 0.3% | 0.3% | 0.5% | 1.1% | 0.4% | 0.0% |
| R6C6 | l |  | 0.0% | 0.2% | 0.1% | 0.0% | 0.3% | -0.4% |  | 0.2% | 0.0% | 0.0% | 0.1% | -0.1% | -0.4% |
| R7C3 | r | Post. lateral parietal | 0.6% | 0.4% | 0.3% | 0.2% | 0.7% | -0.1% |  | 0.4% | 0.6% | 0.2% | 0.3% | 0.5% | -0.1% |
| R7C6 | l |  | -0.9% | -0.4% | 0.2% | 0.1% | 2.5% | -0.6% |  | -0.4% | -1.0% | 0.1% | 0.3% | 1.5% | -0.6% |
| R4C4 | r | Posterior F1 | -0.4% | -0.2% | 0.6% | 0.6% | 0.2% | -0.1% |  | -0.2% | -0.5% | 0.6% | 0.6% | 0.0% | -0.1% |
| R4C5 | l |  | -0.5% | 0.3% | -0.6% | -0.2% | -0.7% | 0.2% |  | 0.2% | -0.8% | -0.3% | -0.8% | -0.1% | 0.2% |
| R5C4 | r | Medial  premotor | -0.2% | -0.2% | 0.6% | 0.8% | 0.1% | -0.1% |  | -0.2% | -0.2% | 0.7% | 0.5% | 0.1% | -0.1% |
| R5C5 | l |  | 0.0% | 0.2% | 0.0% | 0.2% | 0.1% | 0.0% |  | 0.2% | 0.0% | 0.2% | -0.1% | 0.1% | 0.0% |

| **Δ (tCr)** | | | | | |  |
| --- | --- | --- | --- | --- | --- | --- |
| Hs | Anatomical label | **0 pm vs. 6 pm** | **2am vs. 6 pm** | **4 am vs. 6 pm** |  |  |
| r | Ant. med. parietal | 0.0% | 0.2% | 0.6% |  |  |
| l |  | 1.7% | 1.1% | 0.1% |  |  |
| Frontal | | 1.0% | 1.7% | -0.7% |  | |
